# Erinacenones A–L: Twelve New Isoindolinone Alkaloids from the Edible and Medicinal Mushroom *Hericium erinaceus*

**DOI:** 10.3390/molecules29204901

**Published:** 2024-10-16

**Authors:** Lin-Lin Yuan, Ji-Kai Liu

**Affiliations:** Anhui Province Key Laboratory of Bioactive Natural Products, School of Pharmaceutical Sciences, Anhui University of Chinese Medicine, Hefei 230012, China; yuanlinlin44@ahtcm.edu.cn

**Keywords:** mushroom, *Hericium erinaceus*, Hericiaceae, isoindolin-1-one alkaloids, cytotoxicity

## Abstract

A total of twelve previously unreported isoindolin-1-one compounds, erinacenones A–L (**1**–**12**), were isolated from liquid cultures of the medicinal fungus *Hericium erinaceus*. Their structures were elucidated based on spectroscopic data analysis. The absolute configuration of **12** was determined by comparing its optical rotations with values reported in the literature. The most distinctive feature of these compounds is that their nitrogen atoms are connected to different parts of the special structure moieties. Among them, compounds **3** and **4**, as well as **10** and **11**, are two pairs of isomers differing only by a small change in the position of one double bond. Compounds **4** and **5** were found to show cytotoxic activities, with IC_50_ values of 24.7 and 18.4 μM, respectively, against MCF-7 cell lines.

## 1. Introduction

Mushrooms have been used in traditional medicine for a long time. The mushroom *Hericium erinaceus*, known as ‘Houtou’ in China (and ‘Yamabushitake’ in Japan), is an edible mushroom belonging to the Hericiaceae family of Basidiomycete. It grows on alpine trees. The commercial cultivation of this mushroom is popular worldwide, and it can be cultivated on a large scale using inexpensive substrates such as agricultural wastes. Due to its beneficial health properties, this mushroom is widely used in the diet of East Asian countries [1]. In Chinese and Japanese traditional medicine, this mushroom has been used for centuries as a remedy for gastrointestinal disorders, liver and kidney diseases, and other ailments [1].

Many structurally different and potentially bioactive secondary metabolites of *H. erinaceus* have been discovered in the last decade [2]. The main constituents of *H. erinaceus* are cyathane-type diterpenoids (such as erinacines A–I), steroids (such as erinarols A–F), alkaloids (such as hericirine, fumitremorgin, FD-838, 12β-hydroxyverruculogenTR-2, methylthioglioto, and pseurotin), and polysaccharides (such as α-glucans, β-glucans, and glucan-protein complexes) [3].

Bioactive compounds isolated from the fruiting body or mycelium of *H. erinaceus* have been demonstrated to possess anticancer [4,5], antidiabetic [6], antihyperglycemic [7], hypolipidemic [8], anti-inflammatory [9], antimicrobial [5], and antioxidative properties [10]. Moreover, *H. erinaceus* has been used to treat Alzheimer’s disease [11], cognitive impairments [12], ischemic stroke [13], and Parkinson’s disease. In recent years, the research on *H. erinaceus* has been focused on its antidepressant-like effects for the treatment of depressive disorders [14,15,16].

In order to further explore the presence of other potential new secondary metabolites as well as the biological activities of the mushroom *H. erinaceus*, we carried out a large-scale liquid fermentation. The strains were obtained from Shangri-La, Yunnan, China. A total of twelve previously unreported isoindolin-1-one compounds, erinacenones A–L (**1**–**12**), were isolated from the liquid cultures of the medicinal fungus *H. erinaceus*. The structure elucidation and bioassay results are reported here.

## 2. Results and Discussion

### Structural Elucidation of the Previously Undescribed Compounds

**Figure 1:**
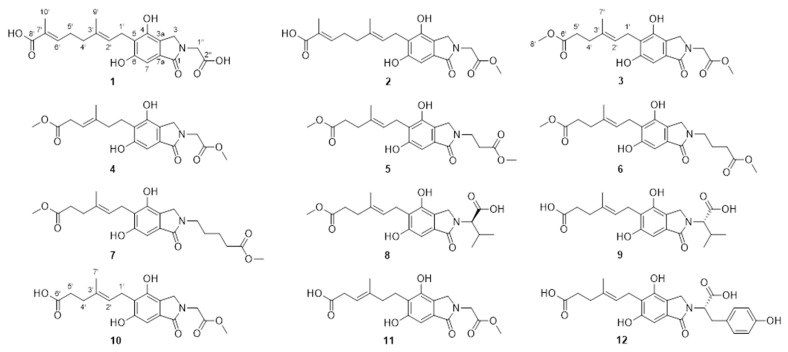
Structures of compounds **1**–**12**.

**Figure 2:**
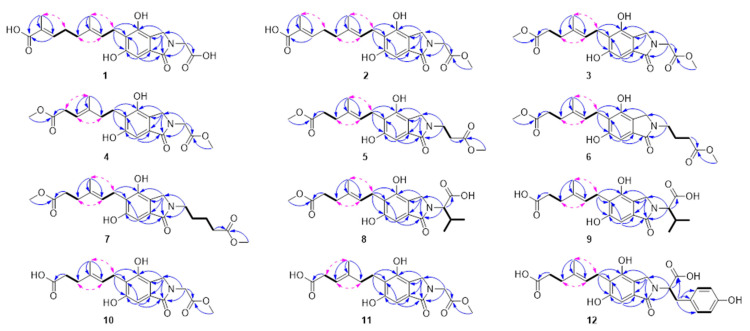
Key 2D NMR correlations for **1**–**12**. Blue lines (HMBC correlations), purple lines (ROESY correlations).

Compound **1** (Figure 1) was obtained as a yellow oil. Based on the molecular ion peak at *m/z* 412.13668 [M + Na]^+^ determined by HRESIMS, it corresponds to the molecular formula C_20_H_23_NO_7_. The ^1^H, ^13^C, and DEPT NMR spectra (Table 1) showed the presence of two methyl groups (*δ*_H_ 1.76, s; 1.82, s; *δ*_C_ 12.4, 16.3), five methylene groups (*δ*_H_ 2.09, t, *J* = 7.3 Hz; 2.28, dd, *J* = 14.9, 7.3 Hz; 3.42, d, *J* = 7.1 Hz; 4.32, s; 4.39, s; *δ*_C_ 39.4, 28.3, 23.6, 45.0, 50.0), three methines, including two olefinic groups (*δ*_H_ 5.30, t, *J* = 7.1 Hz; 6.72, t, *J* = 7.3 Hz; *δ*_C_ 124.5, 143.8), and an aromatic proton (*δ*_H_ 6.76, s; *δ*_C_ 102.0). Additionally, ten quaternary carbons were identified, consisting of five aromatic groups (*δ*_C_ 121.0, 121.7, 131.3, 151.6, 158.0), three carbonyl groups (*δ*_C_ 171.7, 171.8, 173.0), and two olefinic groups (*δ*_C_ 128.8, 134.8). Using the HSQC spectrum, the connectivities of the one-bond ^1^H–^13^C were determined. The HMBC correlations from H-7 (*δ*_H_ 6.76) to C-1 (*δ*_C_ 171.8) and from H-3 (*δ*_H_ 4.39) to C-4 (*δ*_C_ 151.6) confirmed the presence of the isoindoline-1-one substructure (Figure 2). Further HMBC correlations, including those from H-1′ (*δ*_H_ 3.42) to C-4, C-5 (*δ*_C_ 121.7), and C-6 (*δ*_C_ 158.0); H-9′ (*δ*_H_ 1.82) to C-2′ (*δ*_C_ 124.5), C-3′ (*δ*_C_ 134.8), and C-4′ (*δ*_C_ 39.4); H-10′ (*δ*_H_ 1.76) to C-6′ (*δ*_C_ 143.8), C-7′ (*δ*_C_ 128.8), and C-8′ (*δ*_C_ 171.7); and H-1″ (*δ*_H_ 4.32) to C-1, C-3 (*δ*_C_ 50.0), and C-2″ (*δ*_C_ 173.0), together with COSY correlations of H-1′/H-2′ and H-4′/H-5′/H-6′, established the gross structure of **1** as depicted in Figure 2. The H-1′/H-9′, H-2′/H-4′, and H-5′/H-10′ ROESY correlations confirmed the *E* configuration for two double bonds. In light of the comprehensive analysis, the compound was named erinacenone A (see Supplementary Materials).

Compound **2** was obtained as a yellow oil. Based on the molecular ion peak at *m*/*z* 404.17023 [M + H]^+^ determined by HRESIMS, it has the molecular formula C_21_H_25_NO_7_. The ^1^H and ^13^C NMR spectra data (Table 1 and Supplementary Materials) of **2** were extremely similar to **1**, with the primary distinction being the presence of an additional methoxy group (*δ*_C_ 52.8) in **2**. This change was confirmed by the HMBC correlation of H-3″ (*δ*_H_ 3.76, s) to C-2″ (*δ*_C_ 171.2) (Figure 2). The ROESY correlations (H-1′/H-9′, H-2′/H-4′, and H-5′/H-10′) supported the *E* configuration for two double bonds. Thus, the structure of **2** was designated as erinacenone B.

Compounds **3** and **4** were isolated in the form of a yellow oil and shared an identical molecular formula: C_19_H_23_NO_7_. HRESIMS analysis confirmed this molecular formula. When the NMR data (Table 2 and Table 3) from compounds **3** and **4** were compared to those from compound **2**, it was discovered that both compounds had an additional methoxyl group at C-8′ (**3**: *δ*_H_ 3.55, s; *δ*_C_ 51.9; **4**: *δ*_H_ 3.64, s; *δ*_C_ 52.3), while simultaneously losing a trisubstituted double bond and a methyl group, which indicated a structural difference in the side chain at C-5 in the benzene ring. This alteration in the side chain at C-5 was further supported by HMBC correlations (refer to Figure 2). HMBC correlations from H-7′ to C-2′, C-3′, and C-4′, as well as from H-8′ to C-6′, together with COSY correlations of H-4′/H-5′, provided robust evidence for this structural variation. Additionally, the ROESY correlations H-1′/H-7′, H-2′/H-4′, and H-5′/H-7′ confirmed that compounds **3** and **4** are a pair of double-bond positional isomers. Consequently, the structures of **3** and **4** were assigned the names erinacenone C and erinacenone D, respectively.

Compound **5** was isolated as a yellow oil, and its molecular formula was determined to be C_20_H_25_NO_7_ through high-resolution electrospray ionization mass spectrometry (HRESIMS) analysis (*m/z* 392.17017 [M + H]^+^, calculated for C_20_H_26_NO_7_, 392.17038). With the exception of an additional methylene group (*δ*_H_ 2.73, t, *J* = 6.7 Hz; *δ*_C_ 33.9) in **5**, the ^1^H and ^13^C NMR data of **5** (Table 2 and Table 3) closely resembled that of compound **3**. This was confirmed by COSY correlations between H-1″ and H-2″. The H-1′/H-7′ and H-2′/H-4′ ROESY correlations confirmed the *E* configuration. Consequently, the structure of **5** was assigned the name erinacenone E.

Compound **6** was isolated in the form of a yellow oil. Based on the analysis of the NMR data and HRESIMS at *m/z* 406.18594 [M + H]^+^, its molecular formula was determined to be C_21_H_27_NO_7_. In comparison to **5**, it is characterized by the presence of an additional methylene group (*δ*_H_ 2.37, overlapped; *δ*_C_ 32.0). This structural distinction was further supported by HRESIMS data and COSY correlations between H-1″, H-2″, and H-3″. The *E* configuration for the double bond between C-2′ and C-3′ was established by the ROESY correlations of H-1′/H-7′ and H-2′/H-4′. Therefore, the structure of **6** was named erinacenone F.

Compound **7** was isolated in the form of a yellow oil. Based on the analysis of the NMR data and HRESIMS at *m*/*z* 442.18347 [M + Na]^+^, the molecular formula of **7** was determined to be C_22_H_29_NO_7_. The key difference between compounds **7** and **6**, according to NMR data, was that compound **7** included an additional methylene group (*δ*_H_ 2.40, overlapped; *δ*_C_ 34.1). This conclusion was confirmed by HRESIMS data and COSY correlations between H-1″, H-2″, H-3″, and H-4″. As previously mentioned, it was determined that the double bond between C-2′ and C-3′ should be configured. Therefore, the structure of **7** was named erinacenone G.

Compound **8** was obtained as a yellow oil. Based on the analysis of the NMR data and HRESIMS at *m*/*z* 406.18585 [M + H]^+^, it was determined that the molecular formula of **8** was C_21_H_27_NO_7_. Comparing the 1D NMR data (Table 4 and Table 5) of **8** with erinacerin E revealed the absence of signals for a trisubstituted double bond and a methyl group, while an additional methyl group signal (*δ*_H_ 3.55, s; *δ*_C_ 51.9) appeared. This change was confirmed by HMBC correlations from H-8′ to C-6′ (*δ*_C_ 175.8) and HRESIMS data. Detailed analysis of the 2D NMR data (Figure 2) revealed the gross structure of **8**. The *E* configuration was confirmed by the ROESY correlation of H-1′/H-7′ and H-2′/H-4′, as described above. Therefore, the structure of **8** was named erinacenone H (Table 4 and Table 5).

Compound **9**, isolated as a yellow oil, had a molecular formula of C_20_H_25_NO_7_, determined through the analysis of HRESIMS at *m/z* 392.17020 [M + H]^+^, with seven degrees of unsaturation. Structurally similar to **8**, the only difference was the absence of a methyl group, confirmed by the 1D NMR spectra (Table 4 and Table 5) and supported by HRESIMS data. Therefore, **9** was named erinacenone I.

Compounds **10** and **11** were obtained as a yellow oil. The results of the HRESIMS analysis revealed that their molecular formulas were the same: C_18_H_21_NO_7_. The NMR spectra (Table 4 and Table 5) of compounds **10** and **11** resembled those of **3**. Compound **10** was significantly different from **3** in that it lacked a methyl signal. The HRESIMS analysis confirmed the prediction. The double bond configuration between C-2′ and C-3′ was determined as described in compound **3**. Thus, **10** was named erinacenone J. Compounds **10** and **11** were identified as double-bond positional isomers. The ROESY spectrum (H-2′/H-4′ and H-5′/H-7′) established the double-bond configuration between C-3′ and C-4′ for **11**, and it was named erinacenone K.

Compound **12**, isolated as a yellow oil, had a molecular formula of C_24_H_25_NO_8_ (*m*/*z* 456.16529 [M + H]^+)^, determined through HRESIMS analysis, with twelve degrees of unsaturation. The 1D NMR spectra of **12** revealed that it and **11** both included the (*E*)-6-(4,6-dihydroxy-1-oxoisoindolin-5-yl)-4-methylhex-4-enoate moiety. The key distinction was the substitution of 3-(4-hydroxyphenyl) propionic acid for 3-methylbutyric acid at the nitrogen atom. This result was supported by the COSY correlations of H-1″/H-2″, H-4″/H-5″, and H-7″/H-8″; the HMBC correlations from H-1″ (*δ*_H_ 5.10, dd, *J* = 11.2, 4.7 Hz) to C-1 (*δ*_C_ 171.8), C-3 (*δ*_C_ 47.0), and C-9″ (*δ*_C_ 174.5) and from H-2″ (*δ*_H_ 3.11, dd, *J* = 14.6, 11.4 Hz; 2.25, overlapped) to C-3″ (*δ*_C_ 129.3), C-4″ (*δ*_C_ 130.6), and C-8″ (*δ*_C_ 130.6); as well as the HRESIMS data. The double-bond configuration between C-2′ and C-3′ was established based on the ROESY spectrum. Therefore, the structure of **12** was named erinacenone L.

By comparing the optical rotations of compounds **8**, **9**, and **12** with those of the synthetic phthalimidines, the absolute configuration of C-1″ was determined [17]. Compounds **9** and **12** showed negative specific optical rotations, indicating that they possess the *S* configuration at C-1″. Compound **8** showed a positive specific rotation, indicating that it possesses the *R* configuration at C-1″.

## 3. Materials and Methods

### 3.1. General Experimental Procedures

The optical rotations were recorded using a Rudolph AutopolIV-T polarimeter (Rudolph, Hackettstown, NJ, USA). UV spectra were obtained using a UH5300 spectrophotometer (Hitachi, Kyoto, Japan). NMR spectra were obtained using a Bruker Avance III 600 MHz or Avance Neo 500 MHz spectrometer (Bruker, Karlsruhe, Germany). HRESIMS data were collected using a Q-Exactive Orbitrap mass spectrometer (Thermo Scientific, Waltham, MA, USA). Sephadex LH-20 (Amersham Biosciences, Uppsala, Sweden) and silica gel (Qingdao Haiyang Chemical Co., Ltd., Qingdao, China) were used for column chromatography (CC). Medium-pressure liquid chromatography (MPLC) was performed on a Interchim PuriFlash 450 instrument (Interchim, Montluçon, France). An Agilent 1260 liquid chromatography system with a DAD detector (Agilent Technologies, Santa Clara, CA, USA) was used for preparative HPLC using a Zorbax SB-C18 column (9.4 × 150 mm, 5 µm, 4 mL·min^−1^). An Agilent 1260 liquid chromatograph with a Zorbax SB-Aq column (4.6 × 250 mm, 5 µm, 1 mL·min^−1^) was used for semipreparative HPLC. TLC was performed on GF254 plates (Qingdao Marine Chemical Inc., Qingdao, China).

### 3.2. Fungal Material

The fungus *H. erinaceus* (accession No. KU855351.1) was collected in August 2007 in Shangri-La County, Yunnan province, China, and was identified by Prof. Zang (Kunming Institute of Botany, CAS, Kunming, China) at the Kunming Institute of Botany. The strain was deposited at South-Central Minzu University. The strain of *H. erinaceus* was cultured for 30 days at 24 °C on a rotary shaker at 150 rpm on liquid medium (glucose 5%, yeast extract 0.4%, peptone 0.15%, KH_2_PO_4_ 0.05%, MgSO_4_ 0.05%, pH 6.5).

### 3.3. Extraction and Isolation

A 100 L culture broth filtrate was concentrated to 10 L, then extracted with ethyl acetate. Mycelium was extracted with acetone using wall-breaking extraction. The combined extracts were concentrated to yield a crude extract (110.98 g). Silica gel column chromatography (CC) separated the crude extract into fractions A–F. MPLC (MeOH–H_2_O, 5:1, 20:1, 30:1, 45:1, 65:1, 75:1, 85:1, 100:1; 4 L for each step) was used to separate Fraction B into twelve subfractions (B1–B12). Subfraction B6 was subjected to silica gel CC (*v*/*v* 1:0–0:1) to yield subfractions (B6A–B6P). HPLC purified subfraction B6L (MeCN–H_2_O, 34% isocratic, 4 mL/min) to give compound **4** (t_R_ = 9.93 min, 9.8 mg), **5** (t_R_ = 9.92 min, 1.8 mg), **6** (t_R_ = 10.13 min, 9.0 mg), and **7** (t_R_ = 10.61 min, 2.6 mg). Fraction C was separated by MPLC (MeOH–H_2_O, 5:1, 20:1, 30:1, 45:1, 65:1, 75:1, 85:1, 100:1; 4 L for each step) into twenty subfractions (C1–C20). Subfraction C6 was further separated by silica gel CC (CHCl_3_–MeOH from *v*/*v* 1:0 to 0:1) to yield subfractions C6A–C6J. HPLC (MeCN–H_2_O, 32% isocratic, 4 mL/min) purified subfraction C6D to give **8** (t_R_ = 9.90 min, 1.1 mg). Subfraction C10 was separated using silica gel CC (*v*/*v* 1:0–0:1) to obtain subfractions C10A–C10K. HPLC purified subfraction C10A (MeCN–H_2_O, 20% isocratic, 4 mL/min), yielding compound **3** (t_R_ = 9.84 min, 28.3 mg). To obtain subfractions C11A–C11O, silica gel CC (*v*/*v* 1:0–0:1) was used to separate subfraction C11. Subfraction C11L was purified using Sephadex LH-20 (MeOH) and HPLC (MeCN–H_2_O, 25% isocratic, 4 mL/min) to obtain compound **9** (t_R_ = 8.69 min, 9.1 mg). Subfraction C12 was separated by silica gel CC (*v*/*v* 1:0–0:1) to obtain subfractions C12A–C12I. Fraction F was separated into twenty-three subfractions (F1–F23) using MPLC (MeOH–H_2_O, 5:1, 20:1, 30:1, 45:1, 65:1, 75:1, 85:1, 100:1; 4 L for each step). Subfraction F8 was subjected to silica gel CC (*v*/*v* 1:0–0:1) to obtain subfractions F6A–F6H. HPLC (MeCN–H_2_O, 13% isocratic, 4 mL/min) purified subfraction F8B to give compound **10** (t_R_ = 6.43 min, 1.1 mg) and **11** (t_R_ = 6.21 min, 1.0 mg). Subfraction F10 was separated using silica gel CC (*v*/*v* 1:0–0:1) to obtain subfractions F10A–F10L. HPLC purified subfraction F10E (MeCN–H_2_O, 10% isocratic, 4 mL/min), yielding compound **2** (t_R_ = 5.45 min, 16.2 mg). Compound **1** (t_R_ = 7.58 min, 17.3 mg) was obtained by purifying subfraction F10G via HPLC (MeCN–H_2_O, 20% isocratic, 4 mL/min).

### 3.4. Characterization Data

#### 3.4.1. Erinacenone A (**1**)

Yellow oil; UV (MeOH) *λ*_max_ (log *ε*) 225 (4.44), 260 (4.25), 300 (3.63) nm; ^1^H (600 MHz) and ^13^C NMR (150 MHz) data (CD_3_OD), see Table 1; HRESIMS *m/z* 412.13668 (calcd for C_20_H_23_NNaO_7_ [M + Na]^+^, 412.13667).

#### 3.4.2. Erinacenone B (**2**)

Yellow oil; UV (MeOH) *λ*_max_ (log *ε*) 225 (4.49), 260 (4.32), 300 (3.72) nm; ^1^H (500 MHz) and ^13^C NMR (125 MHz) data (CD_3_OD), see Table 1; HRESIMS *m/z* 404.17023 (calcd for C_21_H_26_O_7_ [M + H]^+^, 404.17038).

#### 3.4.3. Erinacenone C (**3**)

Yellow oil; UV (MeOH) *λ*_max_ (log *ε*) 220 (4.72), 265 (4.53), 300 (3.95) nm; ^1^H (600 MHz) and ^13^C NMR (150 MHz) data (CD_3_OD), see Table 2 and Table 3; HRESIMS *m/z* 378.15475 (calcd for C_19_H_24_NO_7_ [M + H]^+^, 378.15473).

#### 3.4.4. Erinacenone D (**4**)

Yellow oil; UV (MeOH) *λ*_max_ (log *ε*) 220 (4.33), 260 (4.12), 300 (3.56) nm; ^1^H (600 MHz) and ^13^C NMR (150 MHz) data (CD_3_OD), see Table 2 and Table 3; HRESIMS *m/z* 378.15463 (calcd for C_19_H_24_NO_7_ [M + H]^+^, 378.15473).

#### 3.4.5. Erinacenone E (**5**)

Yellow oil; UV (MeOH) *λ*_max_ (log *ε*) 215 (4.50), 260 (4.10), 300 (3.53) nm; ^1^H (600 MHz) and ^13^C NMR (150 MHz) data (CD_3_OD), see Table 2 and Table 3; HRESIMS *m/z* 392.17017 (calcd for C_20_H_26_NO_7_ [M + H]^+^, 392.17038).

#### 3.4.6. Erinacenone F (**6**)

Yellow oil; UV (MeOH) *λ*_max_ (log *ε*) 220 (4.48), 260 (4.34), 300 (3.75) nm; ^1^H (600 MHz) and ^13^C NMR (150 MHz) data (CD_3_OD), see Table 2 and Table 3; HRESIMS *m/z* 406.18594 (calcd for C_21_H_28_NO_7_ [M + H]^+^, 406.18603).

#### 3.4.7. Erinacenone G (**7**)

Yellow oil; UV (MeOH) *λ*_max_ (log *ε*) 220 (4.57), 260 (4.40), 300 (3.77) nm; ^1^H (500 MHz) and ^13^C NMR (125 MHz) data (CD_3_OD), see Table 2 and Table 3; HRESIMS *m/z* 442.18347 (calcd for C_22_H_29_NNaO_7_ [M + Na]^+^, 442.18362).

#### 3.4.8. Erinacenone H (**8**)

Yellow oil; [α]^25^_D_+ 28.8 (*c* 0.10, MeOH); UV (MeOH) *λ*_max_ (log *ε*) 210 (4.46), 265 (3.90), 305 (3.33) nm; ^1^H (600 MHz) and ^13^C NMR (150 MHz) data (CD_3_OD), see Table 4 and Table 5; HRESIMS *m/z* 406.18585 (calcd for C_21_H_28_NO_7_ [M + H]^+^, 406.18603).

#### 3.4.9. Erinacenone I (**9**)

Yellow oil; [α]^25^_D_− 10.6 (*c* 0.25, MeOH); UV (MeOH) *λ*_max_ (log *ε*) 220 (4.52), 260 (4.27), 300 (3.68) nm; ^1^H (600 MHz) and ^13^C NMR (150 MHz) data (CD_3_OD), see Table 4 and Table 5; HRESIMS *m/z* 392.17020 (calcd for C_20_H_26_NO_7_ [M + H]^+^, 392.17038).

#### 3.4.10. Erinacenone J (**10**)

Yellow oil; UV (MeOH) *λ*_max_ (log *ε*) 215 (4.42), 260 (4.10), 300 (3.51) nm; ^1^H (600 MHz) and ^13^C NMR (150 MHz) data (CD_3_OD), see Table 4 and Table 5; HRESIMS *m/z* 364.13898 (calcd for C_18_H_22_ N O_7_ [M + H]^+^, 364.13908).

#### 3.4.11. Erinacenone K (**11**)

Yellow oil; UV (MeOH) *λ*_max_ (log *ε*) 215 (4.32), 260 (3.85), 300 (3.34) nm; ^1^H (600 MHz) and ^13^C NMR (150 MHz) data (CD_3_OD), see Table 4 and Table 5; HRESIMS *m/z* 386.12082 (calcd for C_18_H_21_NNaO_7_ [M + Na]^+^, 386.12102).

#### 3.4.12. Erinacenone L (**12**)

Yellow oil; [α]^25^_D_− 69.0 (*c* 0.05, MeOH); UV (MeOH) *λ*_max_ (log *ε*) 225 (4.62), 265 (4.46) nm; ^1^H (500 MHz) and ^13^C NMR (125 MHz) data (CD_3_OD), see Table 4 and Table 5; HRESIMS *m/z* 456.16529 (calcd for C_24_H_26_NO_8_ [M + H]^+^, 456.16529).

### 3.5. Cytotoxicity Assay

Compounds **1**–**12** were assessed for cytotoxicity against MCF-7 cell lines (ATCC, Manassas, VA, USA) using the MTT (multiple table tournament) method, as previously reported. Briefly, 1 × 10^5^ cells/mL of adherent cells were seeded in 96-well plates and incubated for 12 h at 37 °C. After this initial period, various concentrations of compounds were added to each well. Following a 48 h incubation, MTT solution was added and incubated for an additional 4 h. Then, MTT was removed and dissolved with MTT lysis solution (20% SDS, 50% DMF). MCF-7 cells were cultured in DMEM medium (Hyclone, Logan, UT, USA) with 10% fetal bovine serum (FBS) supplemented at 37 °C in 5% CO_2_. Cisplatin served as the positive control. The absorbances were detected at 595 nm on an Envision multilabel plate reader, and the IC_50_ values were calculated using the Reed-Muench method [18].

## 4. Conclusions

In the present study, a total of twelve previously unreported isoindolin-1-one compounds, erinacenone A–L (**1**–**12**), were isolated from liquid cultures of the medicinal fungus *H. erinaceus*. Their structures were elucidated based on spectroscopic data analysis. The absolute configuration of **12** was determined by comparing its optical rotations with values reported in the literature. The most distinctive feature of these compounds is that their nitrogen atoms are connected to different parts of the special structure moieties. Among them, compounds **3** and **4**, as well as **10** and **11**, are two pairs of isomers differing only by a small change in the position of one double bond. The structures of the twelve isolated compounds are very similar to those reported in the research of Wang et al. [17], Lin et al. [19], and Chen et al., [20], sharing the same core structure. Compounds **4** and **5** were found to show cytotoxic activities with IC_50_ values of 24.7 and 18.4 μM, respectively, against MCF-7 cell lines (Table 6).

So far, despite the fact that the chemical constituents of the fruiting body and fermentation broth of the mushroom *H. erinaceus* have been well studied, many new secondary metabolites can still be isolated and obtained. This suggests that strains collected in different regions and seasons can produce a rich variety of secondary metabolites with structural changes under changing fermentation conditions. The appearance of these new secondary metabolites opens up the possibility of discovering new biological activities of these components. It also shows that the mushroom *H. erinaceus* itself is a treasure trove with great potential that we need to keep exploring. The new alkaloids discovered in the present study have only been screened using simple bioassays so far, and the next step needs to be a systematic screening of different drug targets, with special attention given to their testing on central nervous system models. In addition, the continued search for other types of secondary metabolites from the mushroom *H. erinaceus* should also be considered.

## Figures and Tables

**Table 1:** ^1^H NMR (600 MHz) and ^13^C NMR (150 MHz) spectroscopic data for compounds **1**–**2**.

No.	1		2	
1	171.8		171.9	
3	50.0	4.39, s	44.7	4.39, s
3a	131.3		131.1	
4	151.6		151.7	
5	121.7		121.9	
6	158.0		158.1	
7	102.0	6.76, s	102.0	6.76, s
7a	121.0		121.0	
1′	23.6	3.42, dd (7.1)	23.6	3.43, dd (7.1)
2′	124.5	5.30, t (7.1)	124.3	5.30, t (7.1)
3′	134.8		135.0	
4′	39.4	2.09, t (7.3)	39.5	2.09, t (7.4)
5′	28.3	2.28, dd (14.9, 7.3)	28.4	2.28, dd (14.9, 7.4)
6′	143.8	6.72, t (7.3)	143.3	6.71, t (7.4)
7′	128.8		129.2	
8′	171.7		172.4	
9′	16.3	1.82, s	16.3	1.82, s
10′	12.4	1.76, s	12.5	1.76, s
1″	45.0	4.32, s	50.0	4.39, overlapped
2″	173.0		171.2	
3″			52.8	3.76, s

Measured in methanol-*d*_4._

**Table 2:** ^1^H NMR (600 MHz) spectroscopic data for compounds **3**–**7**.

No.	3	4	5	6	7
3	4.38, s	4.39, s	4.34, s	4.30, s	4.30, s
7	6.76, s	6.76, s	6.72, s	6.73, s	6.73, s
1′	3.40, d (7.1)	2.83, m	3.39, d (7.1)	3.40, d (7.1)	3.40, d (7.1)
2′	5.28, t (7.1)	2.24, t (7.8)	5.27, t (7.1)	5.27, t (7.1)	5.28, t (7.1)
4′	2.26, t (7.6)	5.26, t (7.1)	2.25, t (7.6)	2.25, t (7.6)	2.25, t (7.5)
5′	2.39, m	3.04, d (7.1)	2.39, t (7.6)	2.39, overlapped	2.39, overlapped
7′	1.80, s	1.74, s	1.79, s	1.79, s	1.80, s
8′	3.55, s	3.64, s	3.55, s	3.55, s	3.55, s
1″	4.38, s	4.39, s	3.85, t (6.7)	3.62, t (6.8)	3.60, s
2″			2.73, t (6.7)	1.98, m	1.71, m
3″		3.76, s		2.37, overlapped	1.63, m
4″	3.75, s		3.67, s		2.40, overlapped
5″				3.58, s	
6″					3.64, s

Measured in methanol-*d*_4._

**Table 3:** ^13^C NMR (150 MHz) spectroscopic data for compounds **3**–**7**.

No.	3	4	5	6	7
1	171.8	171.1	171.4	171.5	171.4
3	49.9	44.7	49.7	48.8	48.8
3a	131.1	131.1	131.7	131.8	132.0
4	151.7	151.8	151.6	151.6	151.6
5	121.8	122.4	121.4	121.3	121.3
6	158.1	158.3	158.1	158.1	158.1
7	102.0	101.9	101.7	101.8	101.8
7a	121.0	121.0	120.6	120.4	120.4
1′	23.6	23.4	23.6	23.6	23.6
2′	124.5	39.5	124.6	124.6	124.6
3′	134.3	140.7	134.2	134.2	134.2
4′	35.9	116.8	36.0	36.0	36.0
5′	33.9	34.3	33.9	33.9	33.9
6′	175.8	174.8	175.8	175.8	175.8
7′	16.1	16.4	16.1	16.1	16.1
8′	51.9	52.3	51.9	51.9	51.9
1″	44.7	50.0	39.8	42.9	43.0
2″	171.1	171.8	33.9	24.7	28.7
3″	52.8	52.8	173.7	32.0	23.1
4″			52.3	175.2	34.1
5″				52.1	175.6
6″					52.0

Measured in methanol-*d*_4._

**Table 4:** ^1^H NMR (600 MHz) spectroscopic data for compounds **8**–**12**.

No.	8	9	10	11	12
3	4.60, d (17.0); 4.32, d (17.0)	4.59, overlapped; 4.32, d (17.0)	4.38, s	4.39, s	4.37, d (16.6); 4.27, d (16.6)
7	6.75, s	6.75, s	6.76, s	6.75, s	6.67, s
1′	3.41, d (7.1)	3.42, d (7.2)	3.42, d (7.1)	2.83, t (7.6)	3.39, m
2′	5.28, t (7.1)	5.31, t (7.2)	5.30, t (7.1)	2.21, t (7.6)	5.29, t (7.4)
4′	2.26, t (7.6)	2.26, t (7.2)	2.26, m	5.39, m	2.25, overlapped
5′	2.39, m	2.36, overlapped	2.32, m	2.97, m	2.35, m
7′	1.80, d	1.81, s	1.81, s	1.74, s	1.80, s
8′	3.55, s				
1″	4.56, m	4.58, overlapped	4.39, s	4.39, s	5.10, dd (11.2, 4.7)
2″	2.34, m	2.35, overlapped			3.11, dd (14.6, 11.4); 2.25, overlapped
3″	1.09, d (6.6)	1.09, d (6.6)	3.78, s	3.76, s	
4″	0.89, d (6.6)	0.9, d (6.6)			7.04, d (8.5)
5″					6.65, d (8.5)
7″					6.65, d (8.5)
8″					7.04, d (8.5)

Measured in methanol-*d*_4._

**Table 5:** ^13^C NMR (150 MHz) spectroscopic data for compounds **8**–**12**.

No.	8	9	10	11	12
1	171.8	171.9	171.8	171.9	171.8
3	46.8	46.8	50.0	44.7	47.0
3a	131.2	131.1	131.1	131.0	131.4
4	151.6	151.6	151.7	151.8	151.5
5	121.6	121.7	121.9	122.8	121.5
6	158.1	158.1	158.2	158.3	158.0
7	101.9	102.0	102.0	6.75	101.9
7a	120.9	120.9	121.0	121.2	120.9
1′	23.6	23.6	23.6	23.6	23.6
2′	124.6	124.2	123.9	39.8	124.2
3′	134.2	134.5	135.1	138.6	134.5
4′	36.0	36.0	36.6	119.5	35.9
5′	33.9	34.1	35.4	37.1	34.0
6′	175.8	177.6	179.6	nd ^a^	177.6
7′	16.1	16.2	16.3	16.4	16.2
8′	51.9				
1″	62.8	62.5	44.7	50.0	57.5
2″	30.1	30.1	171.1	171.2	35.9
3″	20.1	20.0	52.8	52.8	129.3
4″	19.6	19.2			130.6
5″	nd ^a^	174.5			116.4
6″					157.2
7″					116.4
8″					130.6
9″					174.5

^a^ Not detected. Measured in methanol-*d*_4_.

**Table 6:** Cytotoxicity inhibitory activity of compounds (IC_50_, µM).

Compounds	MCF-7
**4**	24.7
**5**	18.4
Cisplatin ^a^	9.12

^a^ Positive control. Other compounds did not show inhibitory activity, or very weak activity. The maximum tested concentration is 100 µM.

## Data Availability

No new data were created or analyzed in this study. Data sharing is not applicable to this article.

## Supplementary Materials

The following supporting information can be downloaded at: https://www.mdpi.com/article/10.3390/molecules29204901/s1, Figures S1–S84: HRESIMS, HNMR, CNMR, COSY, HSQC, HMBC, ROESY of compounds **1**–**12**.
