# Supplementary material for: Erinacenones A–L: Twelve New Isoindolinone Alkaloids from the Edible and Medicinal Mushroom Hericium erinaceus
Source: Molecules. 2024 Oct 16;29(20):4901. doi: 10.3390/molecules29204901 (PMC11510660; doi:10.3390/molecules29204901)

SUPPLEMENTARY MATERIAL

Erinacenones A–L: Twelve New Isoindolinone Alkaloids from the Edible and Medicinal Mushroom *Hericium erinaceus*

Lin-Lin Yuan and Ji-Kai Liu *

Anhui Province Key Laboratory of Bioactive Natural Products, School of Pharmaceutical Sciences, Anhui University of Chinese Medicine, Hefei 230012, China

Emails: liujikai@mail.scuec.edu.cn (J.-K. Liu)

Contents

Figure S1. HRESIMS spectroscopic data of compound 1. 3
Figure S2. ^{1}H NMR (600 MHz) spectrum of compound 1. 4
Figure S3. ^{13}C NMR and DEPT (150 MHz) spectra of compound 1. 4
Figure S4. ^{1}H-^{1}H COSY spectrum of compound 1. 5
Figure S5. HSQC spectrum of compound 1. 5
Figure S6. HMBC spectrum of compound 1. 6
Figure S7. ROESY spectrum of compound 1. 6
Figure S8. HRESIMS spectroscopic data of compound 2. 7
Figure S9. ^{1}H NMR (500 MHz) spectrum of compound 2. 8
Figure S10. ^{13}C NMR and DEPT (125 MHz) spectra of compound 2. 8
Figure S11. ^{1}H-^{1}H COSY spectrum of compound 2. 9
Figure S12. HSQC spectrum of compound 2. 9
Figure S13. HMBC spectrum of compound 2. 10
Figure S14. ROESY spectrum of compound 2. 10
Figure S15. HRESIMS spectroscopic data of compound 3. 11
Figure S16. ^{1}H NMR (600 MHz) spectrum of compound 3. 11
Figure S17. ^{13}C NMR and DEPT (150 MHz) spectra of compound 3. 12
Figure S18. ^{1}H-^{1}H COSY spectrum of compound 3. 12
Figure S19. HSQC spectrum of compound 3. 13
Figure S20. HMBC spectrum of compound 3. 13
Figure S21. ROESY spectrum of compound 3. 14
Figure S22. HRESIMS spectroscopic data of compound 4. 15
Figure S23. ^{1}H NMR (600 MHz) spectrum of compound 4. 16
Figure S24. ^{13}C NMR and DEPT (150 MHz) spectra of compound 4. 16
Figure S25. ^{1}H-^{1}H COSY spectrum of compound 4. 17
Figure S26. HSQC spectrum of compound 4. 17
Figure S27. HMBC spectrum of compound 4. 18
Figure S28. ROESY spectrum of compound 4. 18
Figure S29. HRESIMS spectroscopic data of compound 5. 19
Figure S30. ^{1}H NMR (600 MHz) spectrum of compound 5. 20
Figure S31. ^{13}C NMR and DEPT (150 MHz) spectra of compound 5. 20
Figure S32. ^{1}H-^{1}H COSY spectrum of compound 5. 21
Figure S33. HSQC spectrum of compound 5. 21
Figure S34. HMBC spectrum of compound 5. 22
Figure S35. ROESY spectrum of compound 5. 22
Figure S36. HRESIMS spectroscopic data of compound 6. 23
Figure S37. ^{1}H NMR (600 MHz) spectrum of compound 6. 24
Figure S38. ^{13}C NMR and DEPT (150 MHz) spectra of compound 6. 24
Figure S39. ^{1}H-^{1}H COSY spectrum of compound 6. 25
Figure S40. HSQC spectrum of compound 6. 25
Figure S41. HMBC spectrum of compound 6. 26

Figure S42. ROESY spectrum of compound 6. 26
Figure S43. HRESIMS spectroscopic data of compound 7. 27
Figure S44. ^{1}H NMR (500 MHz) spectrum of compound 7. 28
Figure S45. ^{13}C NMR and DEPT (125 MHz) spectra of compound 7. 28
Figure S46. ^{1}H-^{1}H COSY spectrum of compound 7. 29
Figure S47. HSQC spectrum of compound 7. 29
Figure S48. HMBC spectrum of compound 7. 30
Figure S49. ROESY spectrum of compound 7. 30
Figure S50. HRESIMS spectroscopic data of compound 8. 31
Figure S51. ^{1}H NMR (600 MHz) spectrum of compound 8. 31
Figure S52. ^{13}C NMR and DEPT (150 MHz) spectra of compound 8. 32
Figure S53. ^{1}H-^{1}H COSY spectrum of compound 8. 32
Figure S54. HSQC spectrum of compound 8. 33
Figure S55. HMBC spectrum of compound 8. 33
Figure S56. ROESY spectrum of compound 8. 34
Figure S57. HRESIMS spectroscopic data of compound 9. 35
Figure S58. ^{1}H NMR (600 MHz) spectrum of compound 9. 36
Figure S59. ^{13}C NMR and DEPT (150 MHz) spectra of compound 9. 36
Figure S60. ^{1}H-^{1}H COSY spectrum of compound 9. 37
Figure S61. HSQC spectrum of compound 9. 37
Figure S62. HMBC spectrum of compound 9. 38
Figure S63. ROESY spectrum of compound 9. 38
Figure S64. HRESIMS spectroscopic data of compound 10. 39
Figure S65. ^{1}H NMR (600 MHz) spectrum of compound 10. 40
Figure S66. ^{13}C NMR and DEPT (150 MHz) spectra of compound 10. 40
Figure S67. ^{1}H-^{1}H COSY spectrum of compound 10. 41
Figure S68. HSQC spectrum of compound 10. 41
Figure S69. HMBC spectrum of compound 10. 42
Figure S70. ROESY spectrum of compound 10. 42
Figure S71. HRESIMS spectroscopic data of compound 11. 43
Figure S72. ^{1}H NMR (600 MHz) spectrum of compound 11. 44
Figure S73. ^{13}C NMR and DEPT (150 MHz) spectra of compound 11. 44
Figure S74. ^{1}H-^{1}H COSY spectrum of compound 11. 45
Figure S75. HSQC spectrum of compound 11. 45
Figure S76. HMBC spectrum of compound 11. 46
Figure S77. ROESY spectrum of compound 11. 46
Figure S78. HRESIMS spectroscopic data of compound 12. 47
Figure S79. ^{1}H NMR (500 MHz) spectrum of compound 12. 48
Figure S80. ^{13}C NMR and DEPT (125 MHz) spectra of compound 12. 48
Figure S81. ^{1}H-^{1}H COSY spectrum of compound 12. 49
Figure S82. HSQC spectrum of compound 12. 49
Figure S83. HMBC spectrum of compound 12. 50
Figure S84. ROESY spectrum of compound 12. 50

Figure S1. HRESIMS spectroscopic data of compound 1.

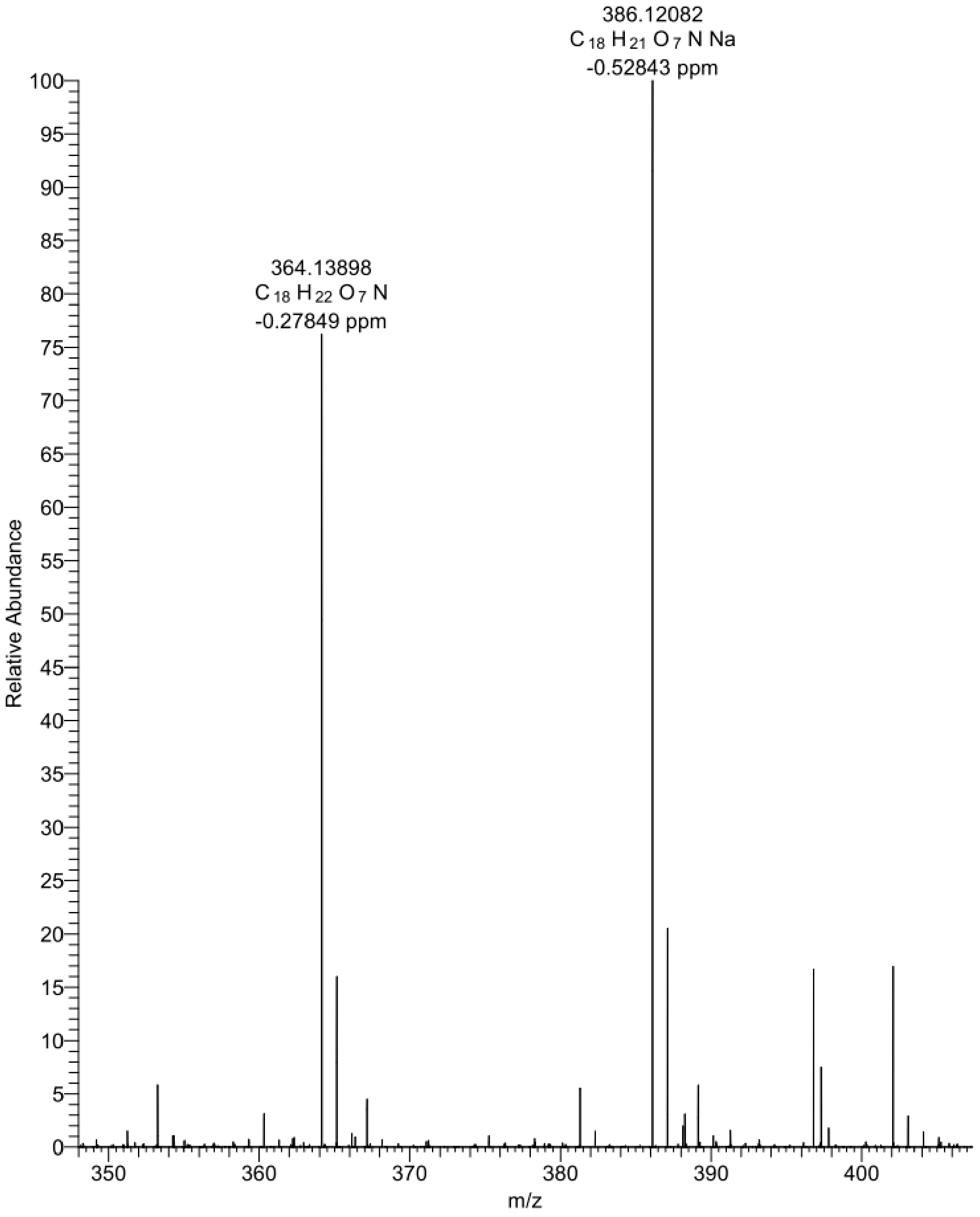

Figure S2. ^{1}H NMR (600 MHz) spectrum of compound 1.

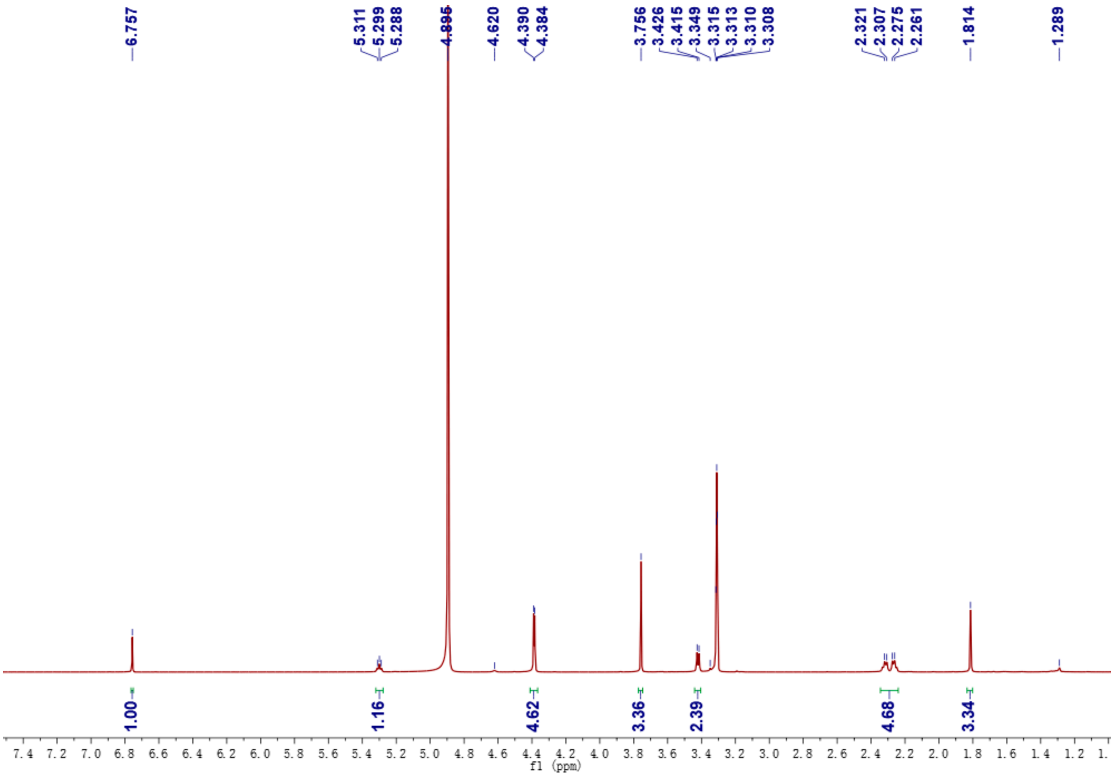

Figure S3. ^{13}C NMR and DEPT (150 MHz) spectra of compound 1.

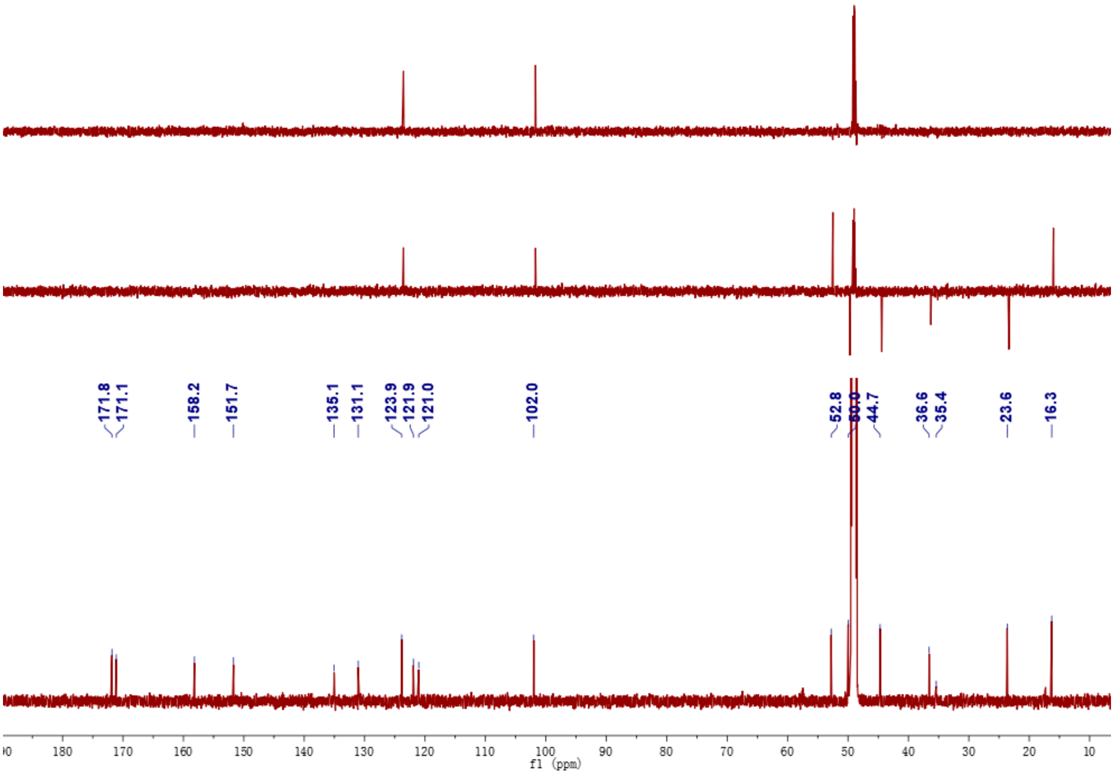

Figure S4. ^{1}H-^{1}H COSY spectrum of compound 1.

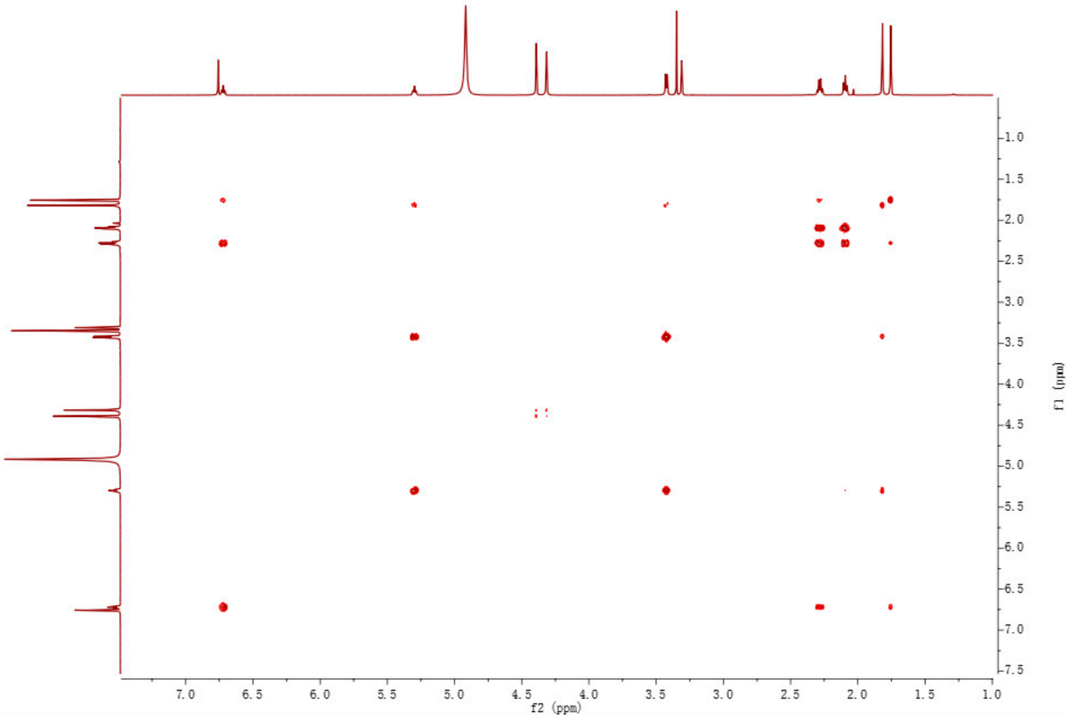

Figure S5. HSQC spectrum of compound 1.

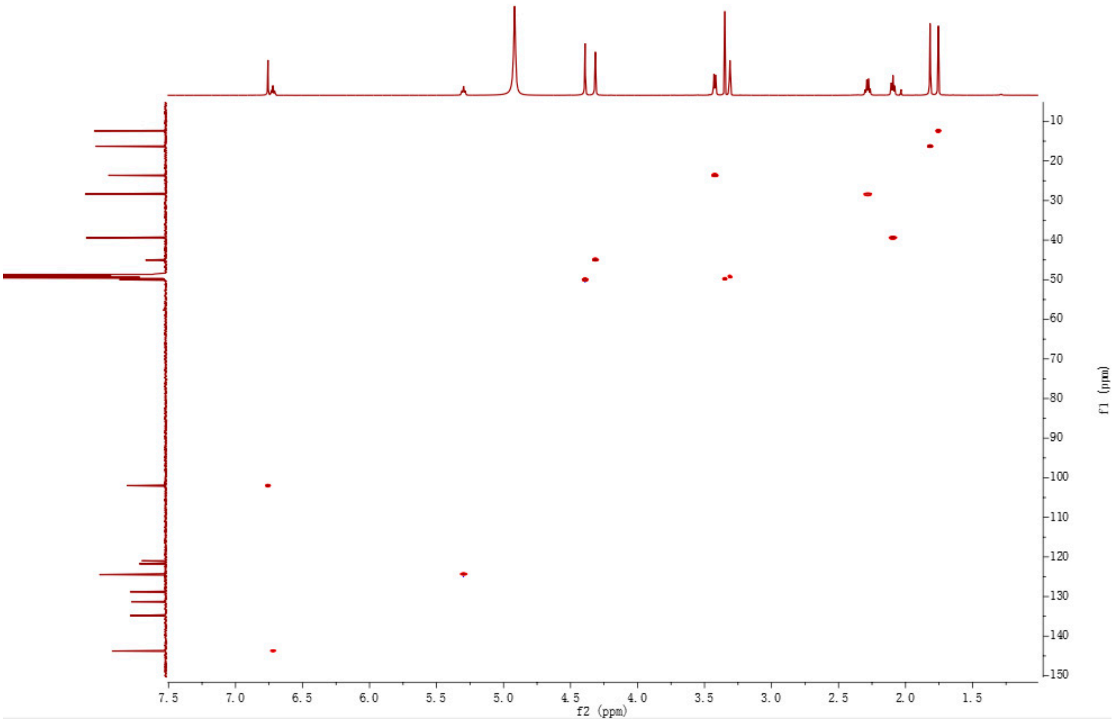

Figure S6. HMBC spectrum of compound 1.

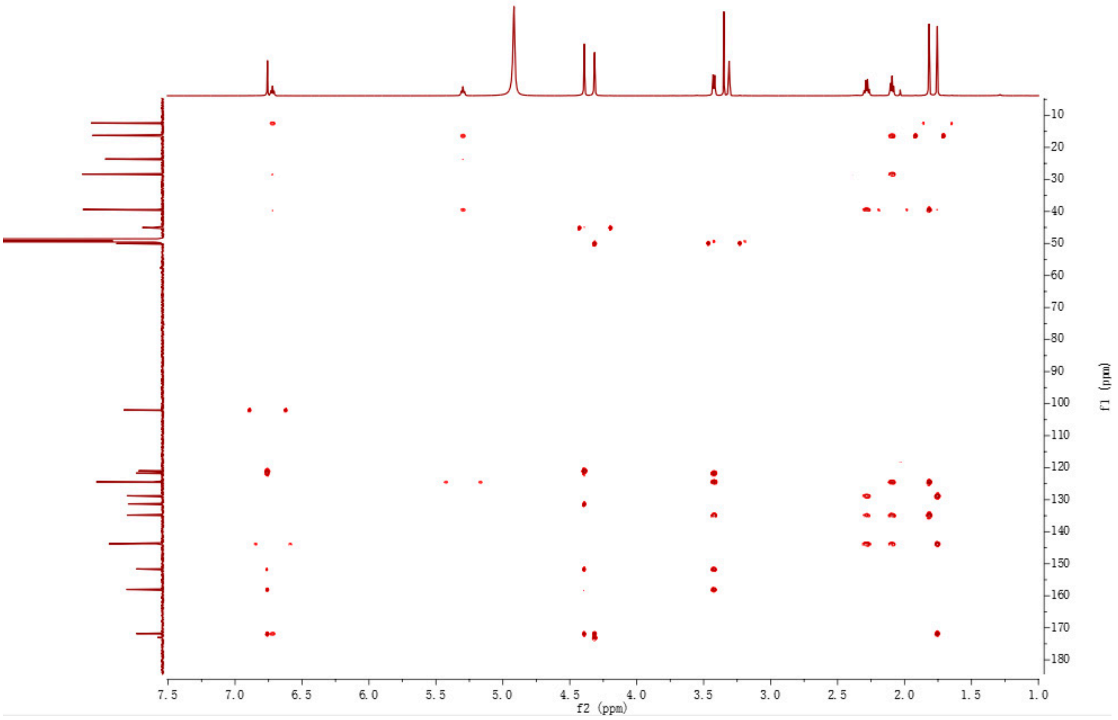

Figure S7. ROESY spectrum of compound 1.

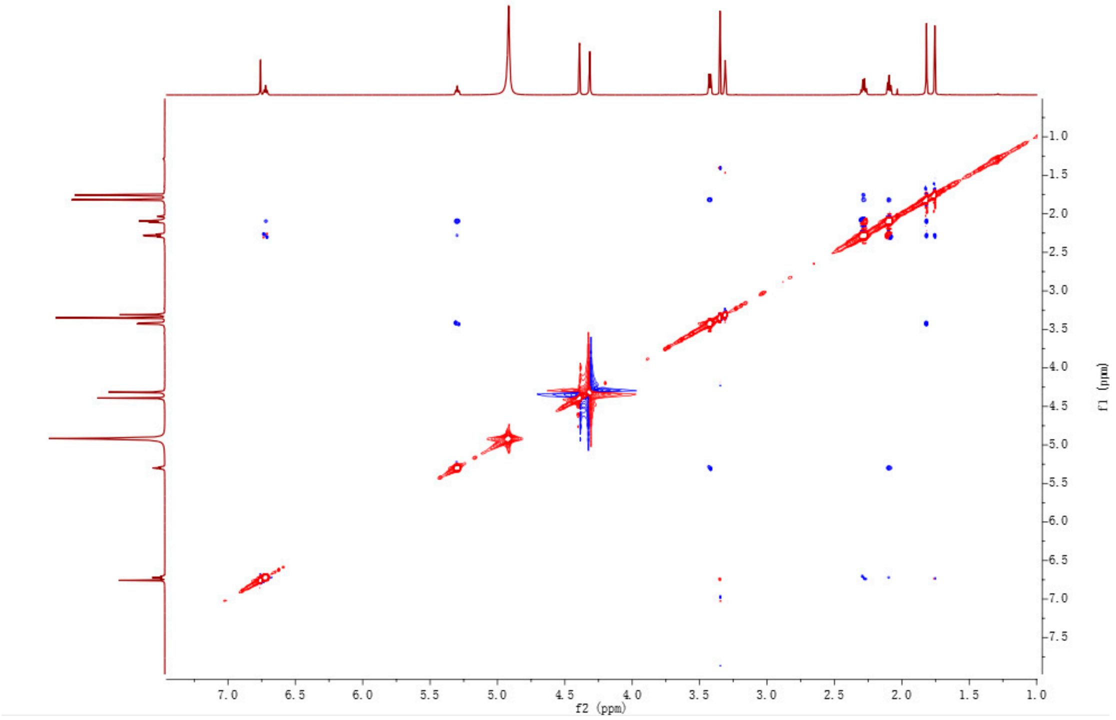

Figure S8. HRESIMS spectroscopic data of compound 2.

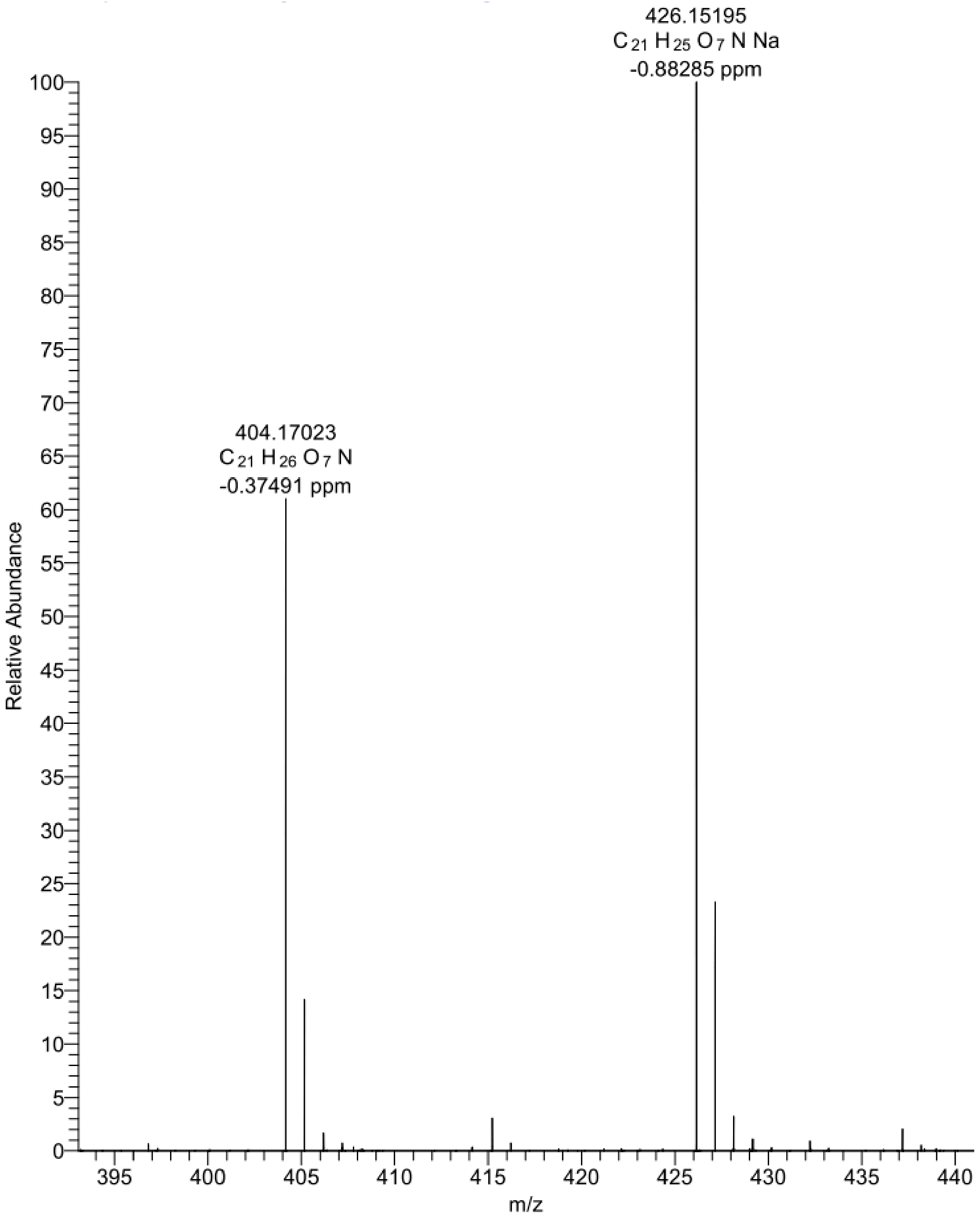

Figure S9. ^{1}H NMR (500 MHz) spectrum of compound 2.

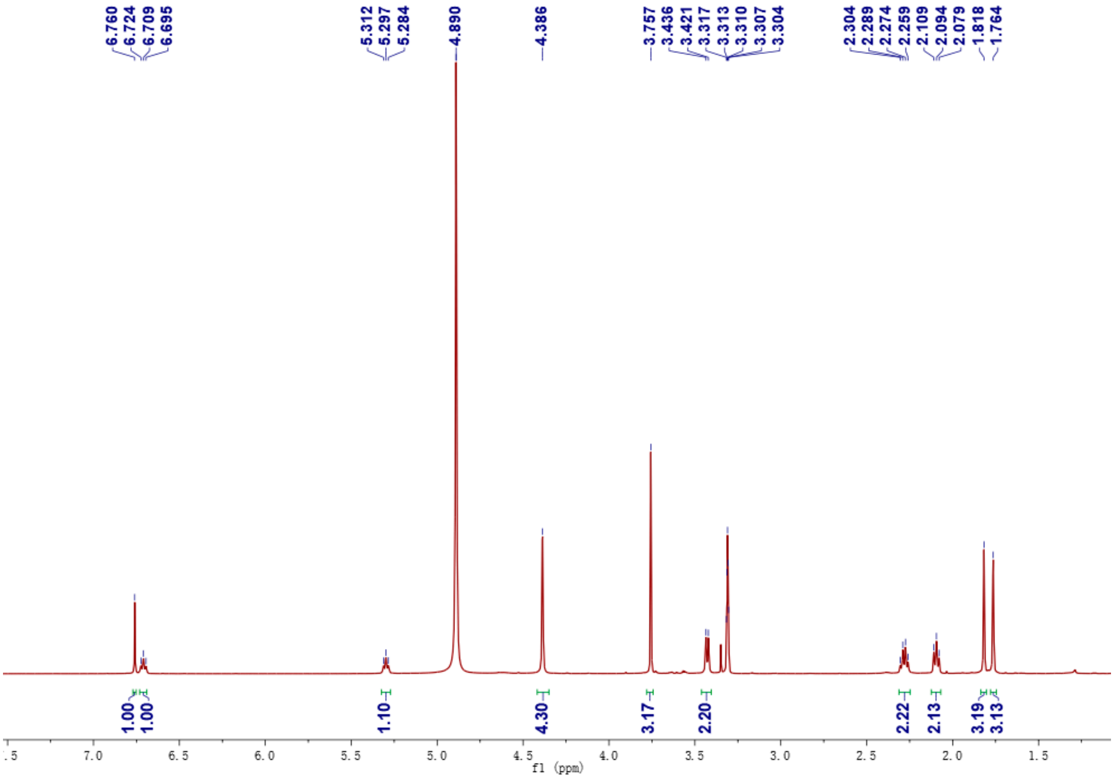

Figure S10. ^{13}C NMR and DEPT (125 MHz) spectra of compound 2.

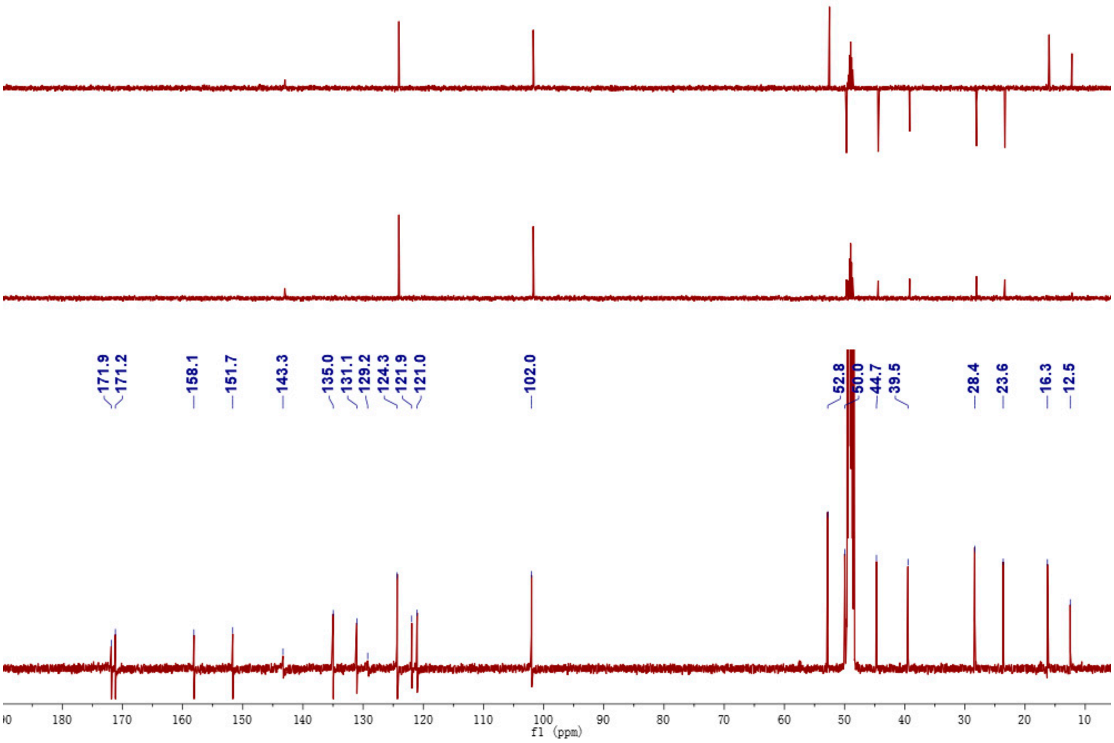

Figure S11. ^{1}H-^{1}H COSY spectrum of compound 2.

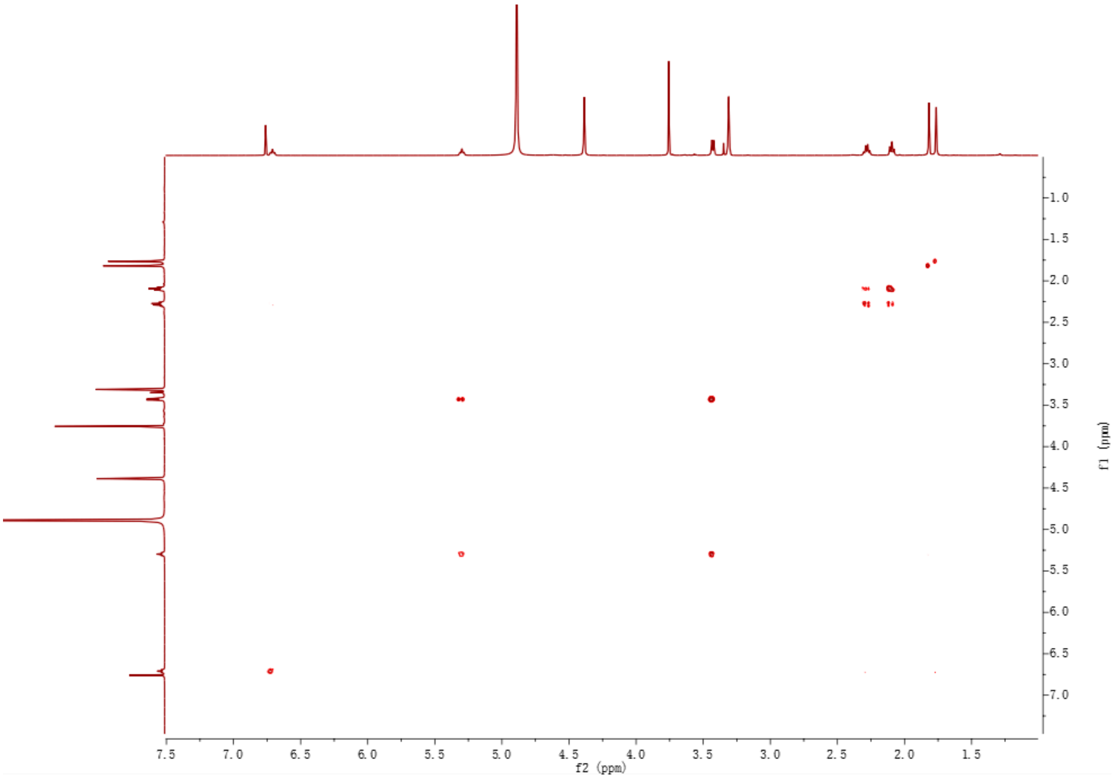

Figure S12. HSQC spectrum of compound 2.

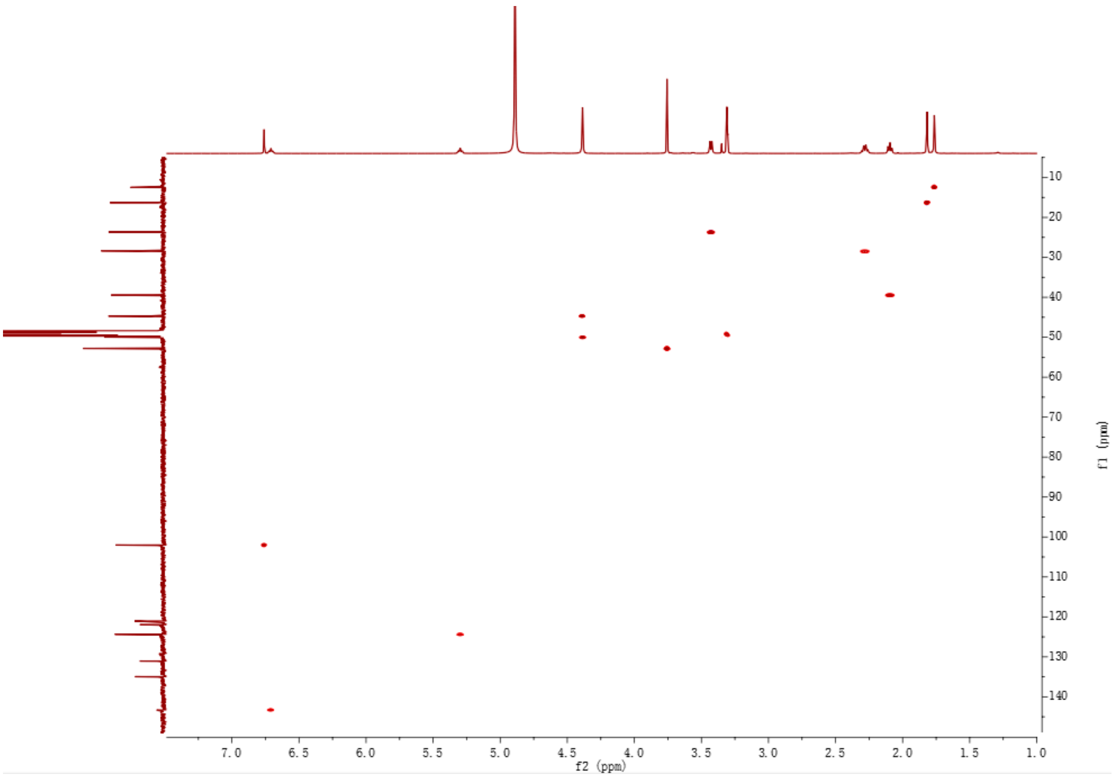

Figure S13. HMBC spectrum of compound 2.

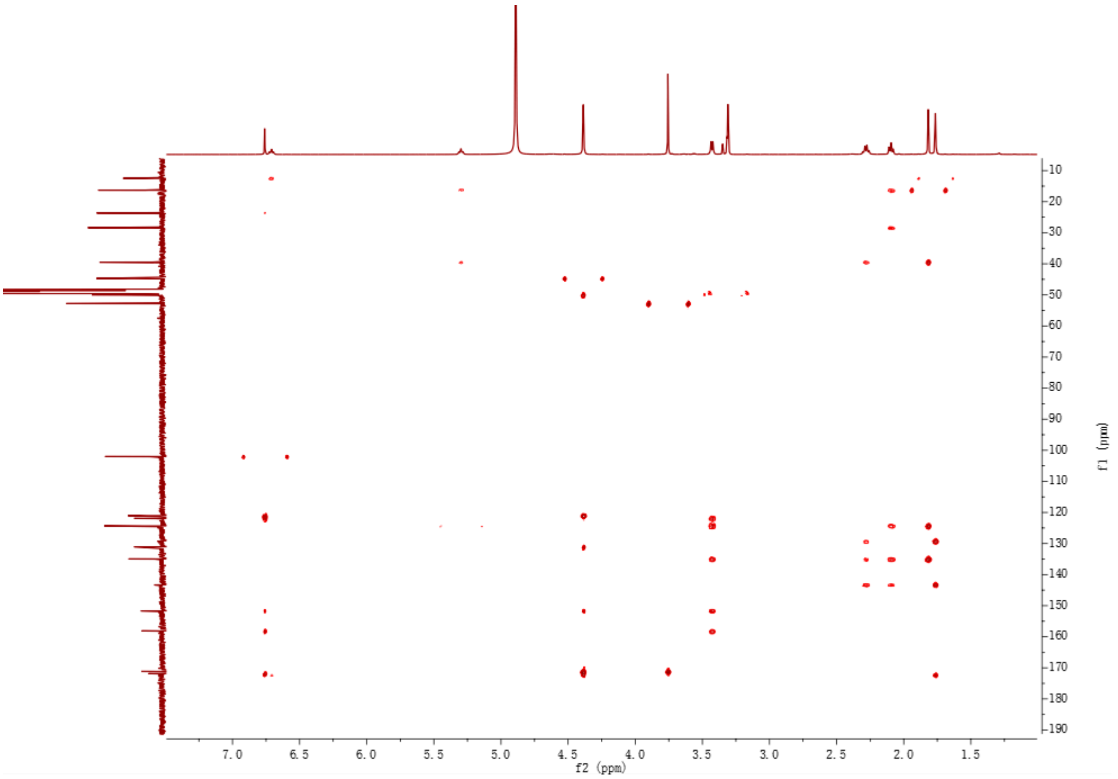

Figure S14. ROESY spectrum of compound 2.

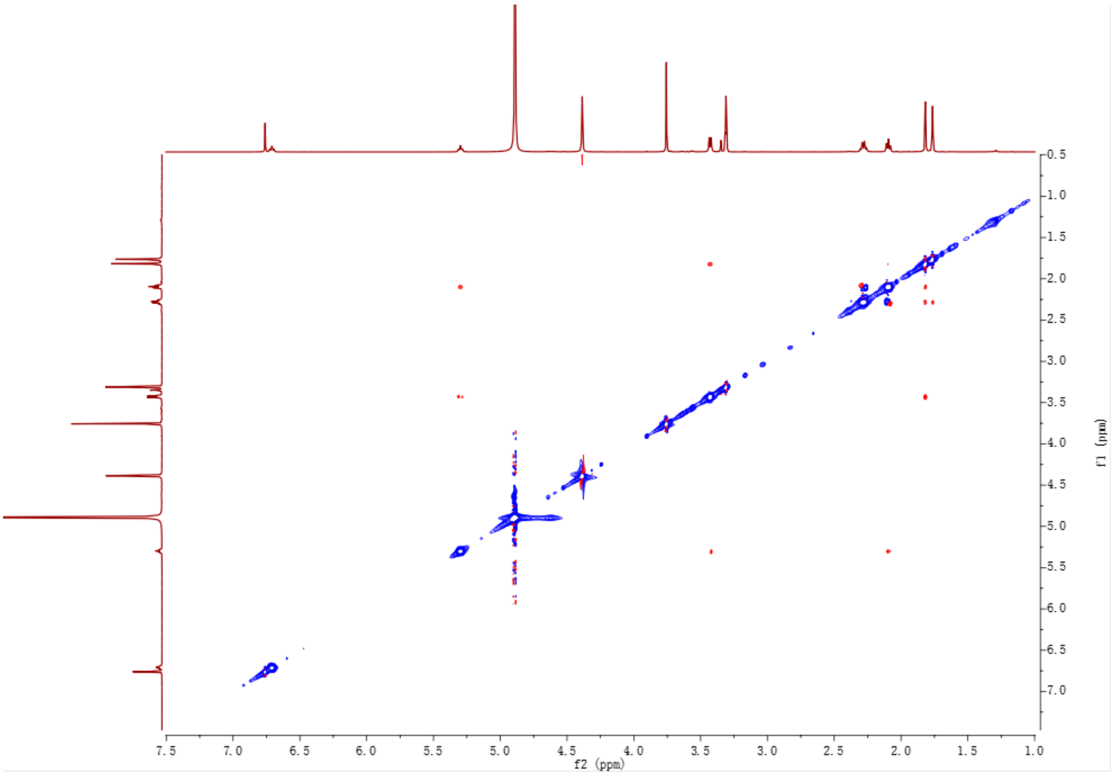

Figure S15. HRESIMS spectroscopic data of compound 3.

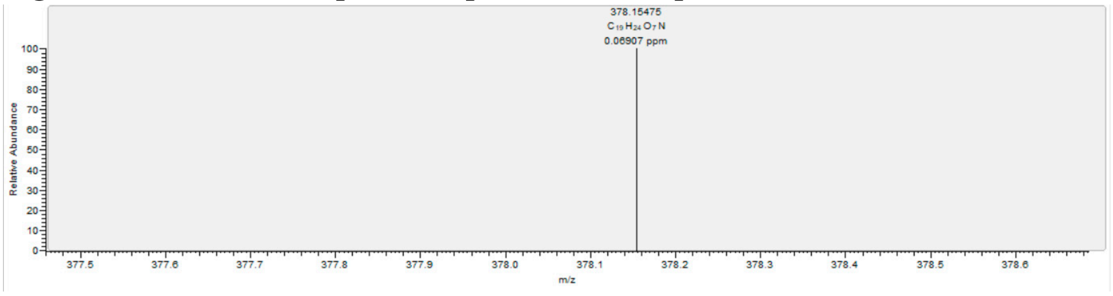

Figure S16. ^{1}H NMR (600 MHz) spectrum of compound 3.

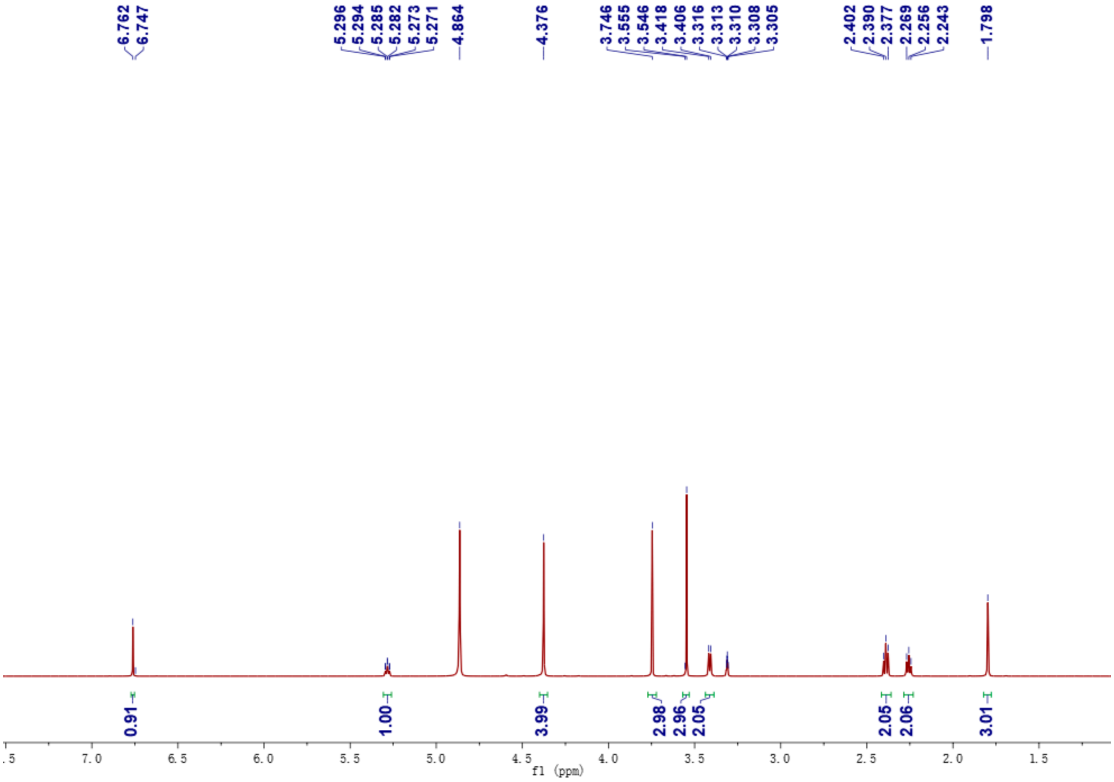

Figure S17. ^{13}C NMR and DEPT (150 MHz) spectra of compound 3.

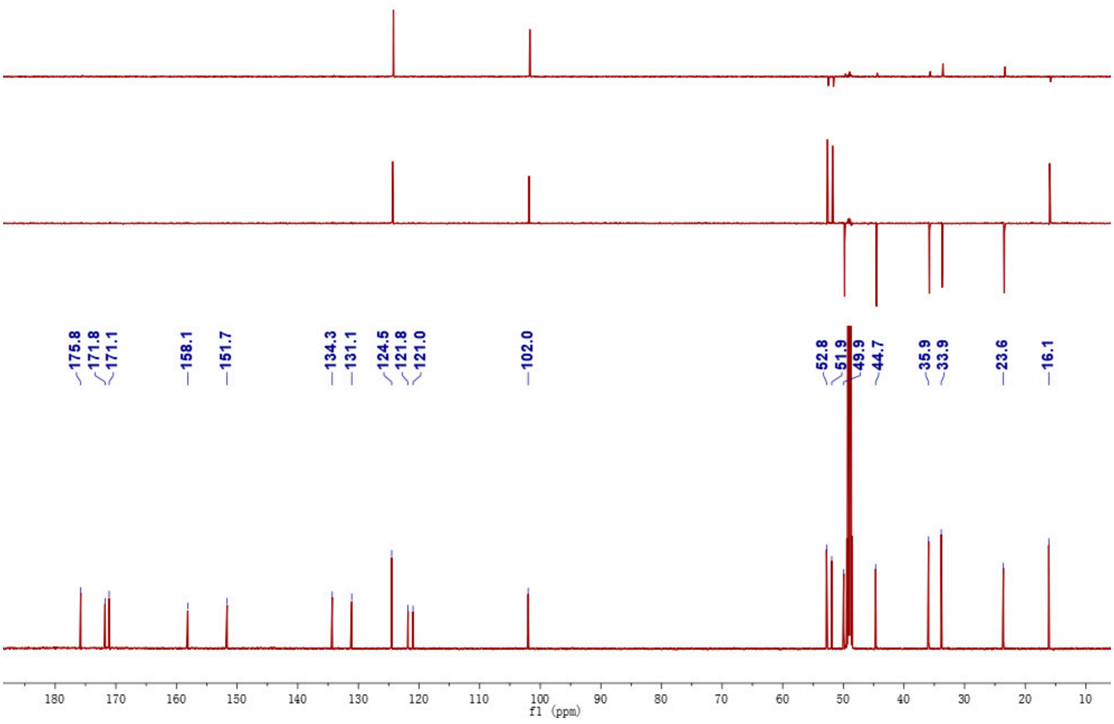

Figure S18. ^{1}H-^{1}H COSY spectrum of compound 3.

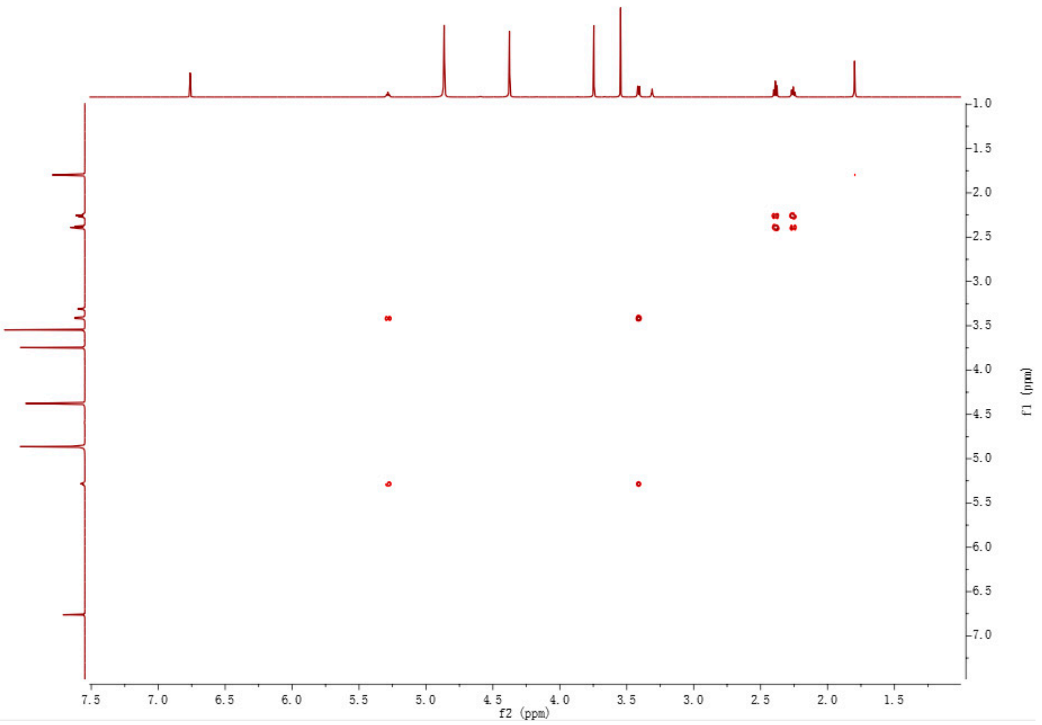

Figure S19. HSQC spectrum of compound 3.

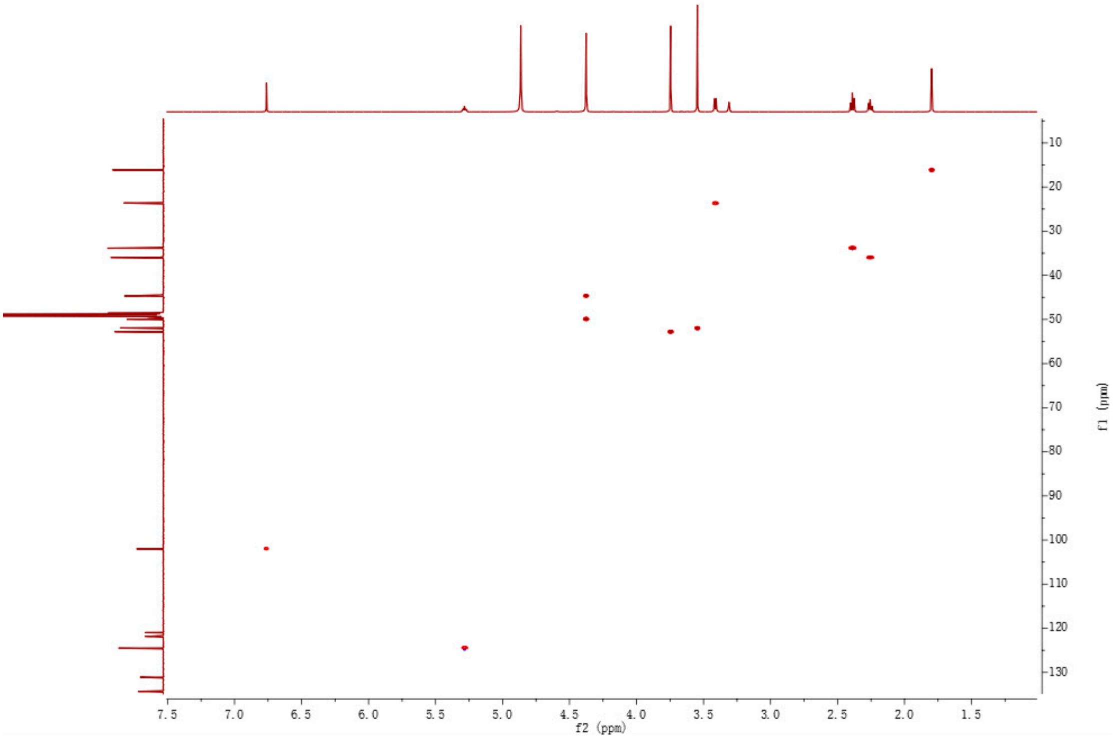

Figure S20. HMBC spectrum of compound 3.

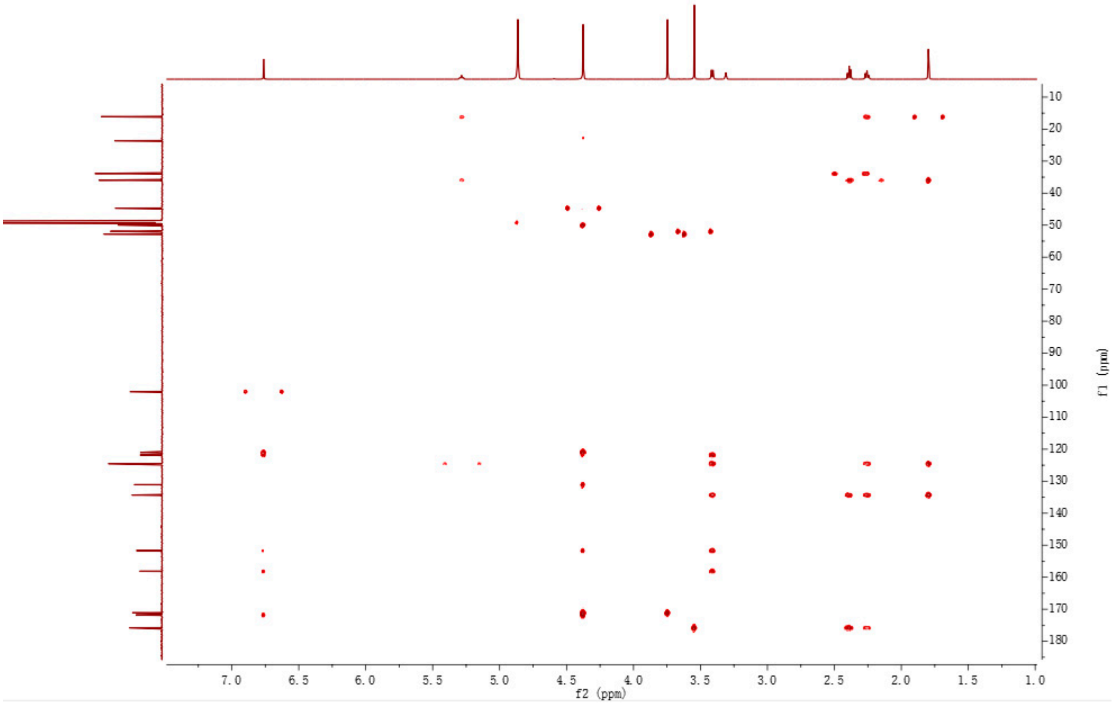

Figure S21. ROESY spectrum of compound 3.

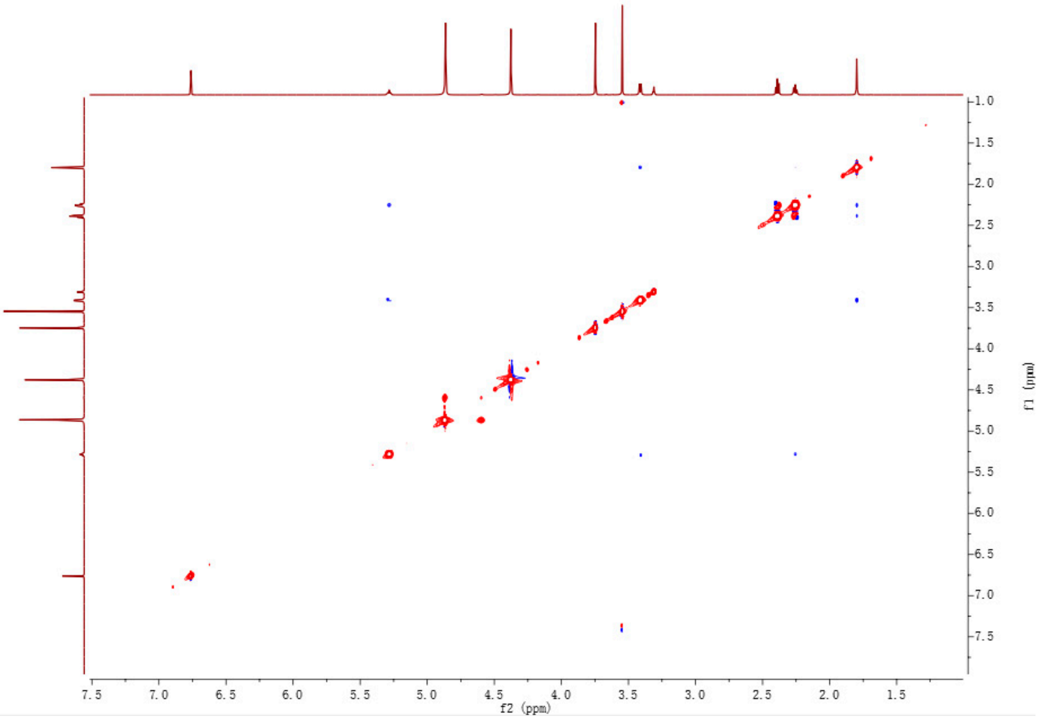

Figure S22. HRESIMS spectroscopic data of compound 4.

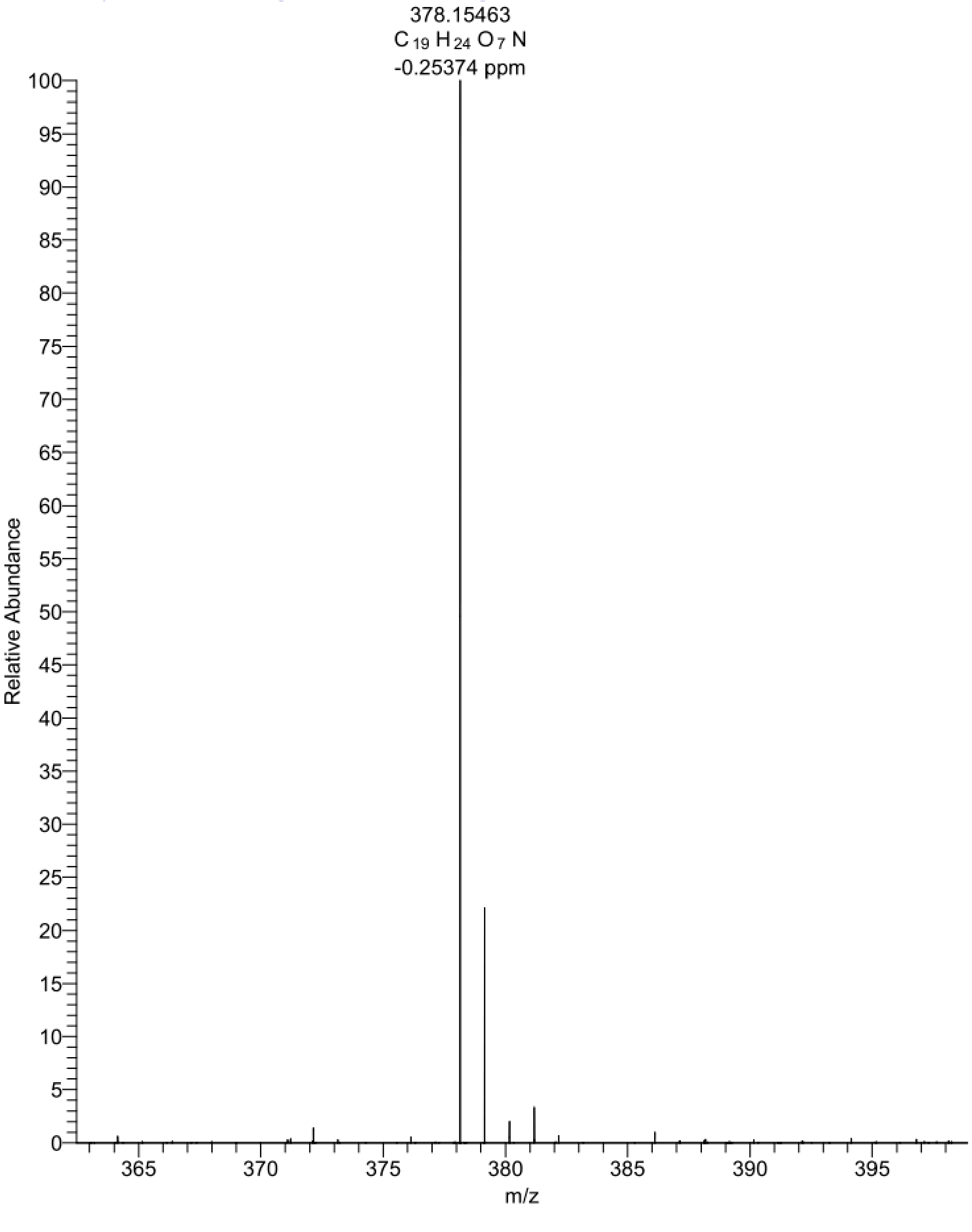

Figure S23. ^{1}H NMR (600 MHz) spectrum of compound 4.

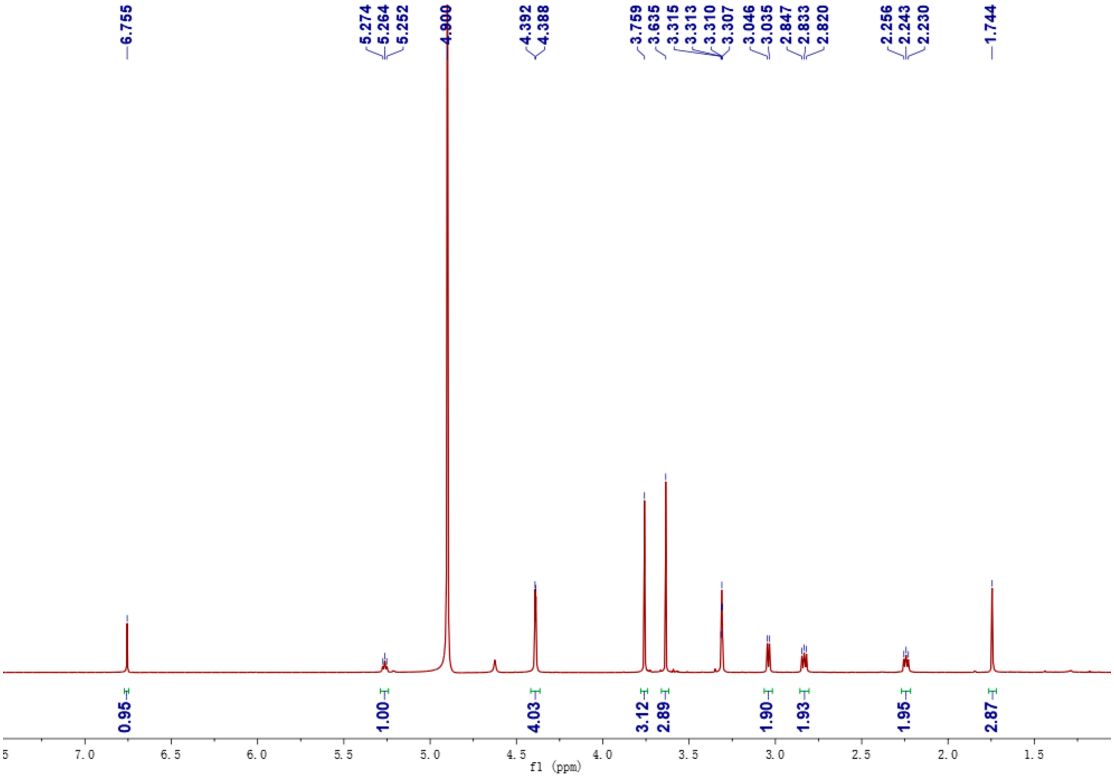

Figure S24. ^{13}C NMR and DEPT (150 MHz) spectra of compound 4.

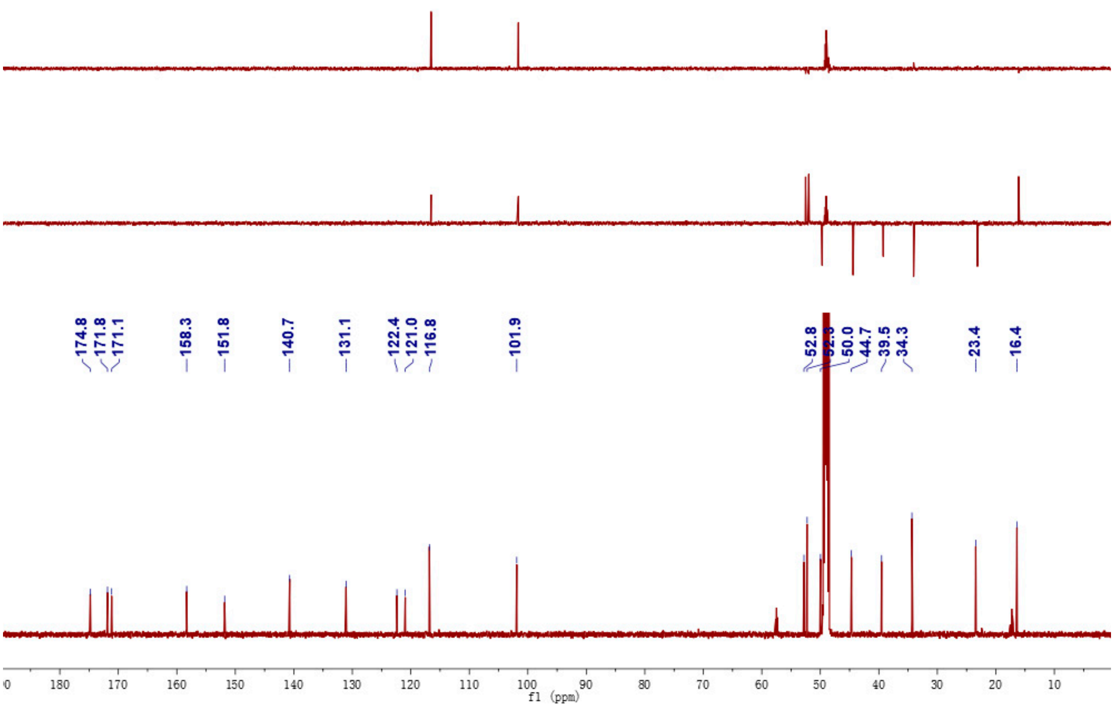

Figure S25. ^{1}H-^{1}H COSY spectrum of compound 4.

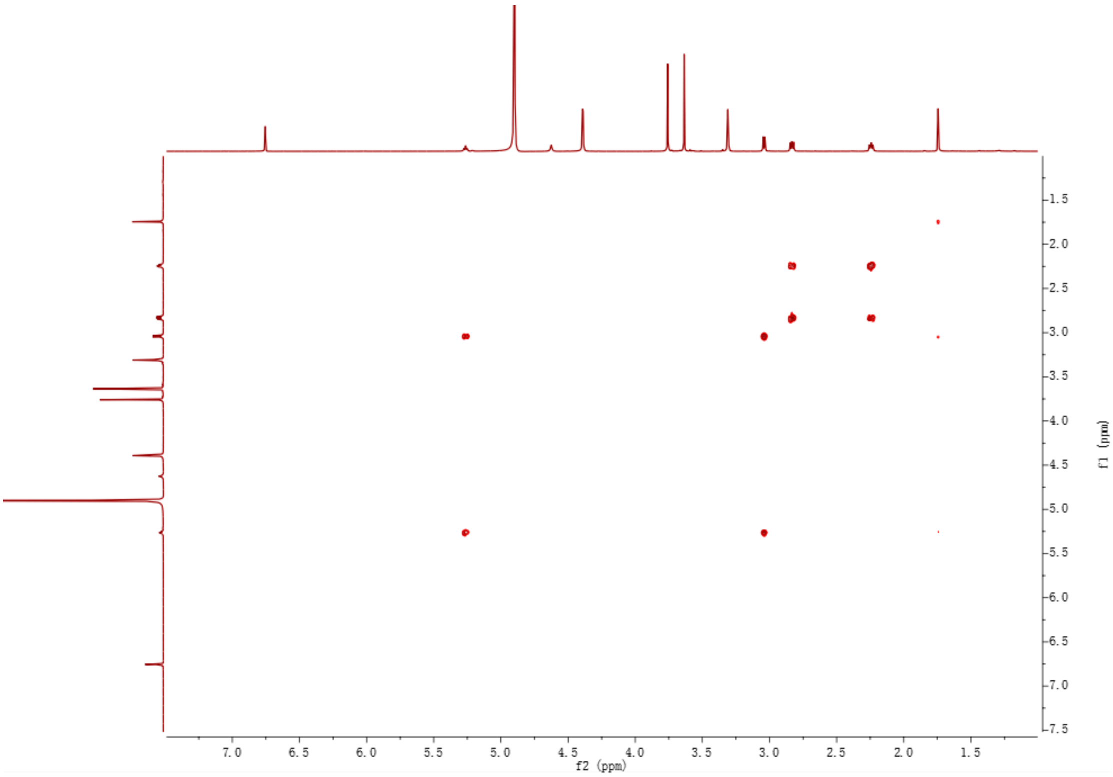

Figure S26. HSQC spectrum of compound 4.

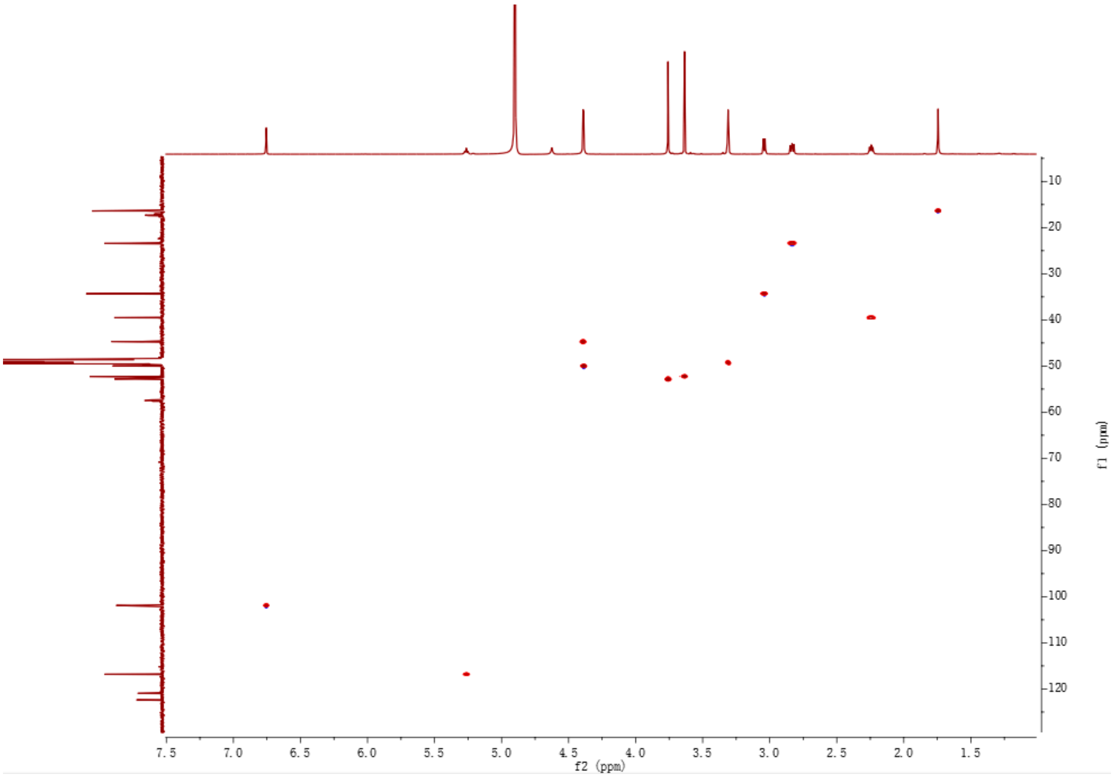

Figure S27. HMBC spectrum of compound 4.

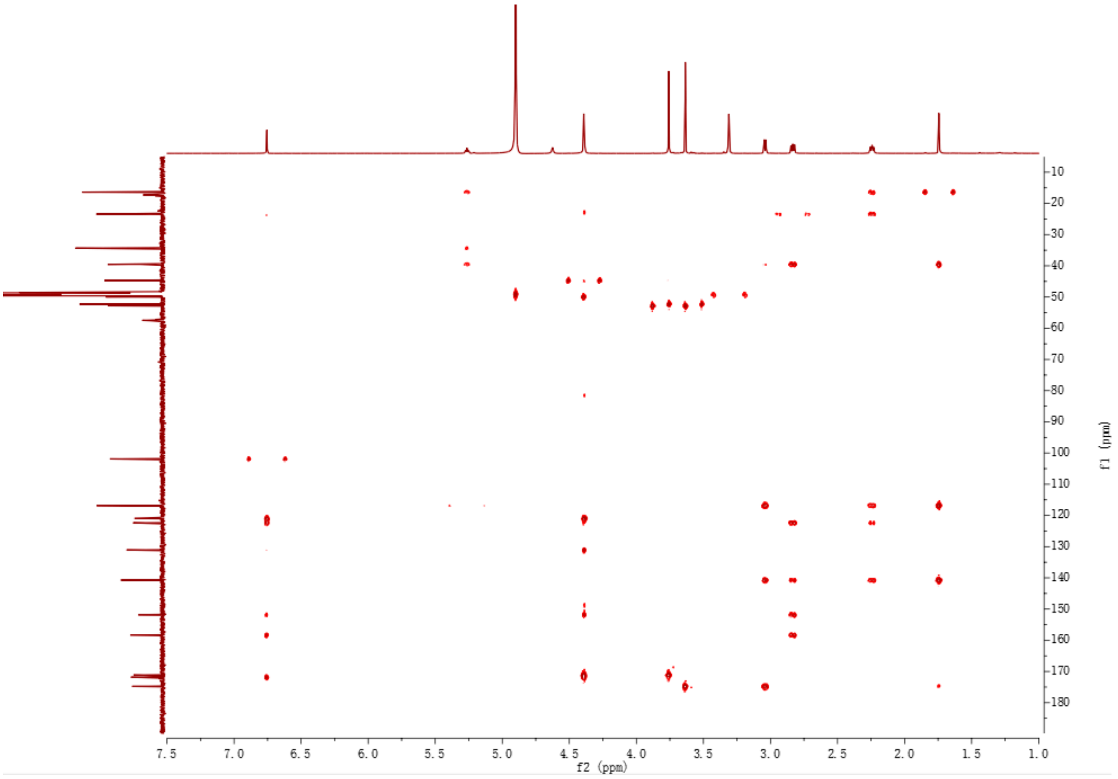

Figure S28. ROESY spectrum of compound 4.

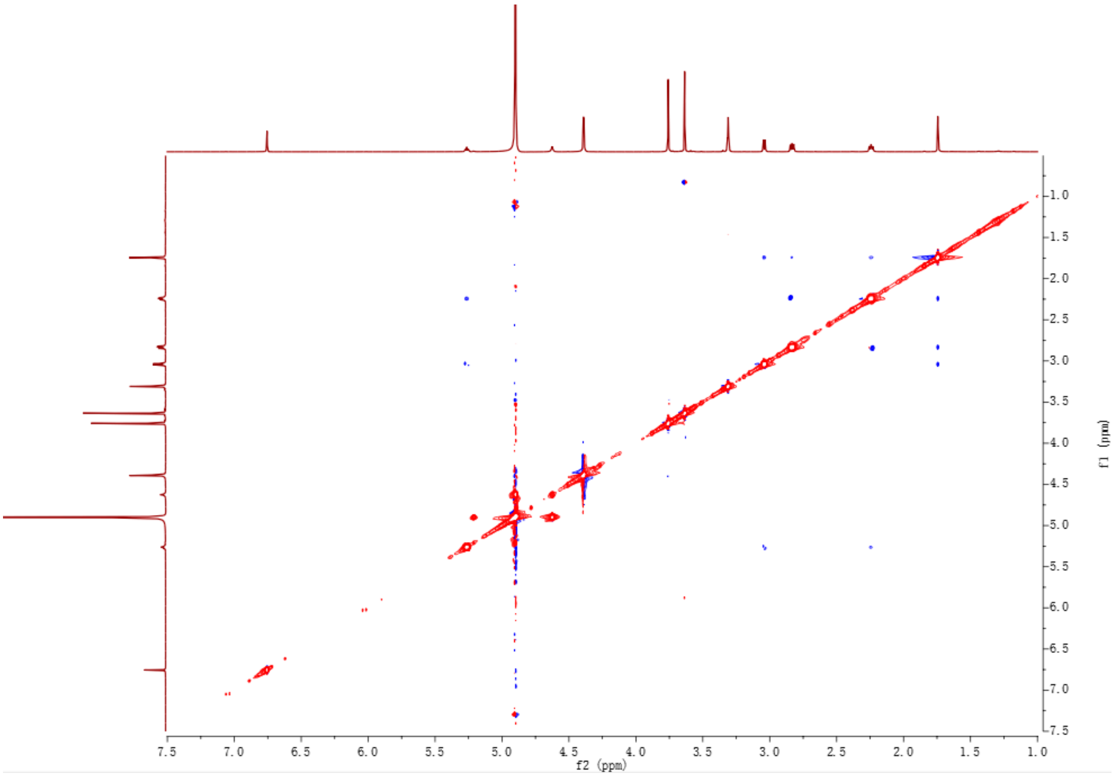

Figure S29. HRESIMS spectroscopic data of compound 5.

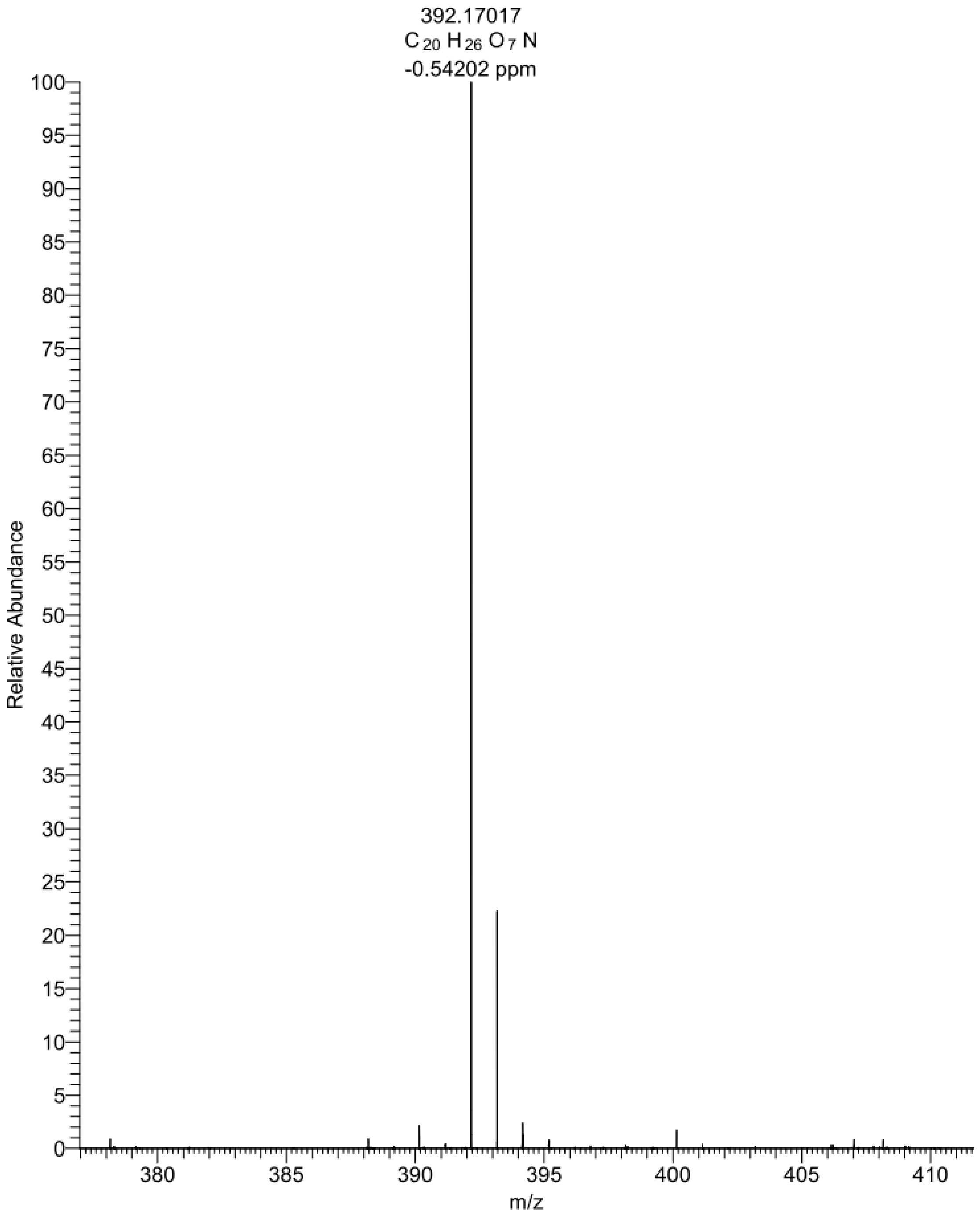

Figure S30. ^{1}H NMR (600 MHz) spectrum of compound 5.

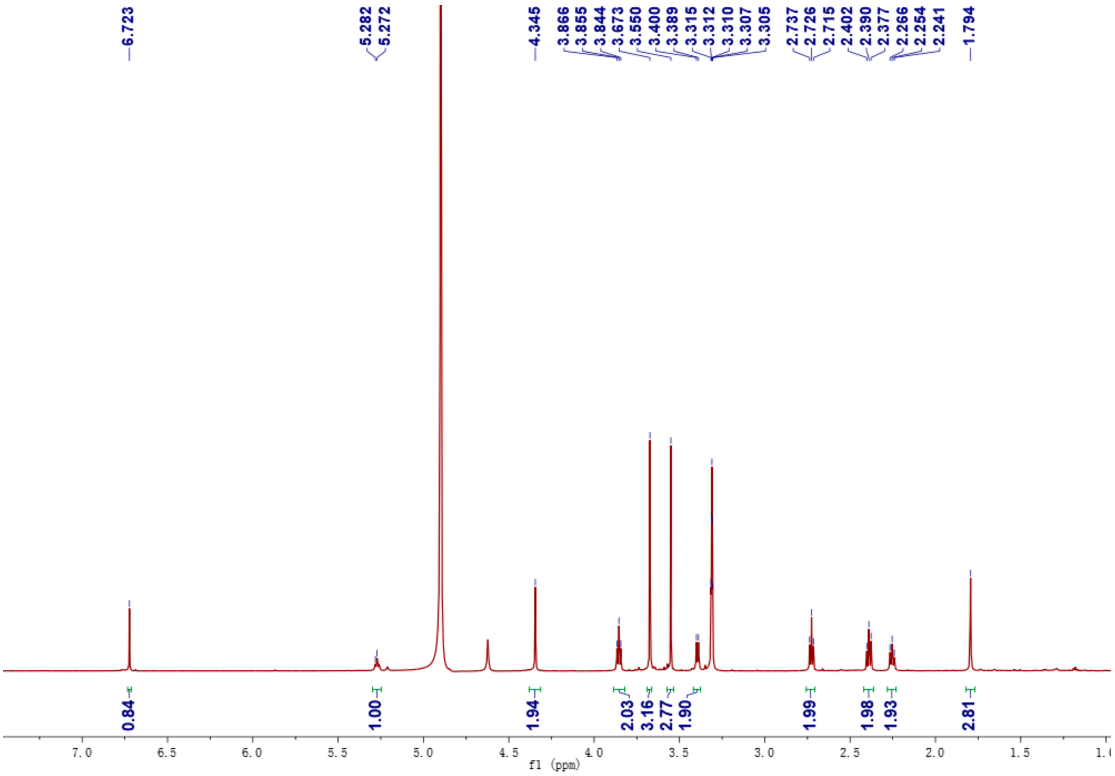

Figure S31. ^{13}C NMR and DEPT (150 MHz) spectra of compound 5.

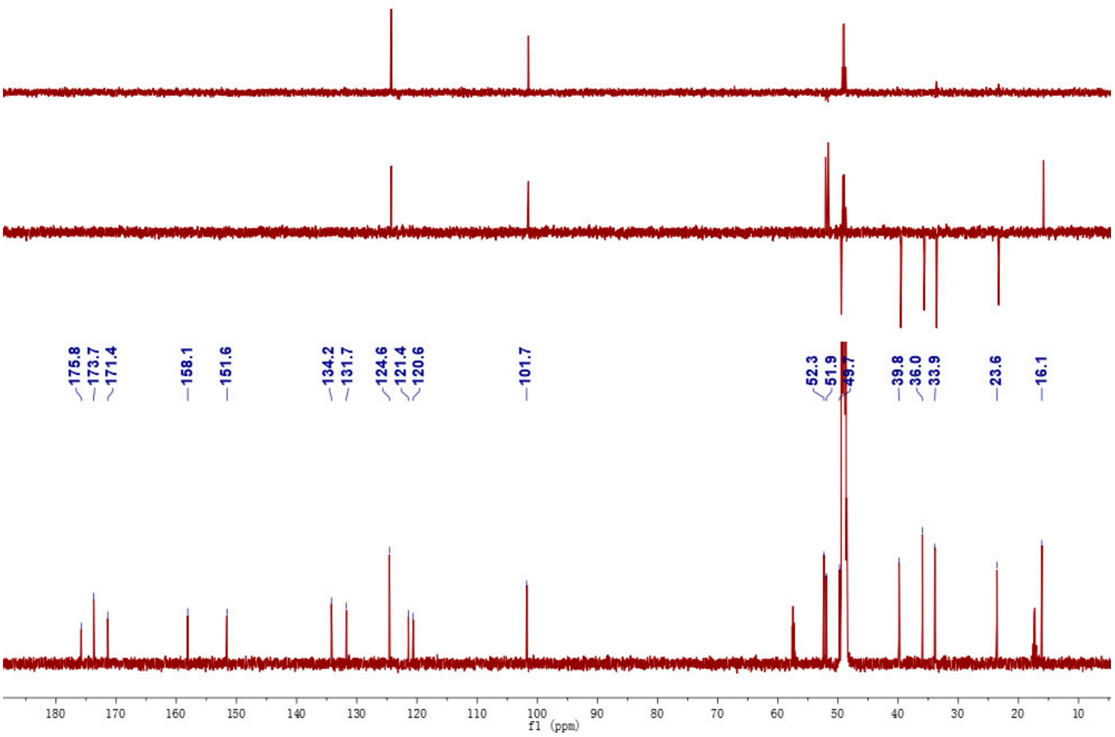

Figure S32. ^{1}H-^{1}H COSY spectrum of compound 5.

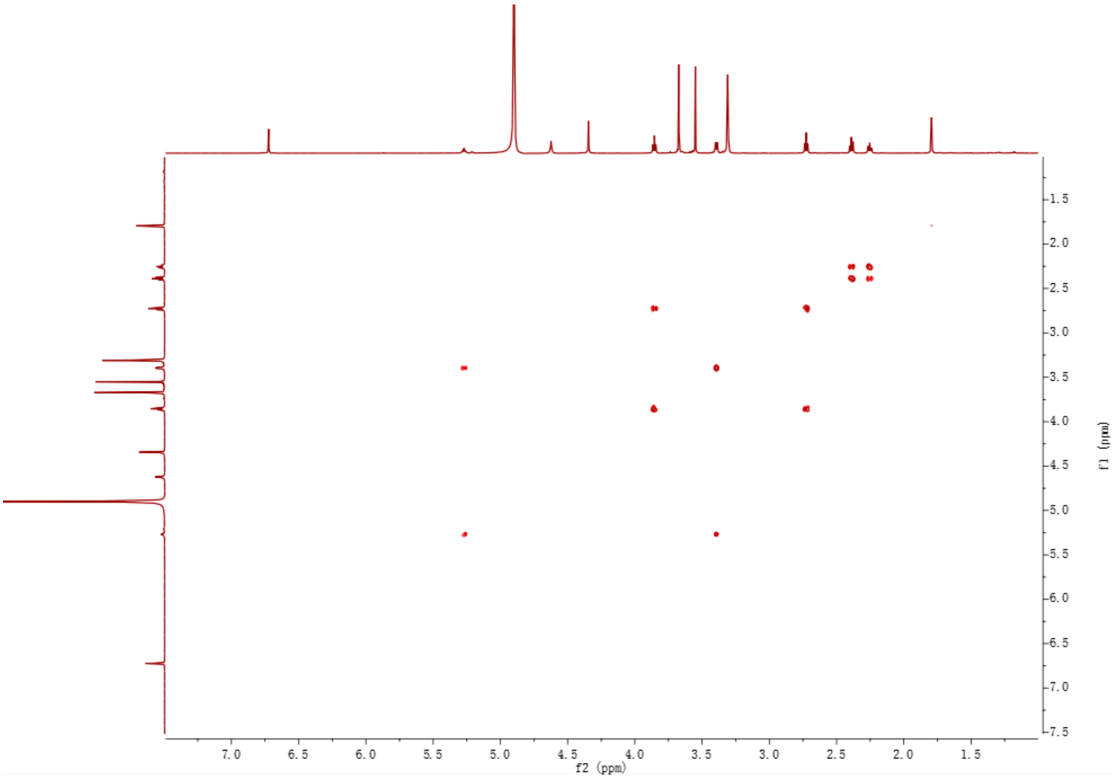

Figure S33. HSQC spectrum of compound 5.

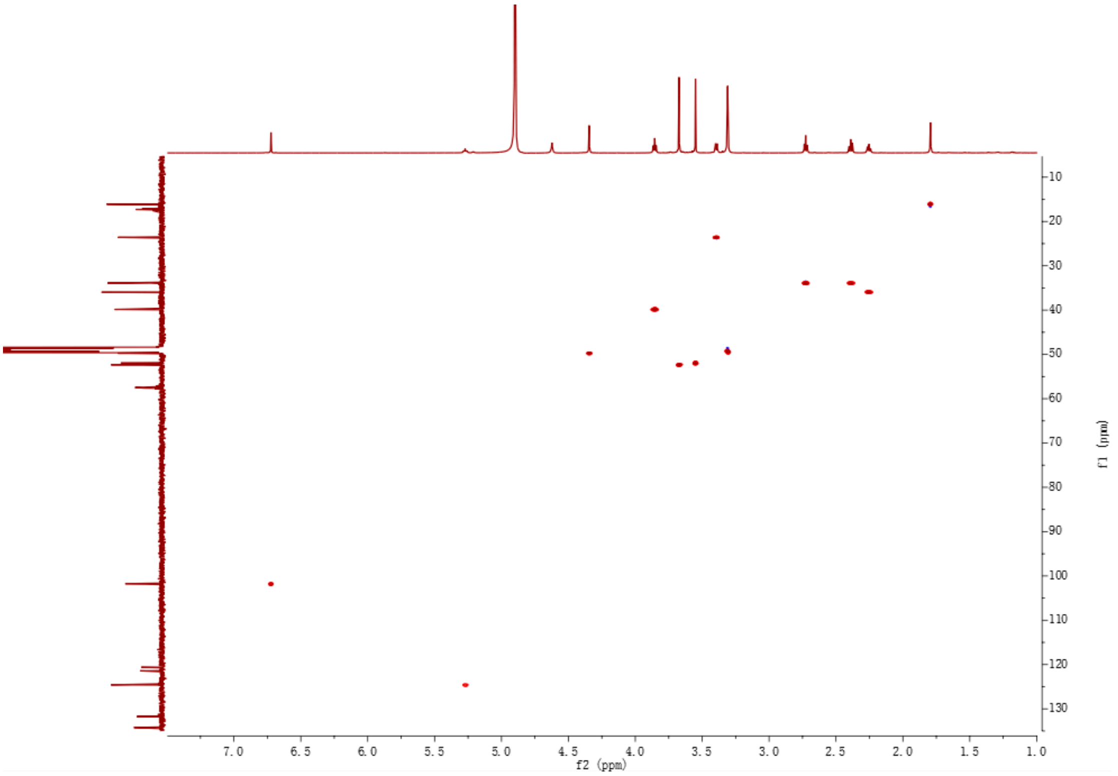

Figure S34. HMBC spectrum of compound 5.

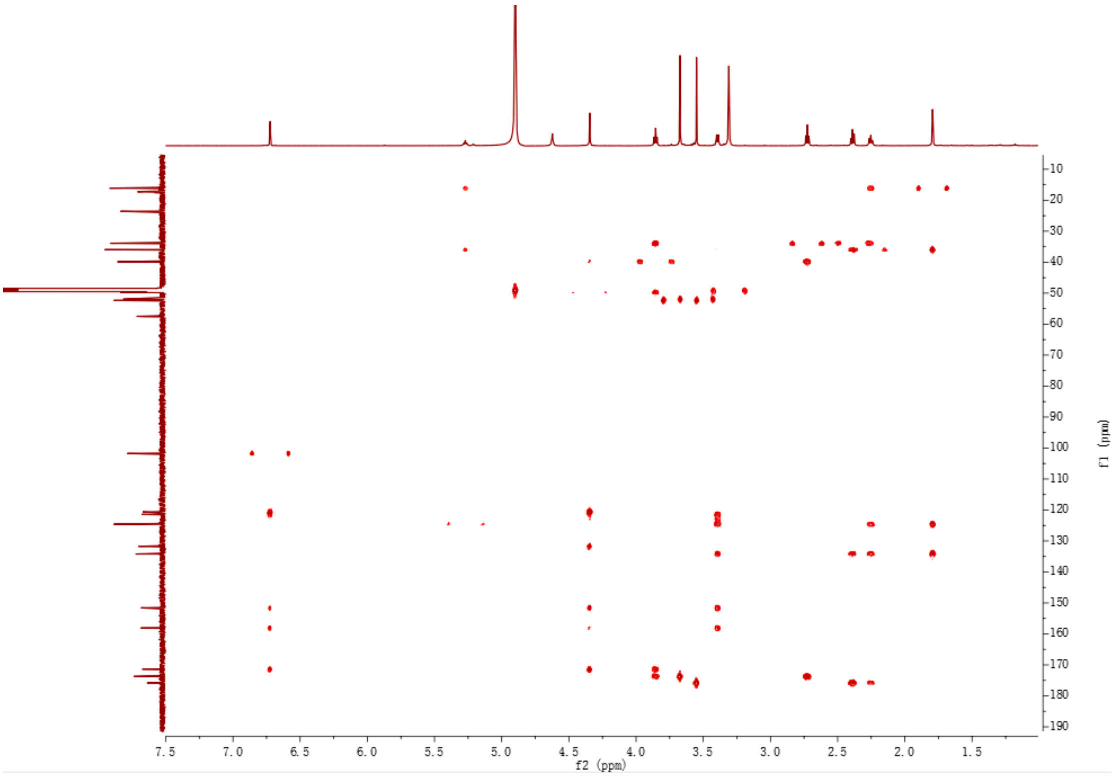

Figure S35. ROESY spectrum of compound 5.

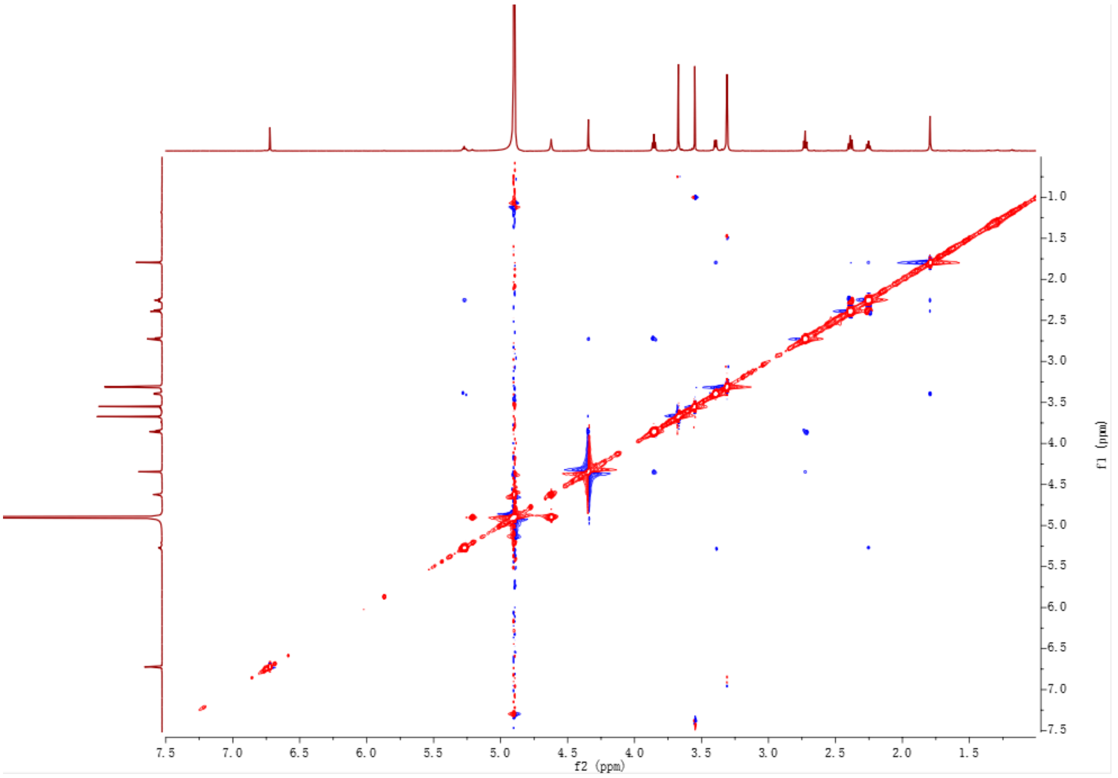

Figure S36. HRESIMS spectroscopic data of compound 6.

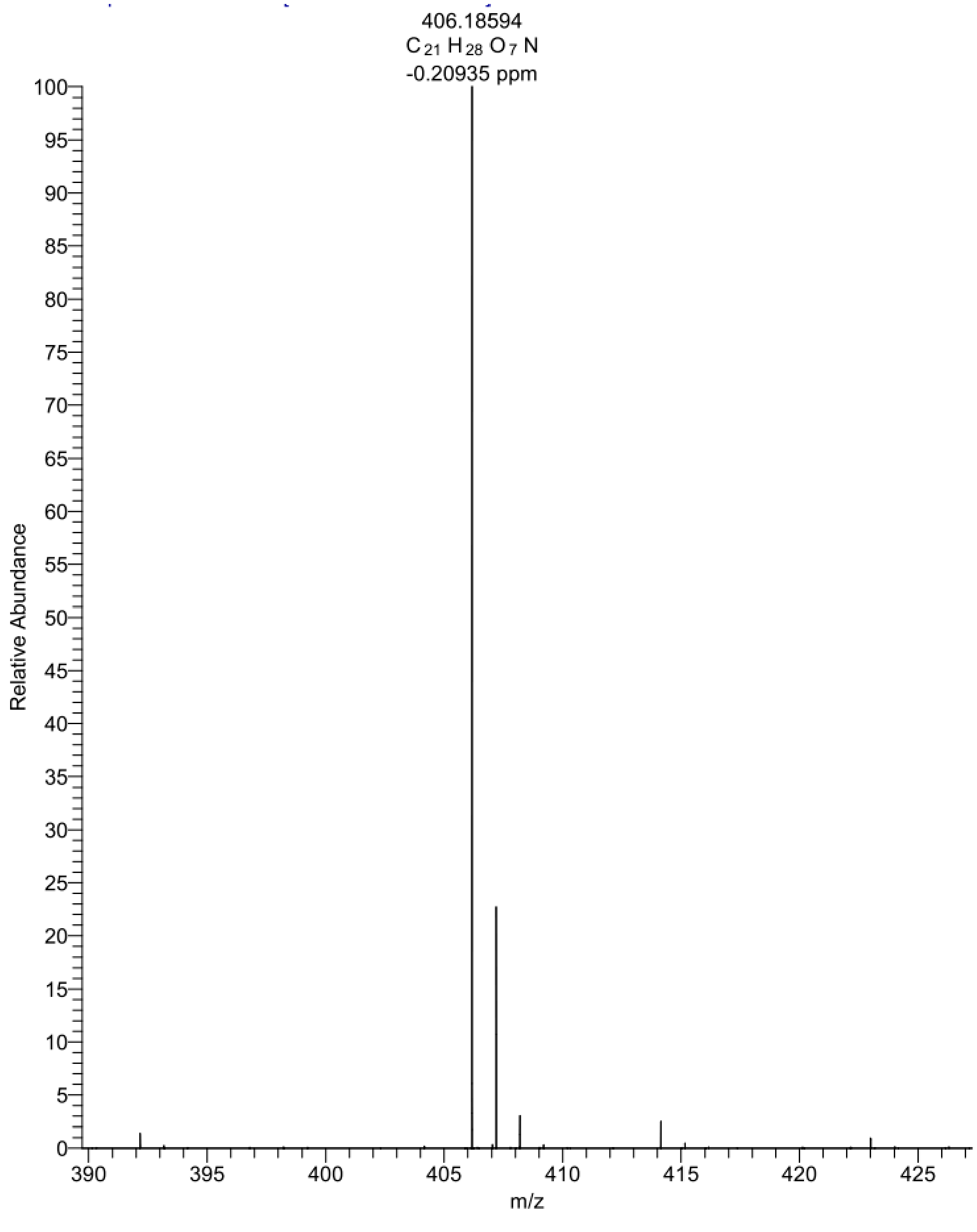

Figure S37. ^{1}H NMR (600 MHz) spectrum of compound 6.

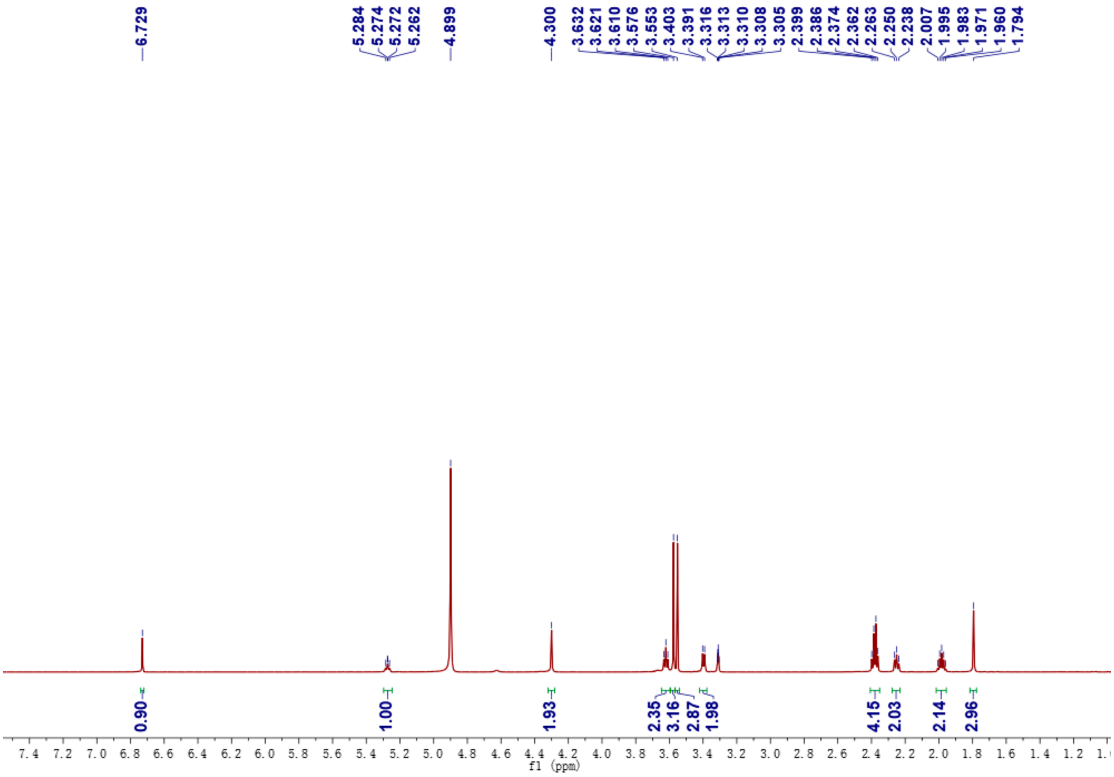

Figure S38. ^{13}C NMR and DEPT (150 MHz) spectra of compound 6.

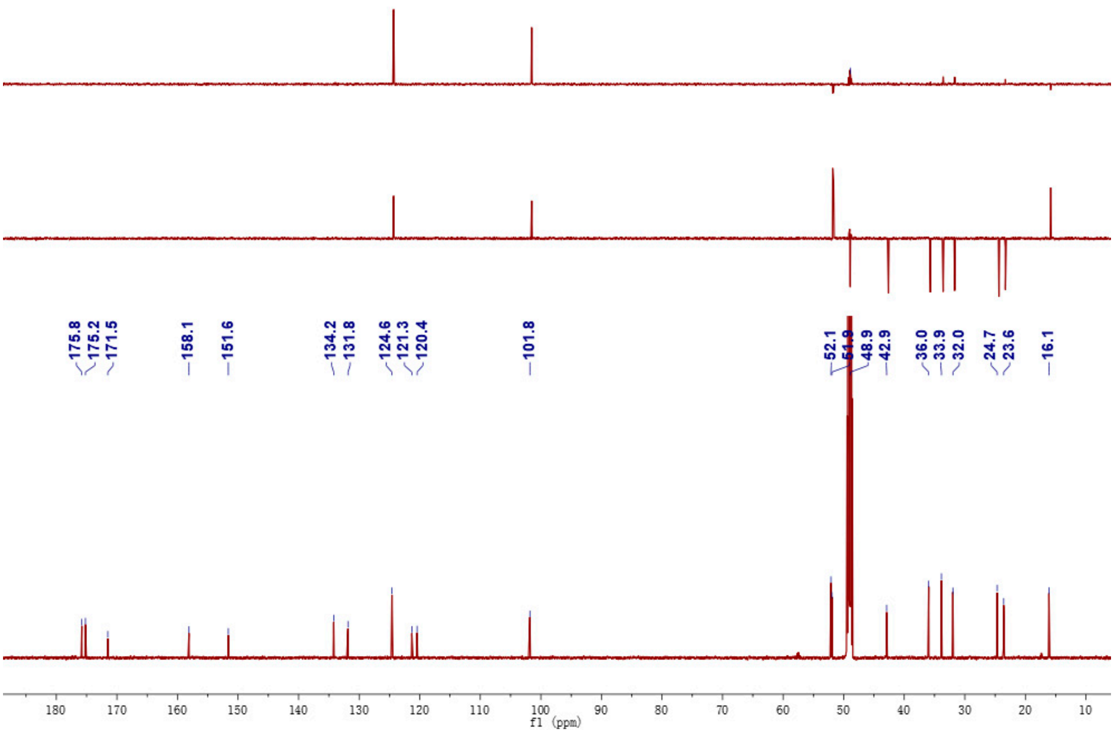

Figure S39. ^{1}H-^{1}H COSY spectrum of compound 6.

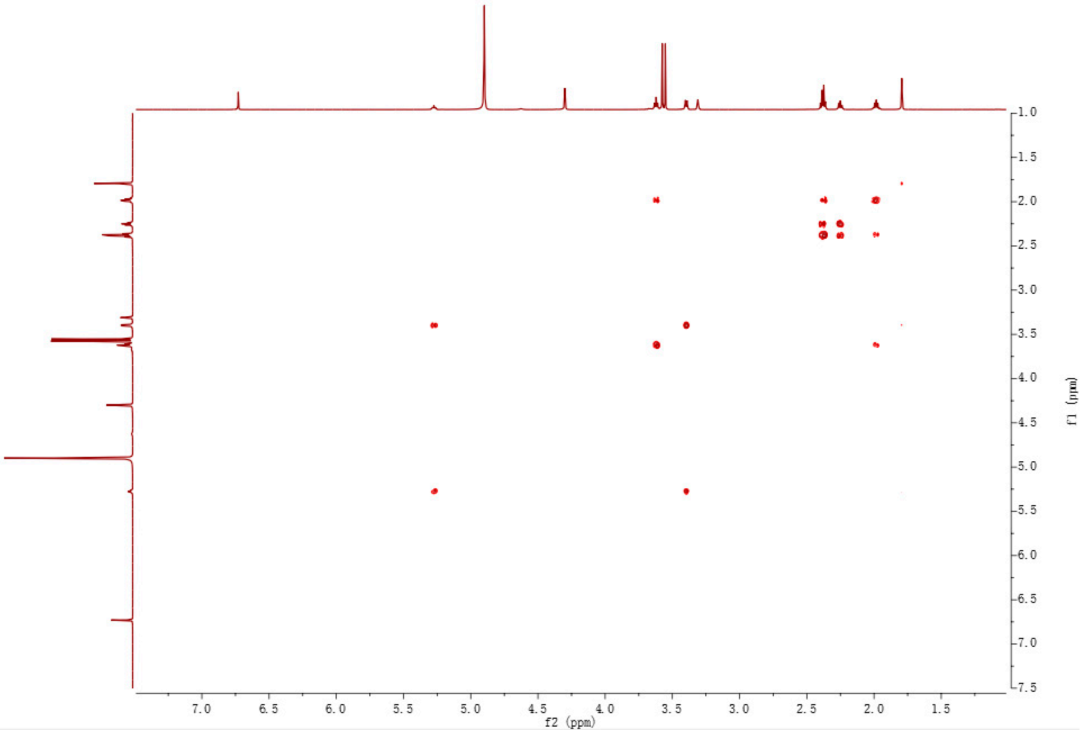

Figure S40. HSQC spectrum of compound 6.

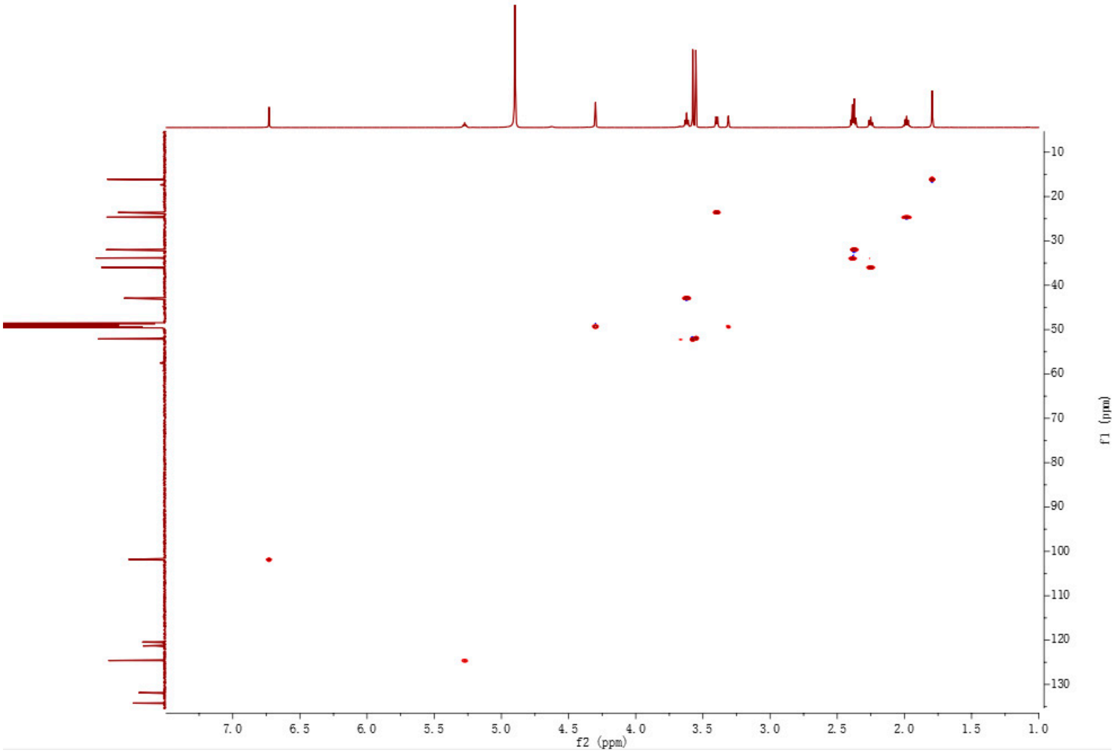

Figure S41. HMBC spectrum of compound 6.

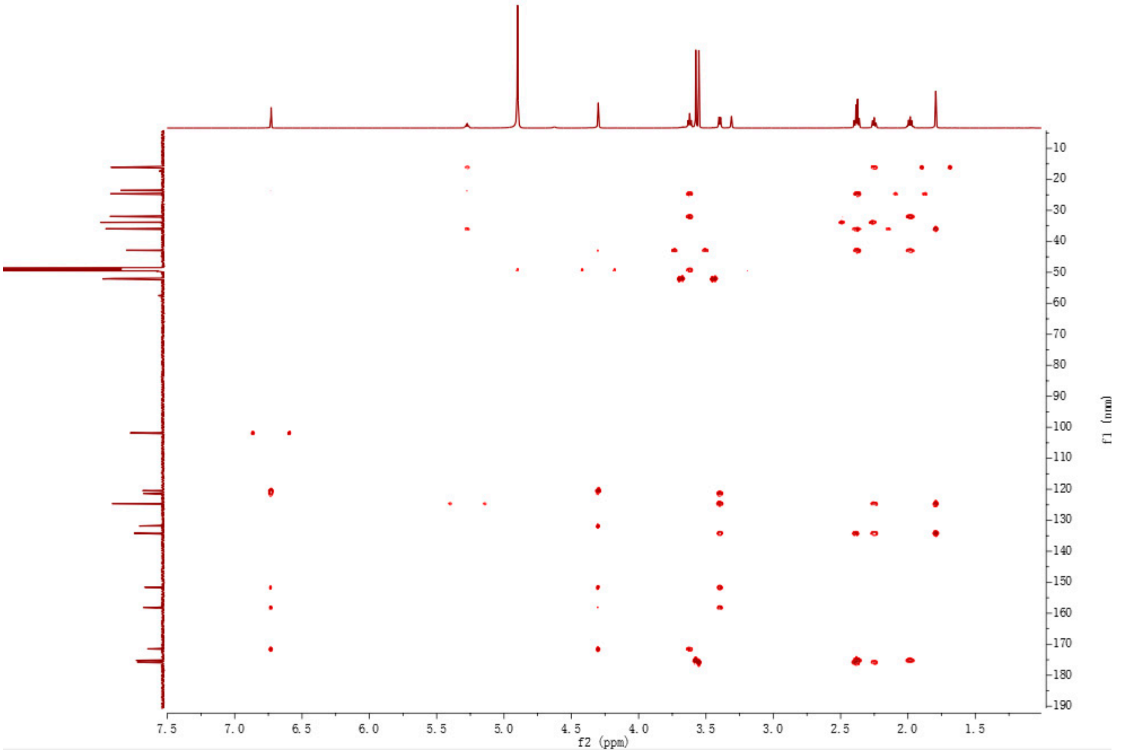

Figure S42. ROESY spectrum of compound 6.

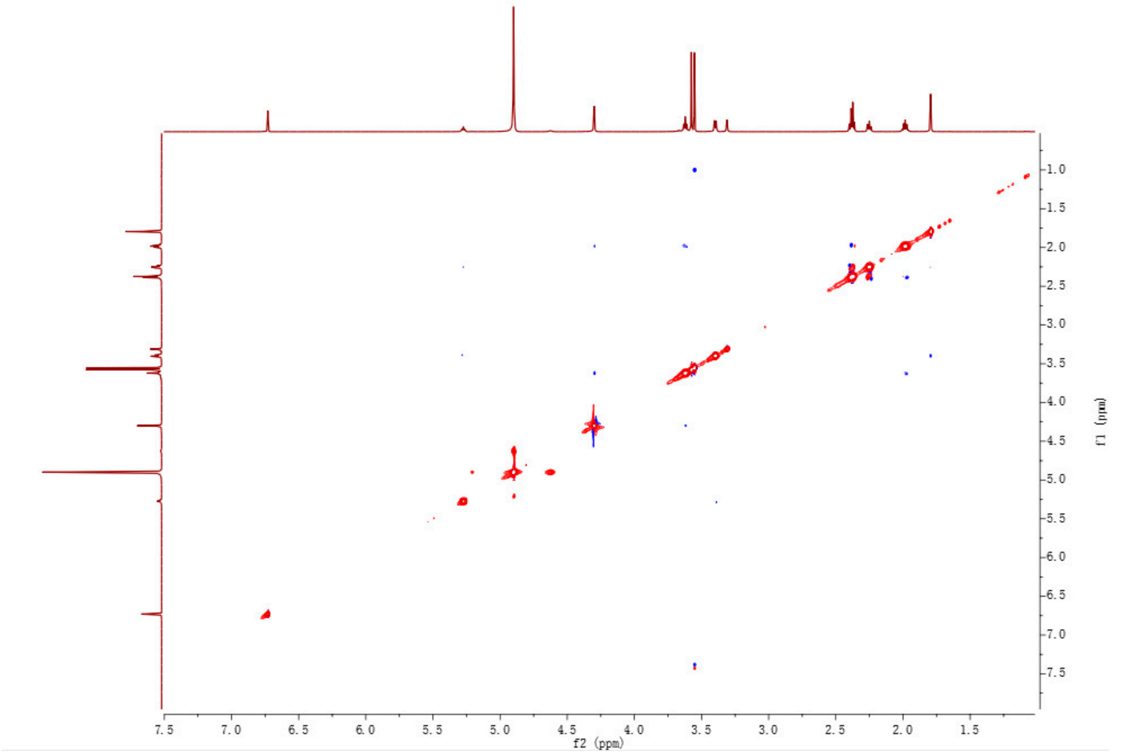

Figure S43. HRESIMS spectroscopic data of compound 7.

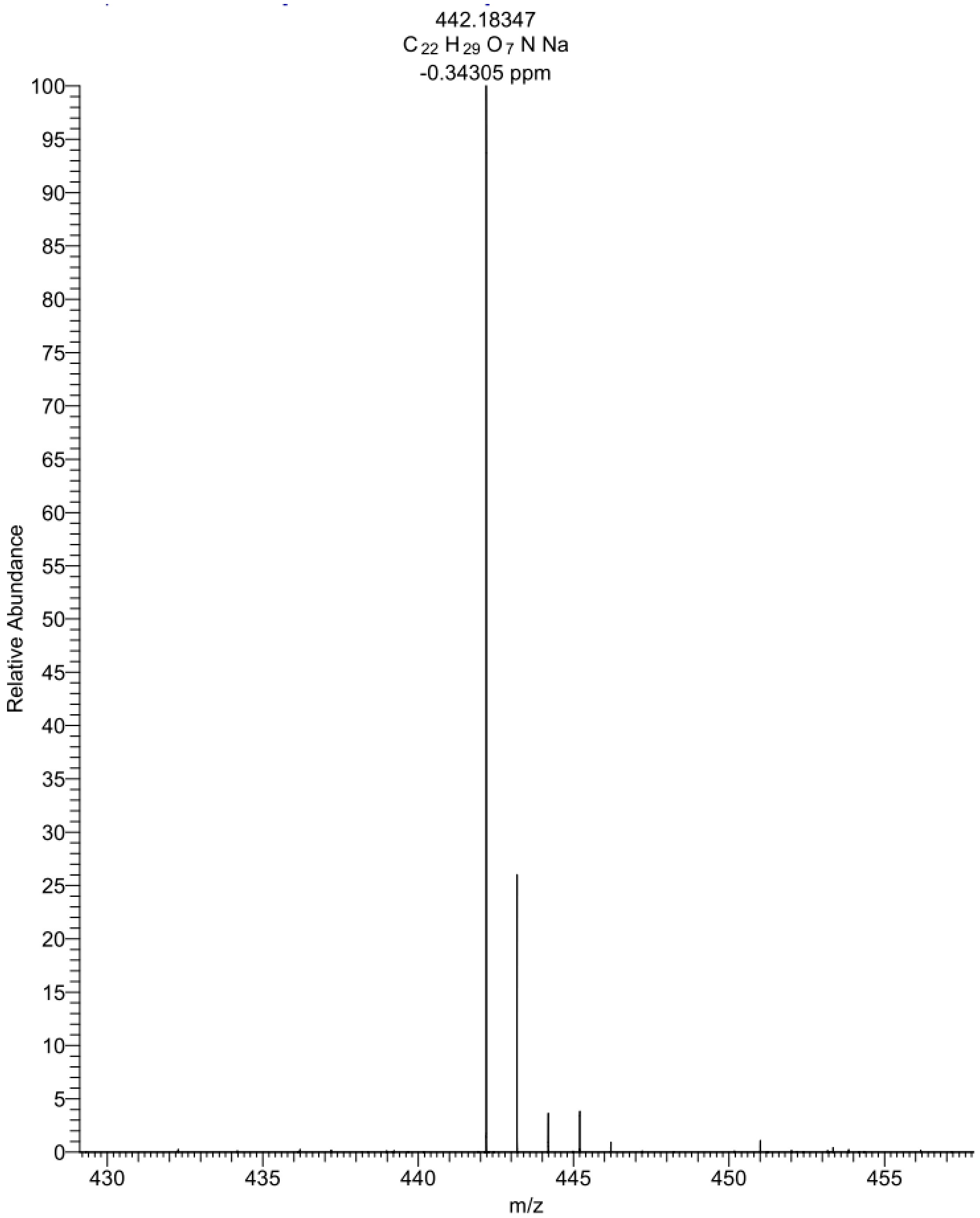

Figure S44. ^{1}H NMR (500 MHz) spectrum of compound 7.

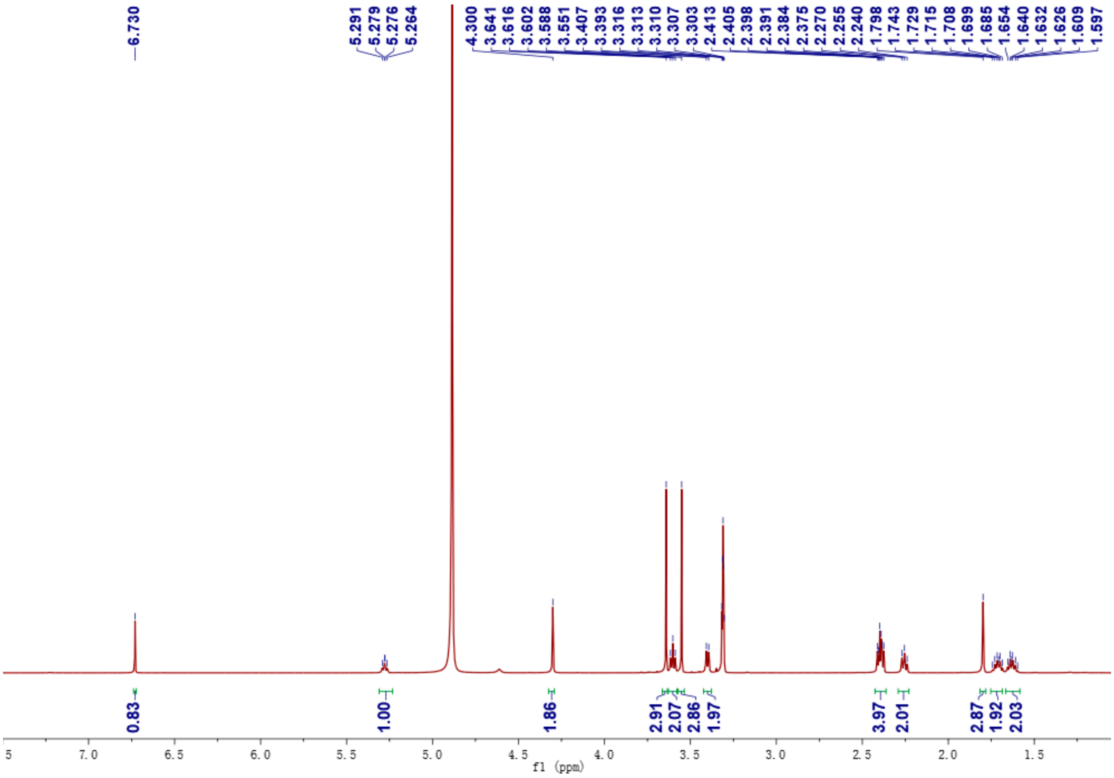

Figure S45. ^{13}C NMR and DEPT (125 MHz) spectra of compound 7.

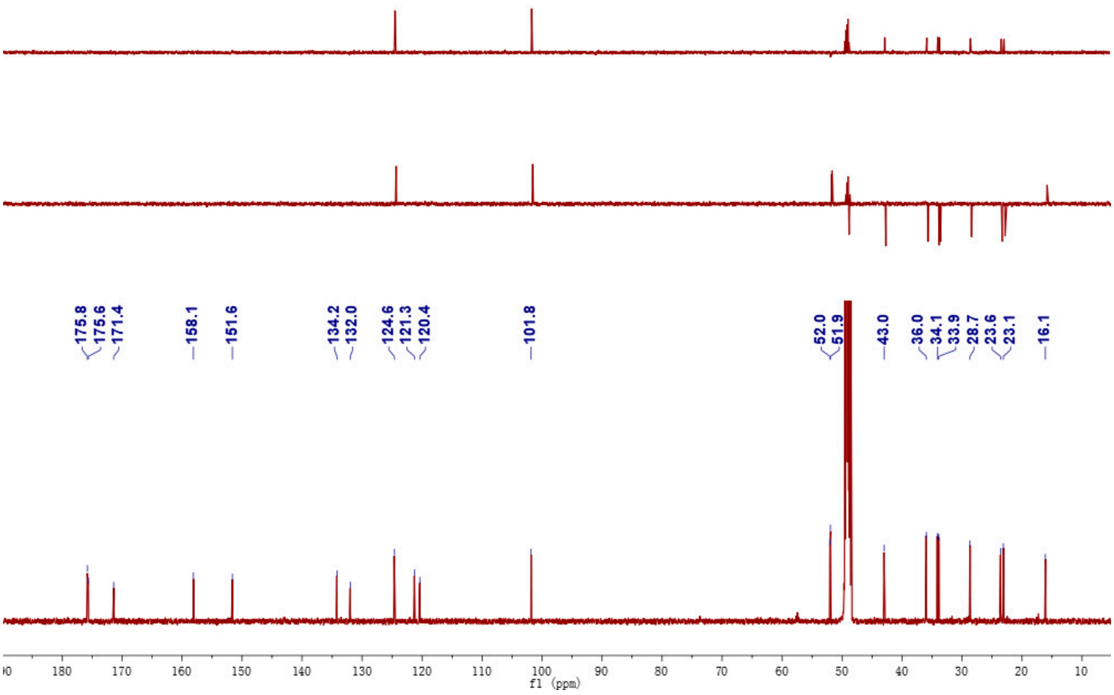

Figure S46. ^{1}H-^{1}H COSY spectrum of compound 7.

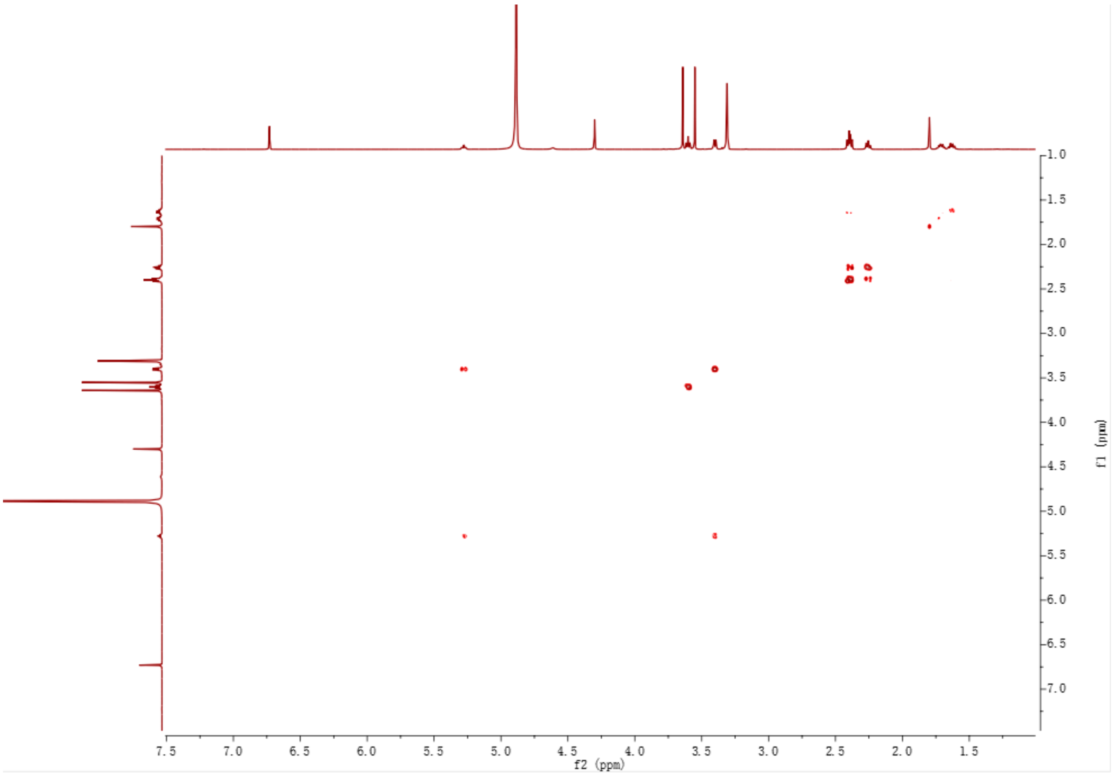

Figure S47. HSQC spectrum of compound 7.

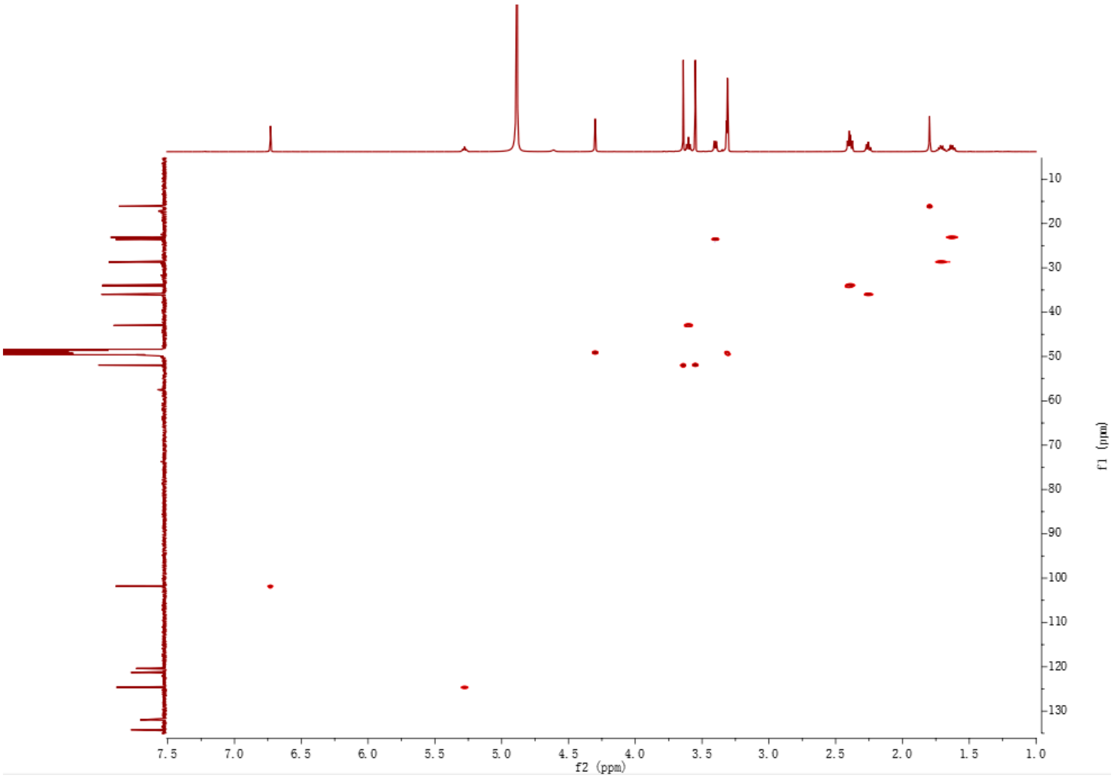

Figure S48. HMBC spectrum of compound 7.

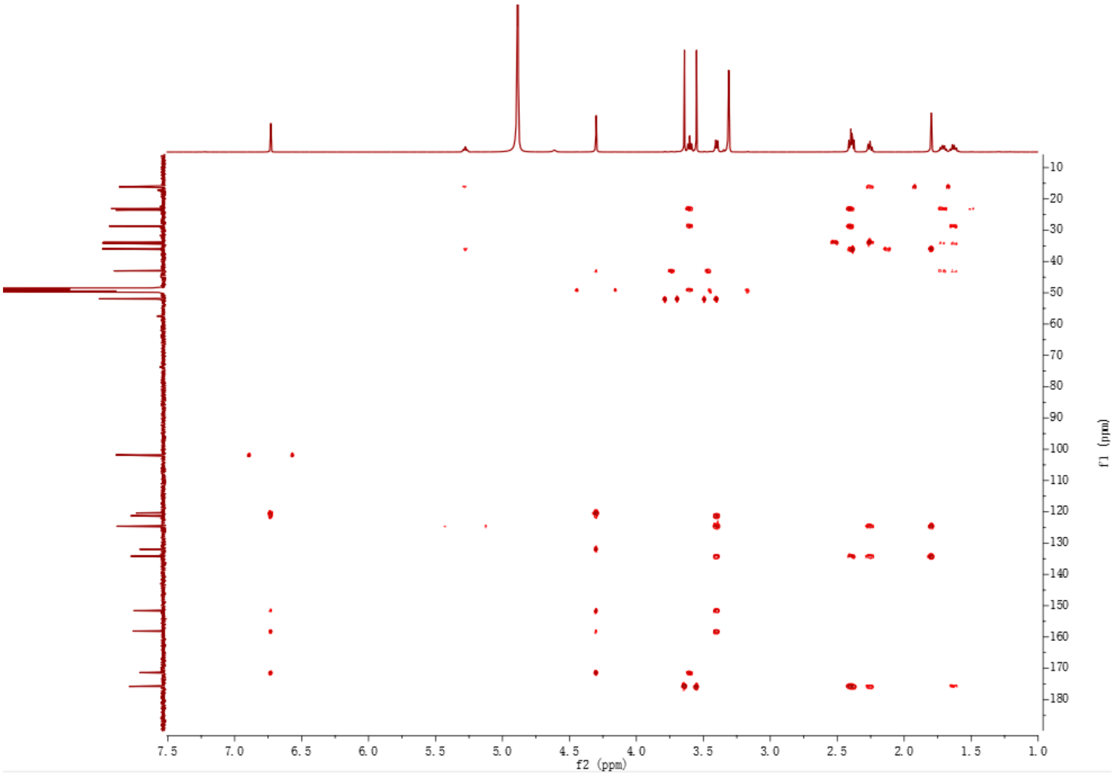

Figure S49. ROESY spectrum of compound 7.

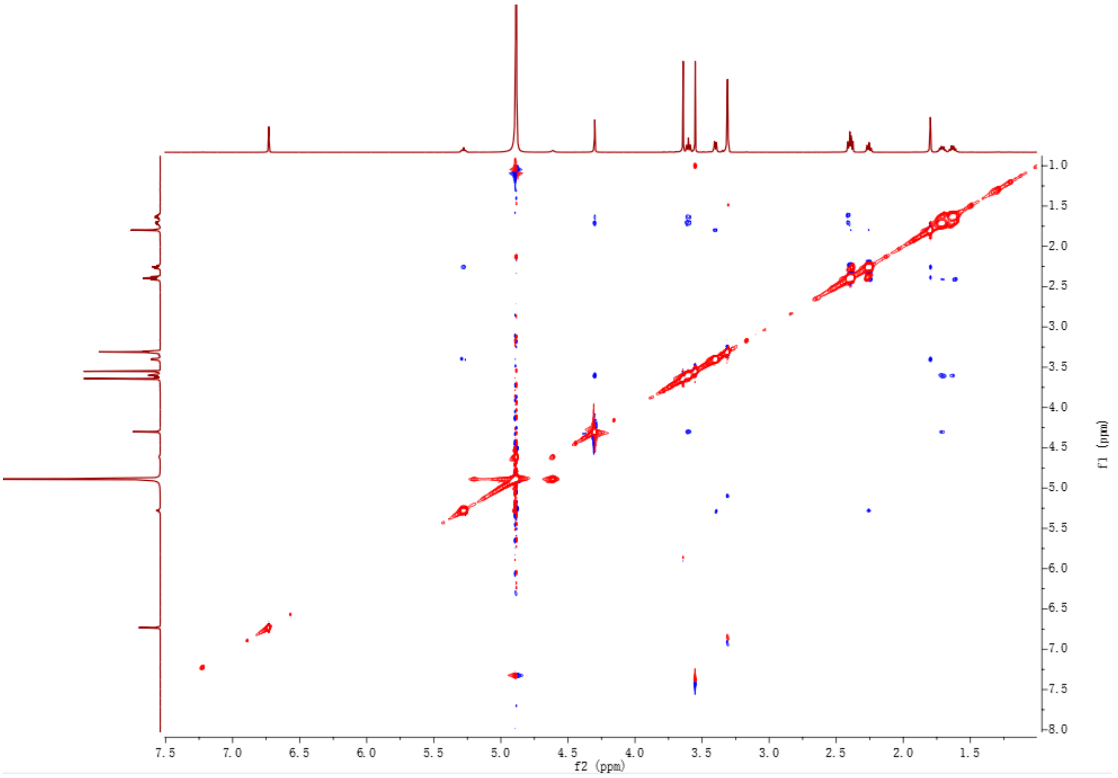

Figure S50. HRESIMS spectroscopic data of compound 8.

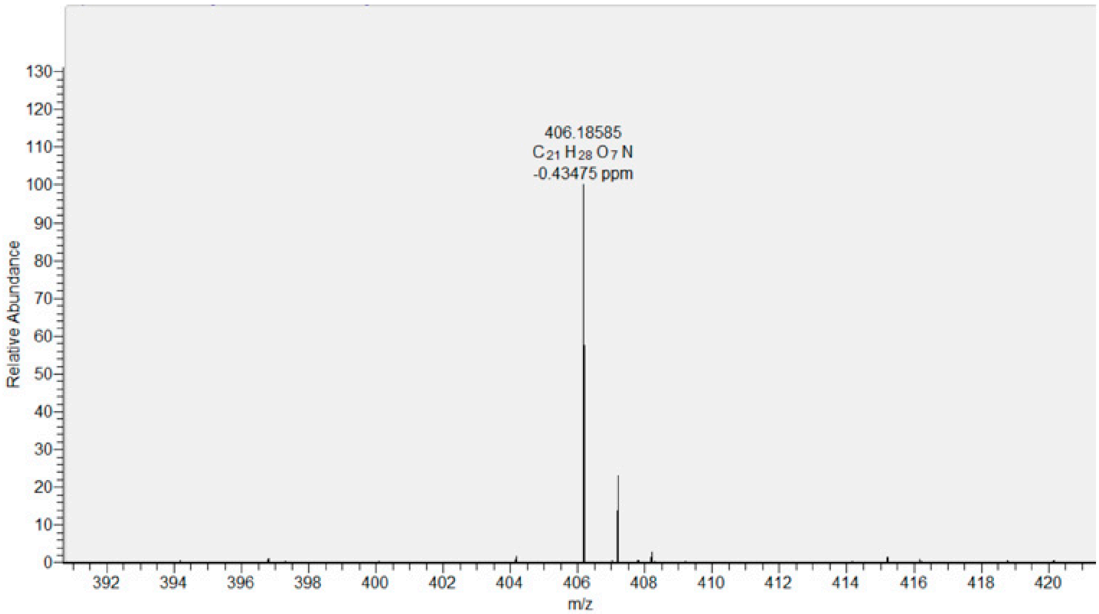

Figure S51. ^{1}H NMR (600 MHz) spectrum of compound 8.

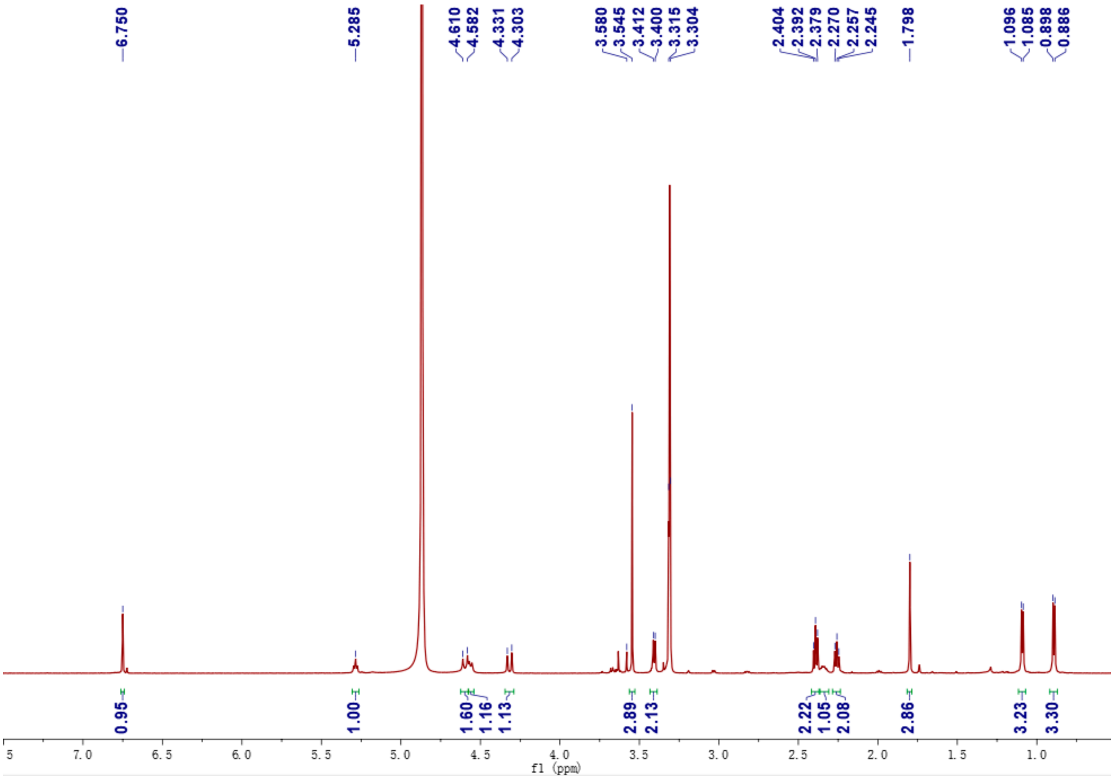

Figure S52. ^{13}C NMR and DEPT (150 MHz) spectra of compound 8.

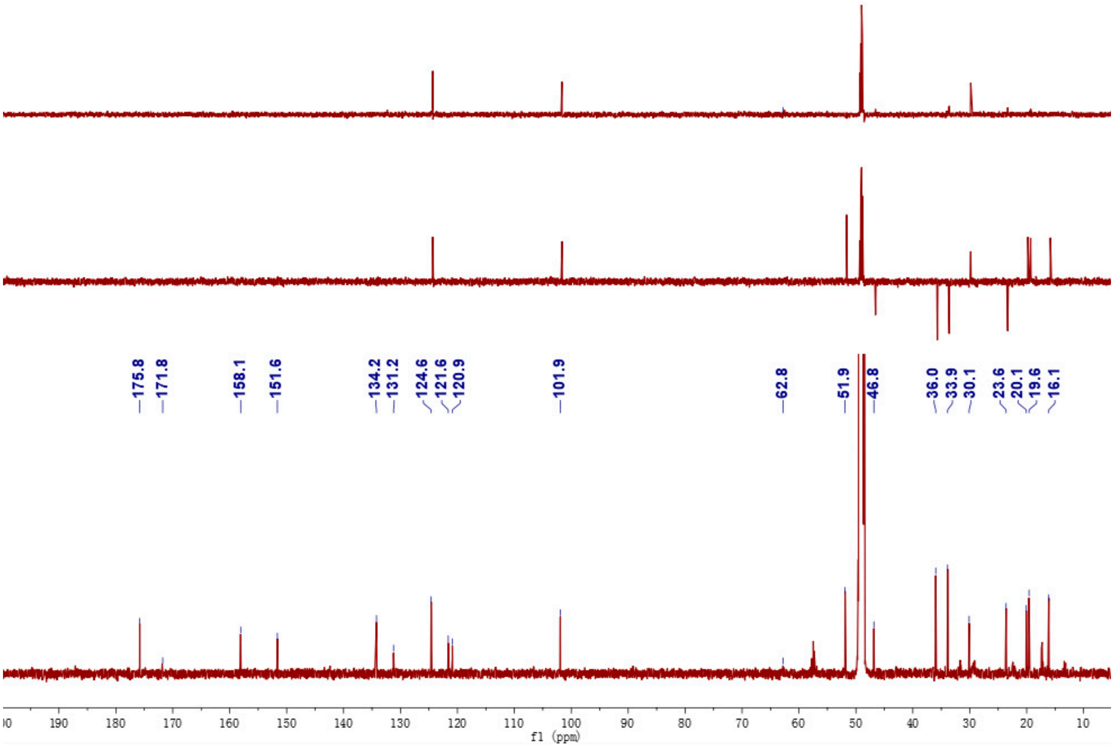

Figure S53. ^{1}H-^{1}H COSY spectrum of compound 8.

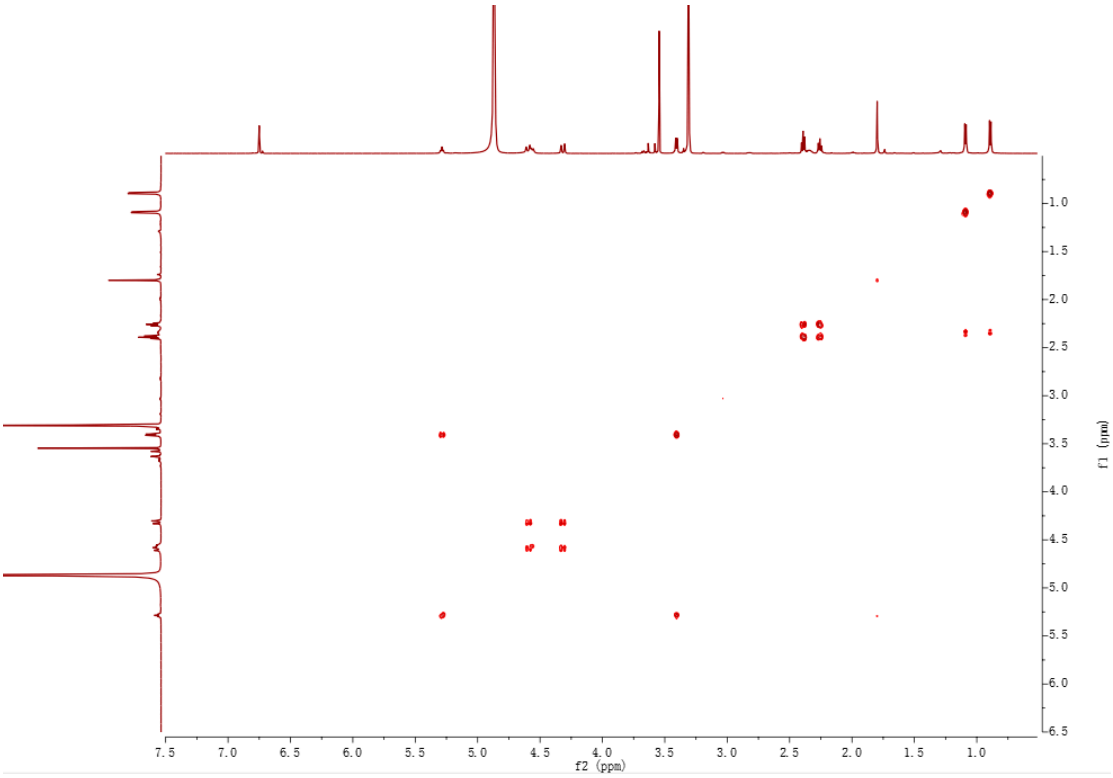

Figure S54. HSQC spectrum of compound 8.

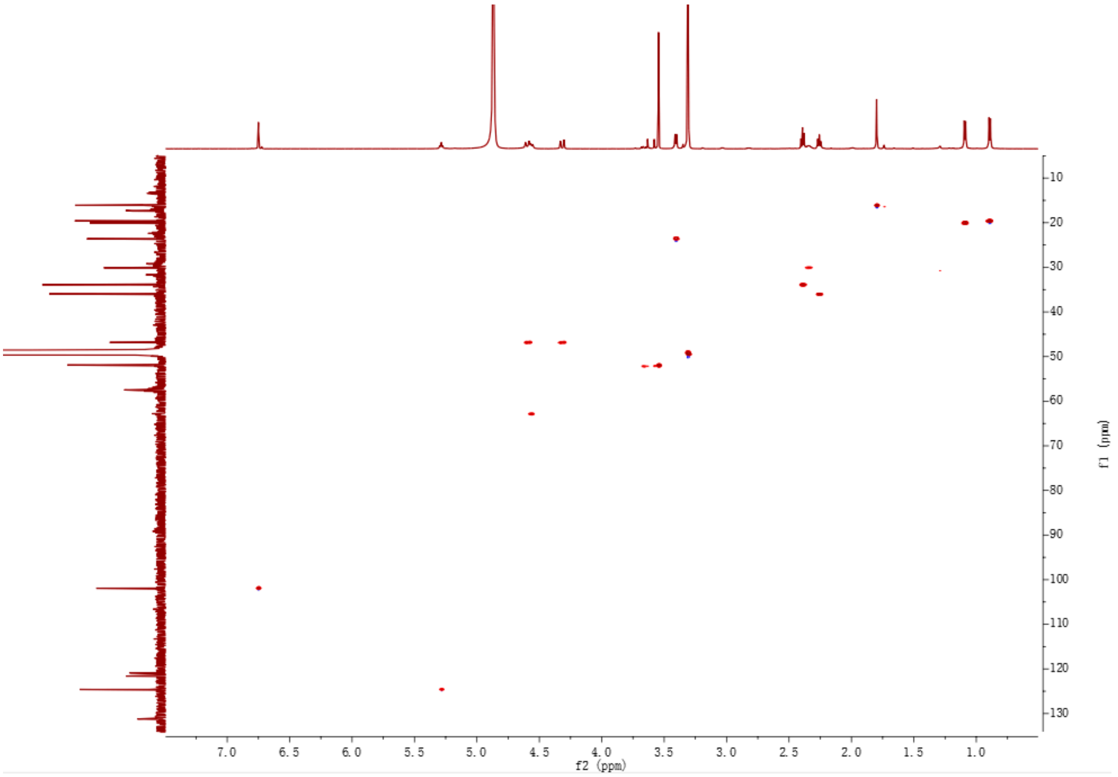

Figure S55. HMBC spectrum of compound 8.

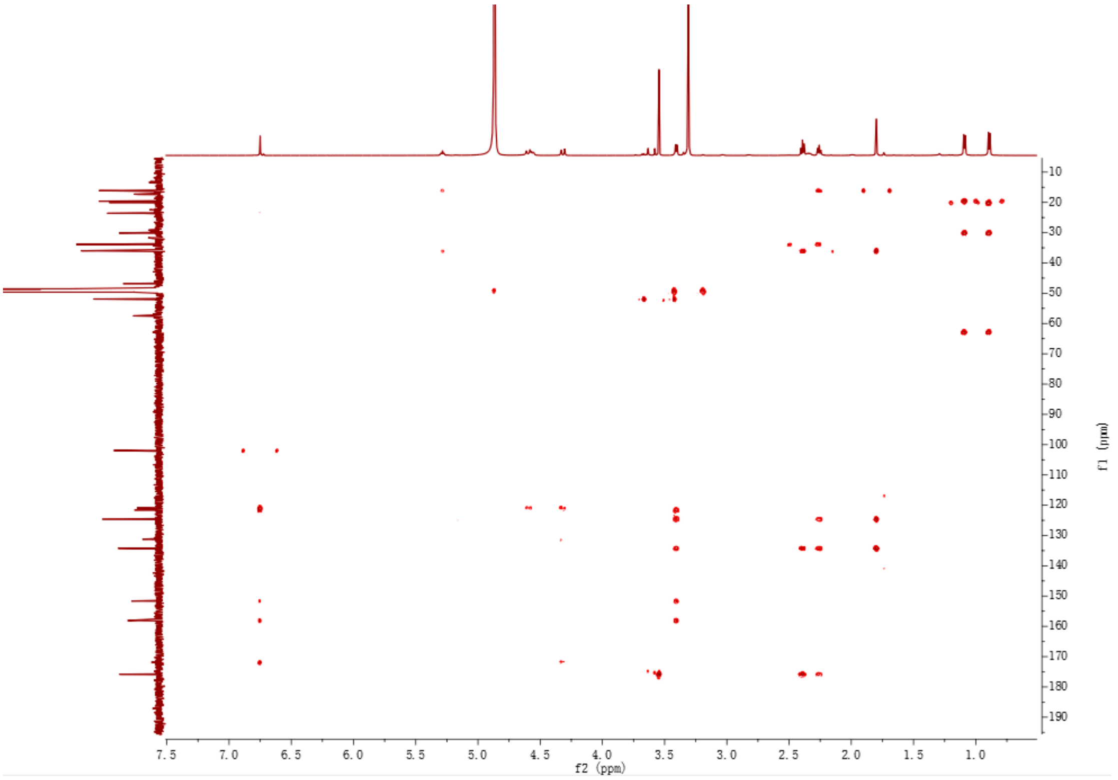

Figure S56. ROESY spectrum of compound 8.

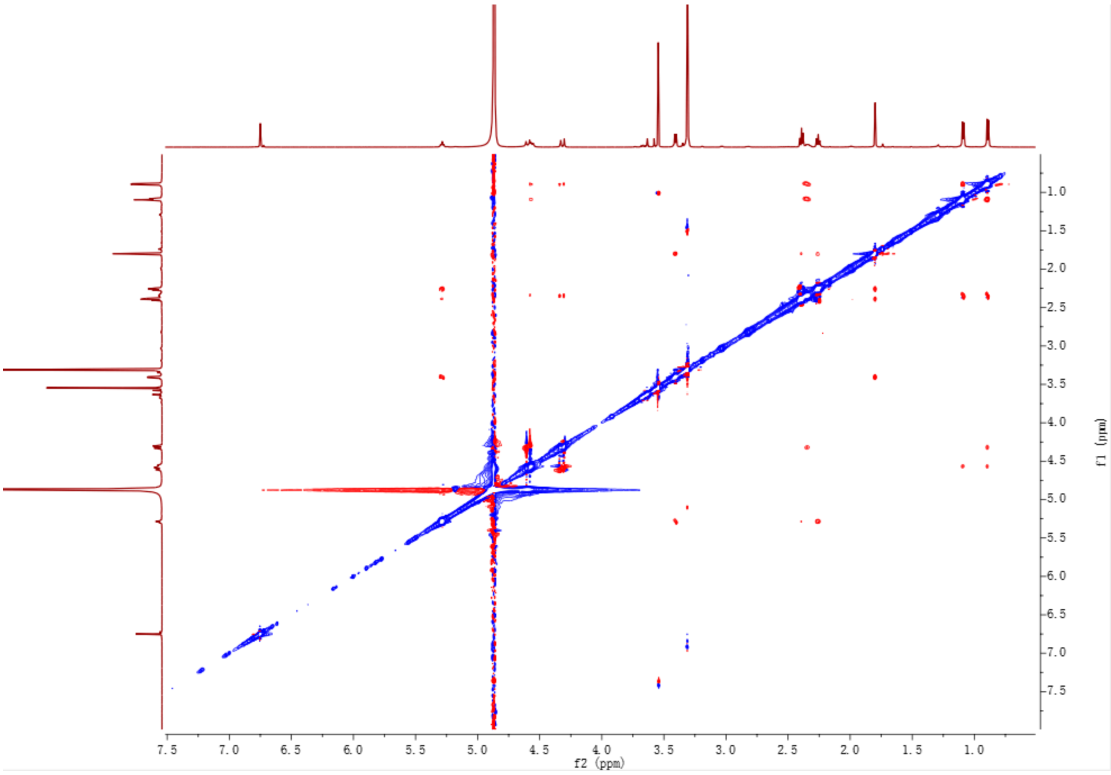

Figure S57. HRESIMS spectroscopic data of compound 9.

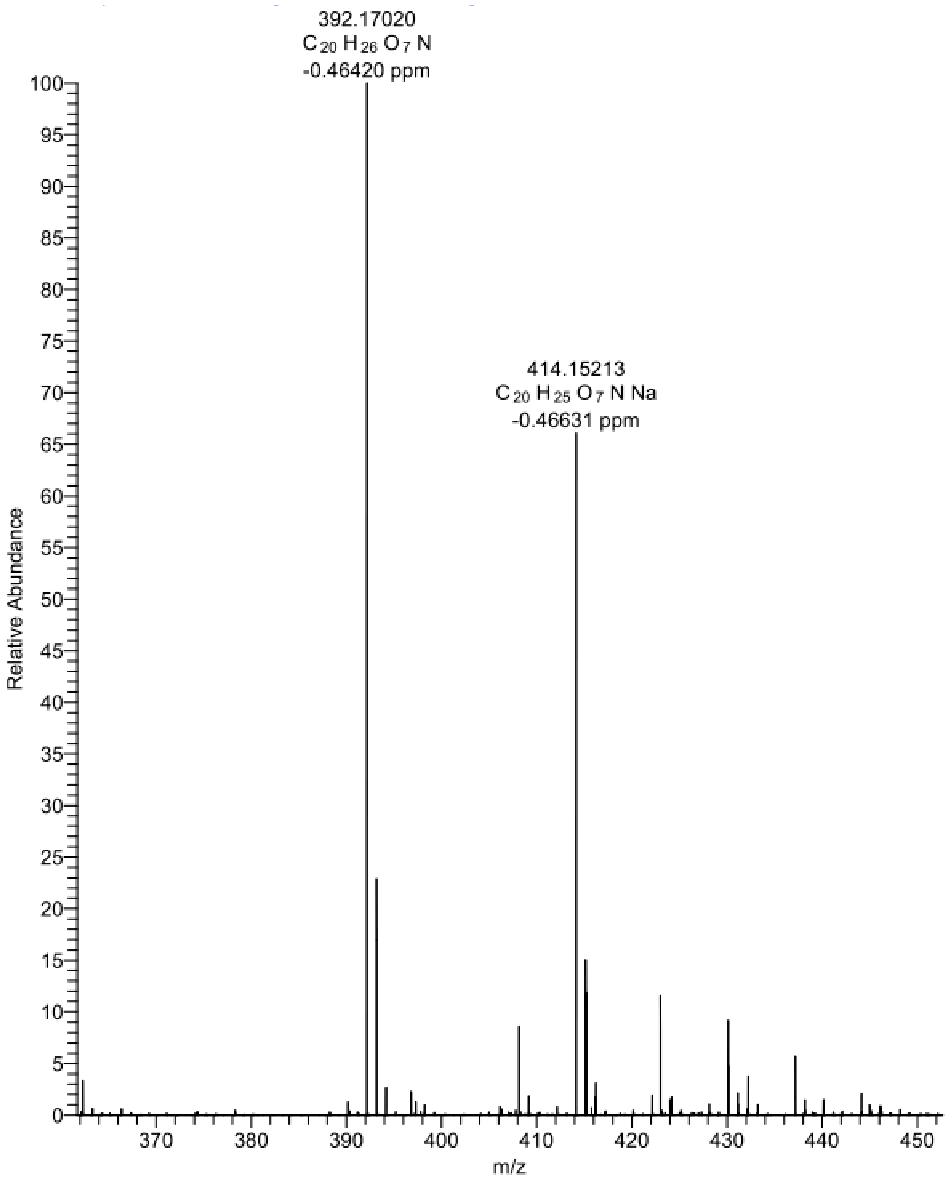

Figure S58. ^{1}H NMR (600 MHz) spectrum of compound 9.

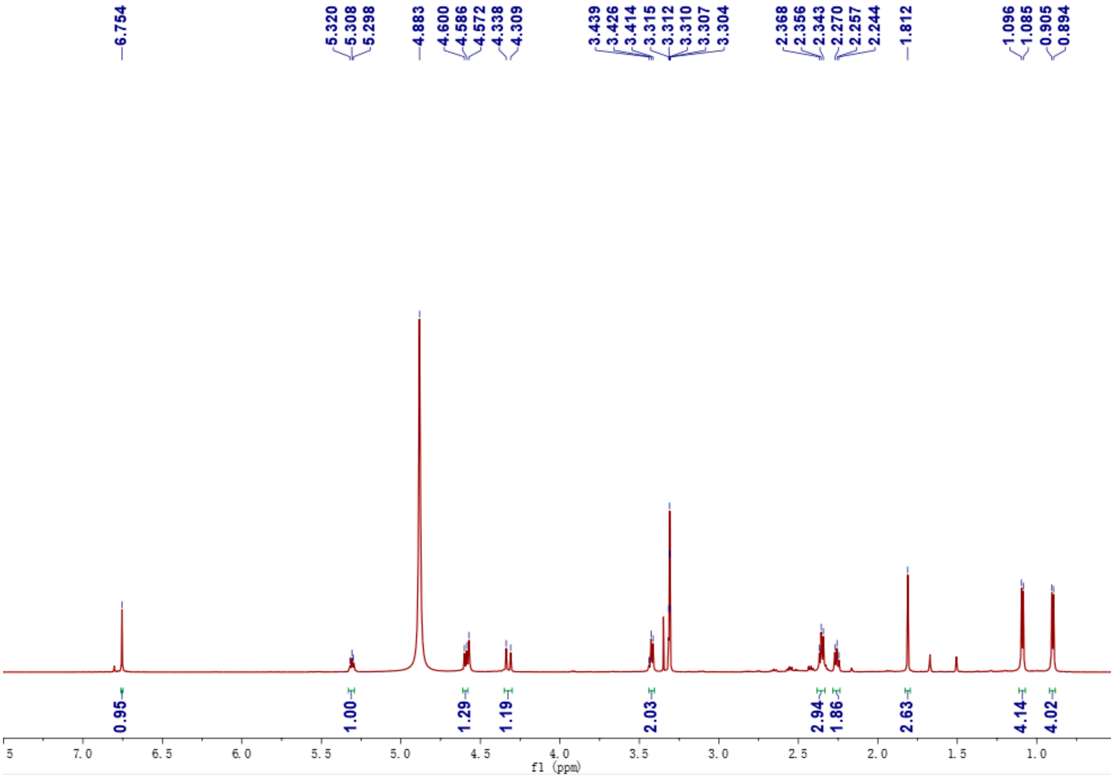

Figure S59. ^{13}C NMR and DEPT (150 MHz) spectra of compound 9.

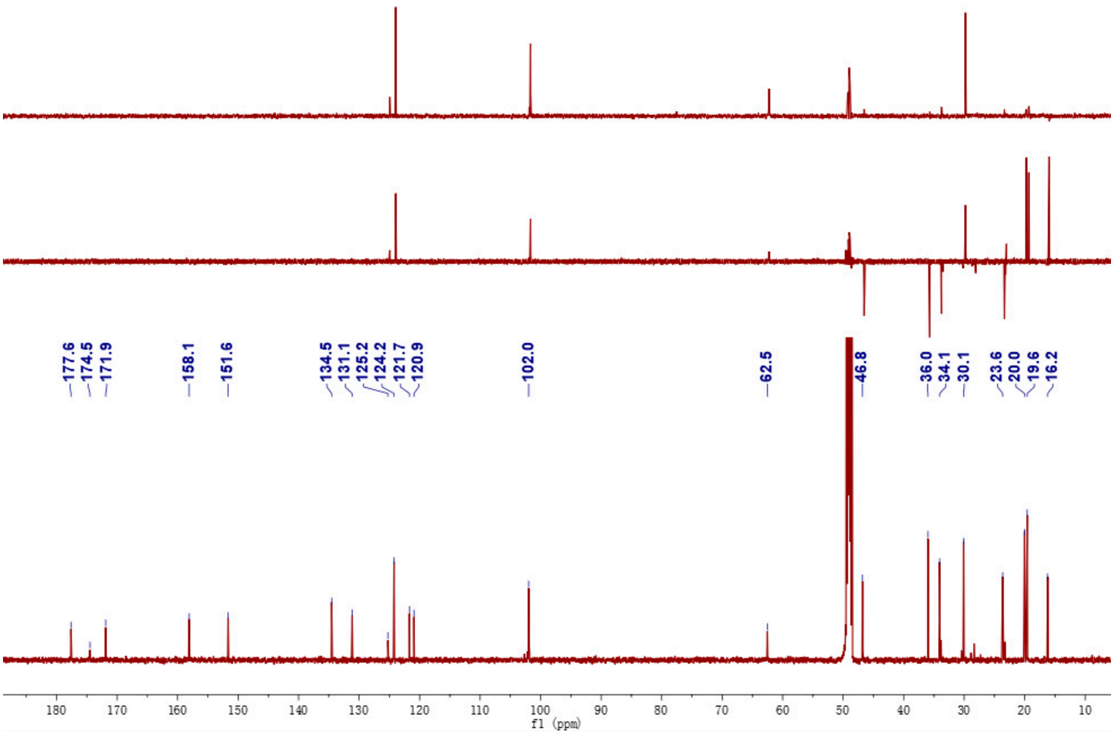

Figure S60. ^{1}H-^{1}H COSY spectrum of compound 9.

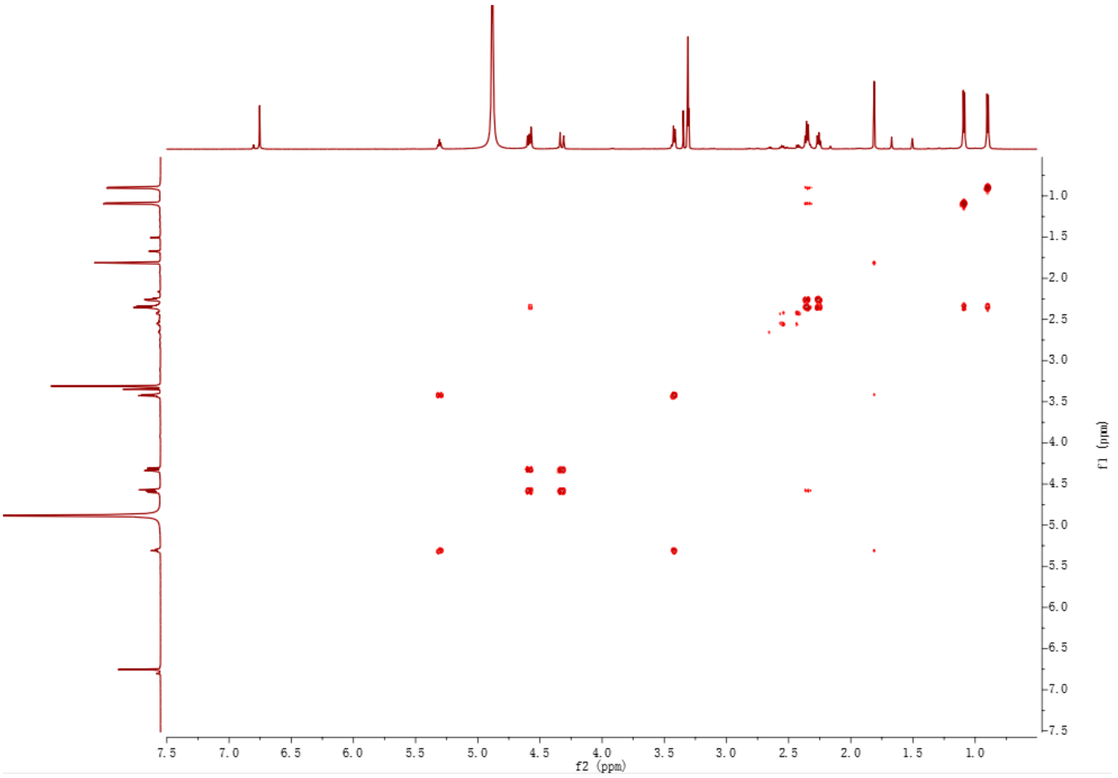

Figure S61. HSQC spectrum of compound 9.

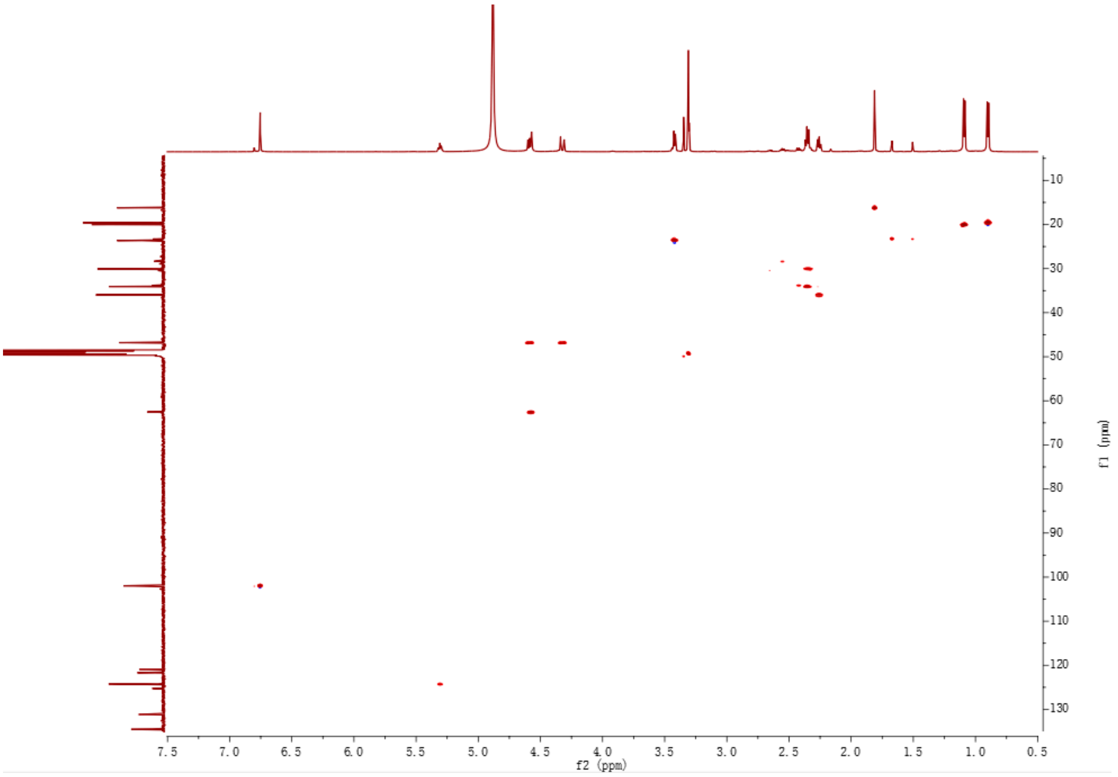

Figure S62. HMBC spectrum of compound 9.

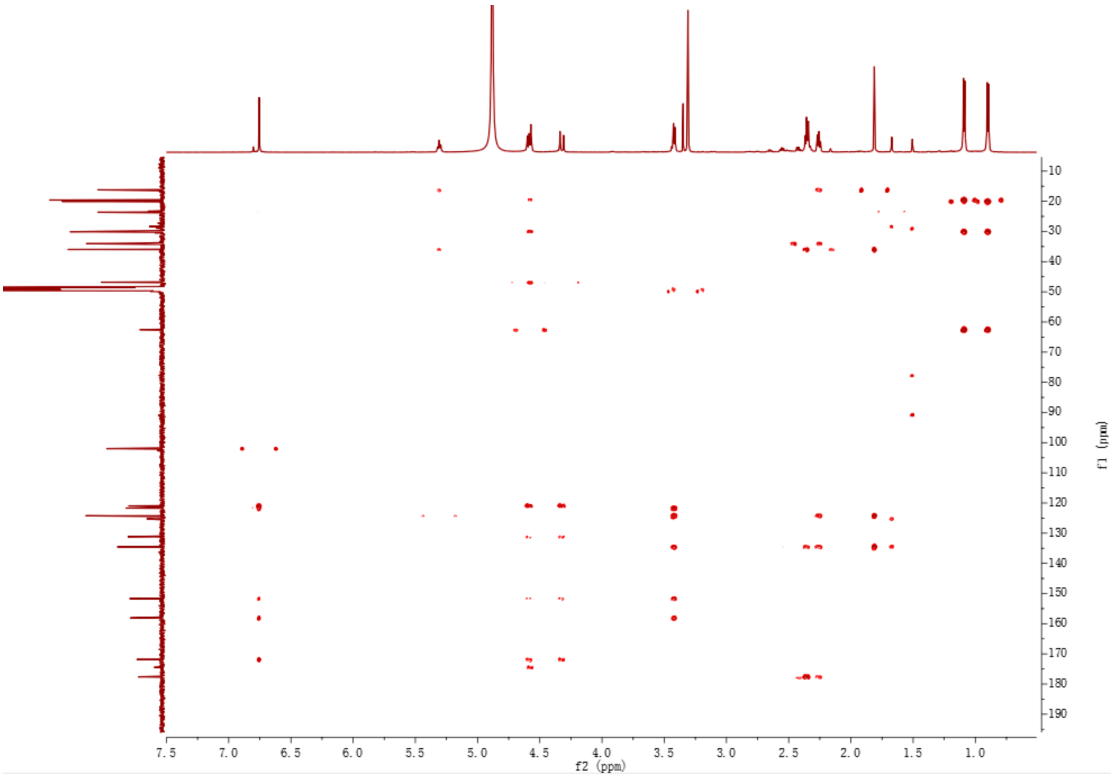

Figure S63. ROESY spectrum of compound 9.

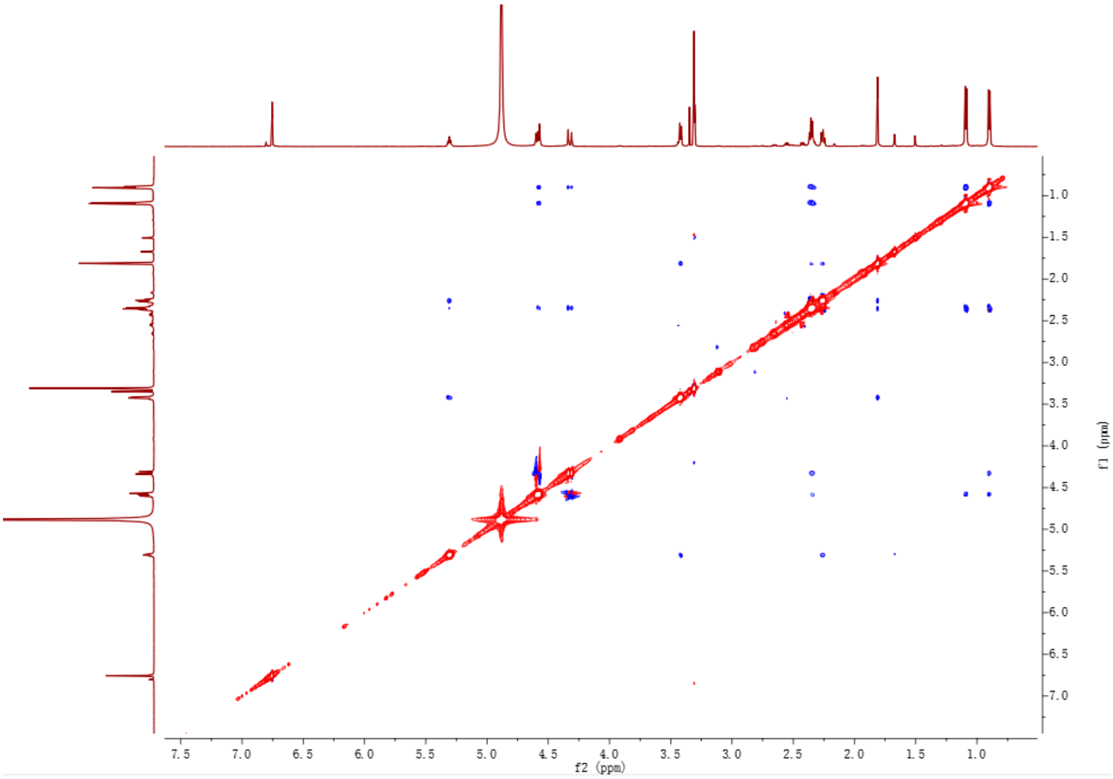

Figure S64. HRESIMS spectroscopic data of compound 10.

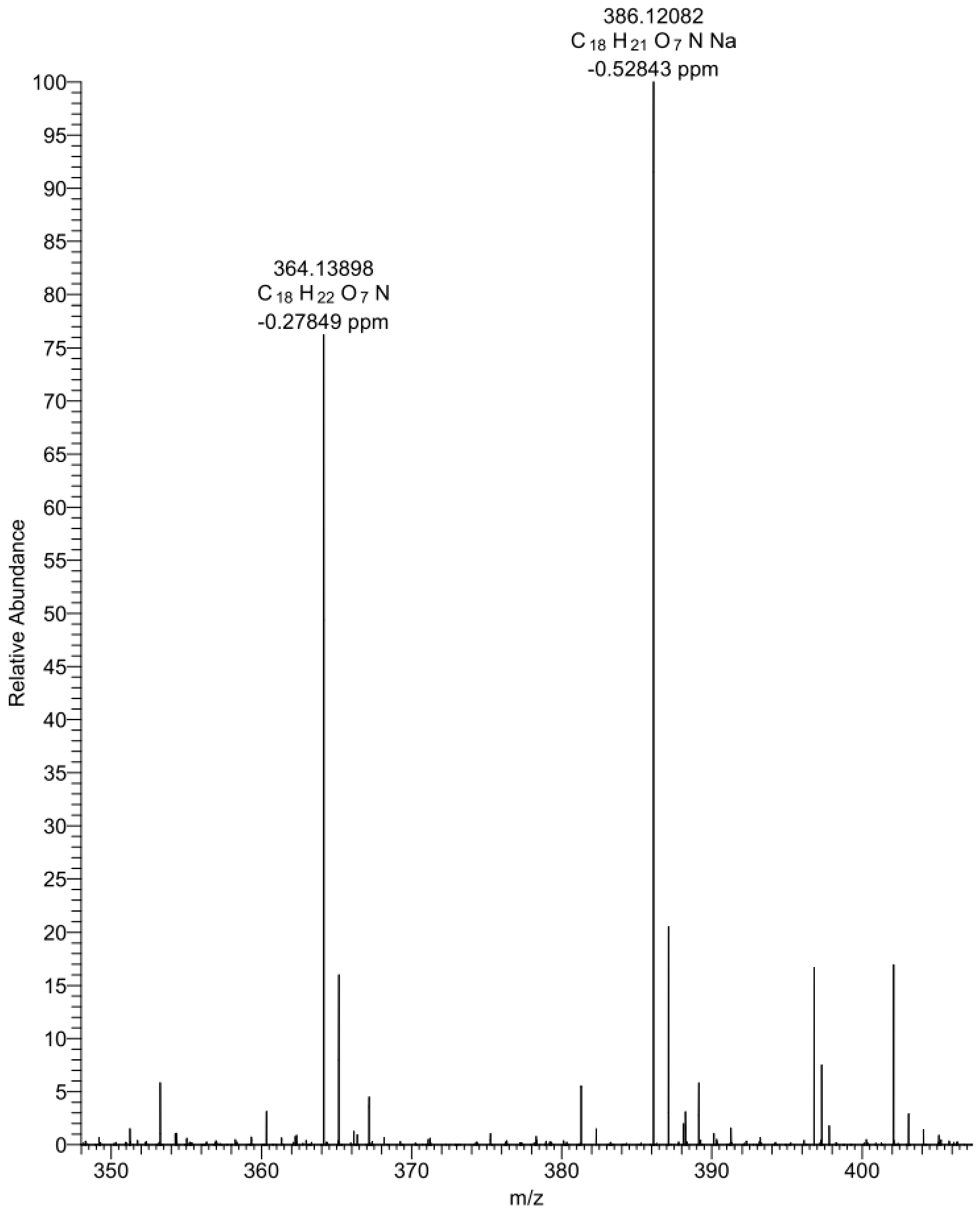

Figure S65. ^{1}H NMR (600 MHz) spectrum of compound 10.

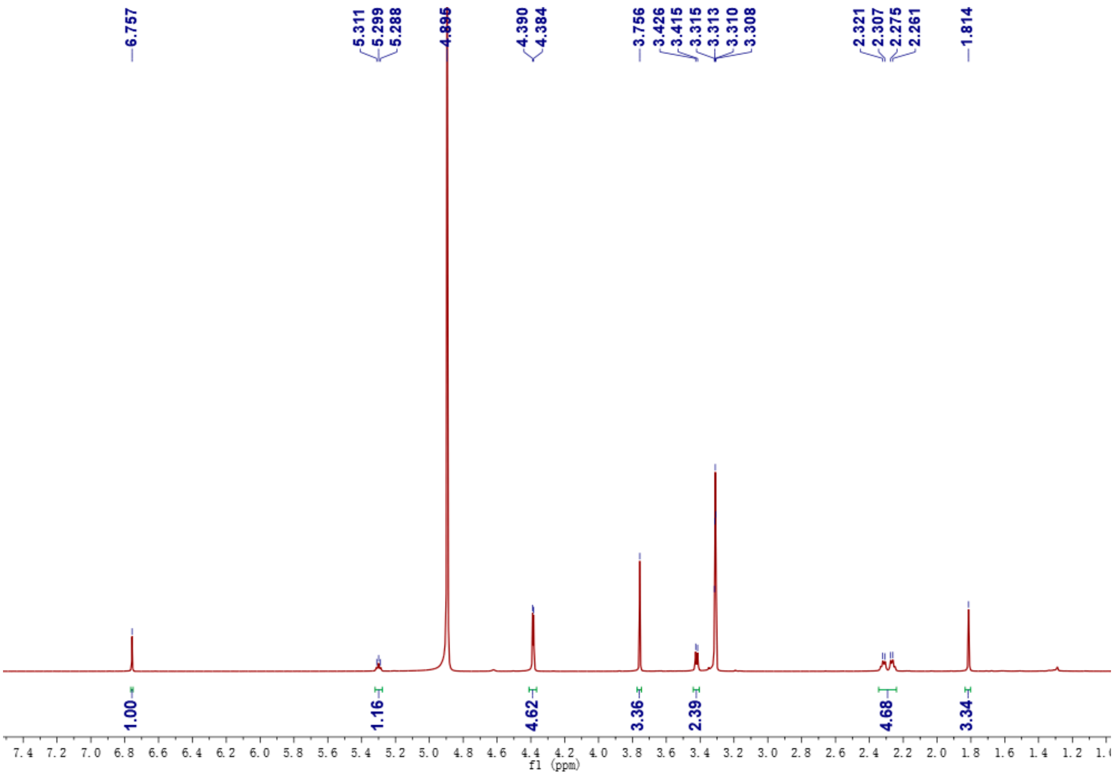

Figure S66. ^{13}C NMR and DEPT (150 MHz) spectra of compound 10.

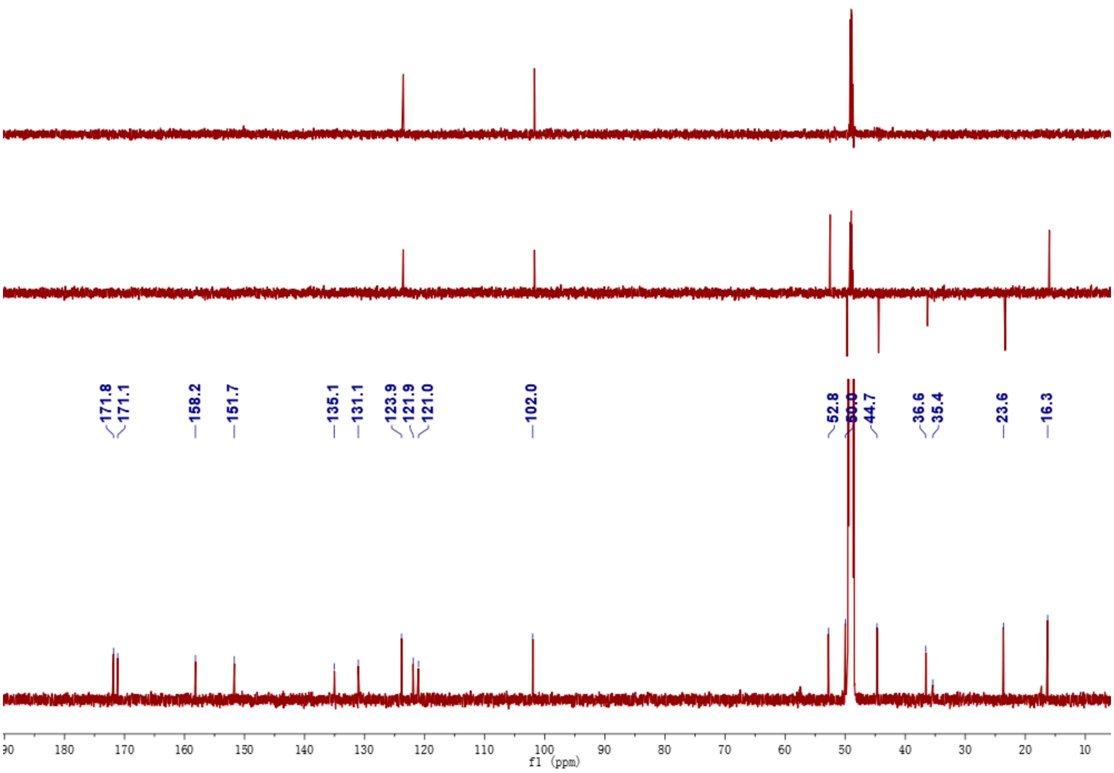

Figure S67. ^{1}H-^{1}H COSY spectrum of compound 10.

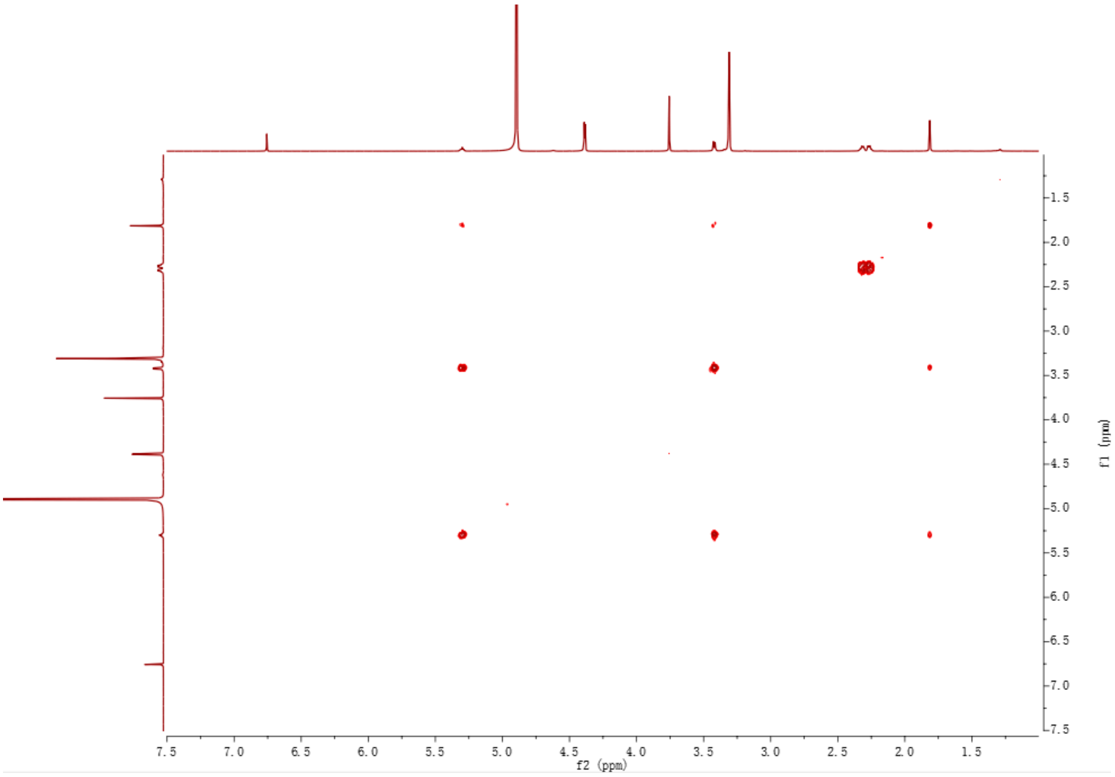

Figure S68. HSQC spectrum of compound 10.

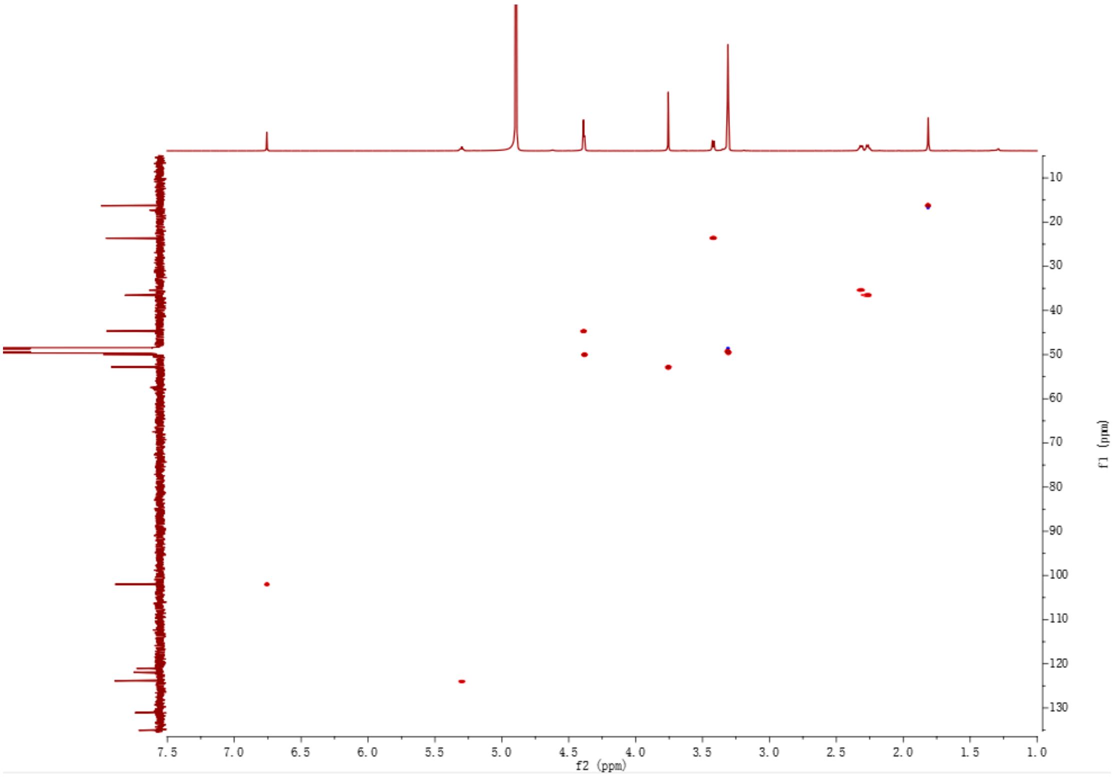

Figure S69. HMBC spectrum of compound 10.

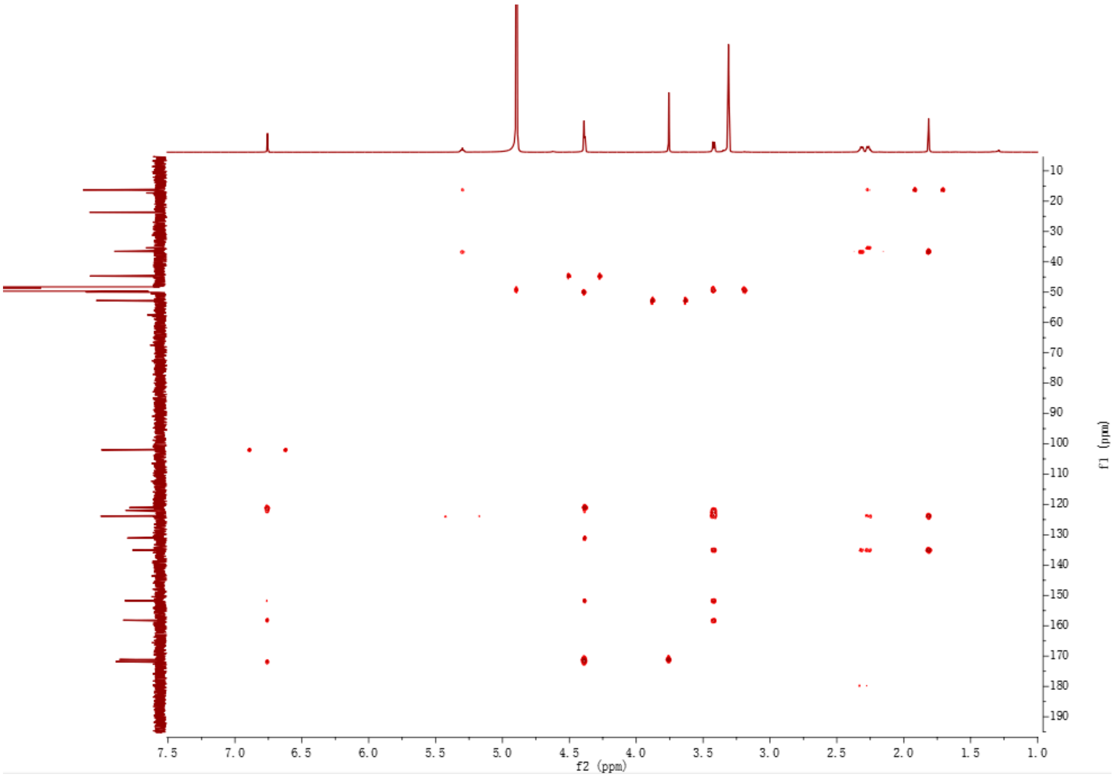

Figure S70. ROESY spectrum of compound 10.

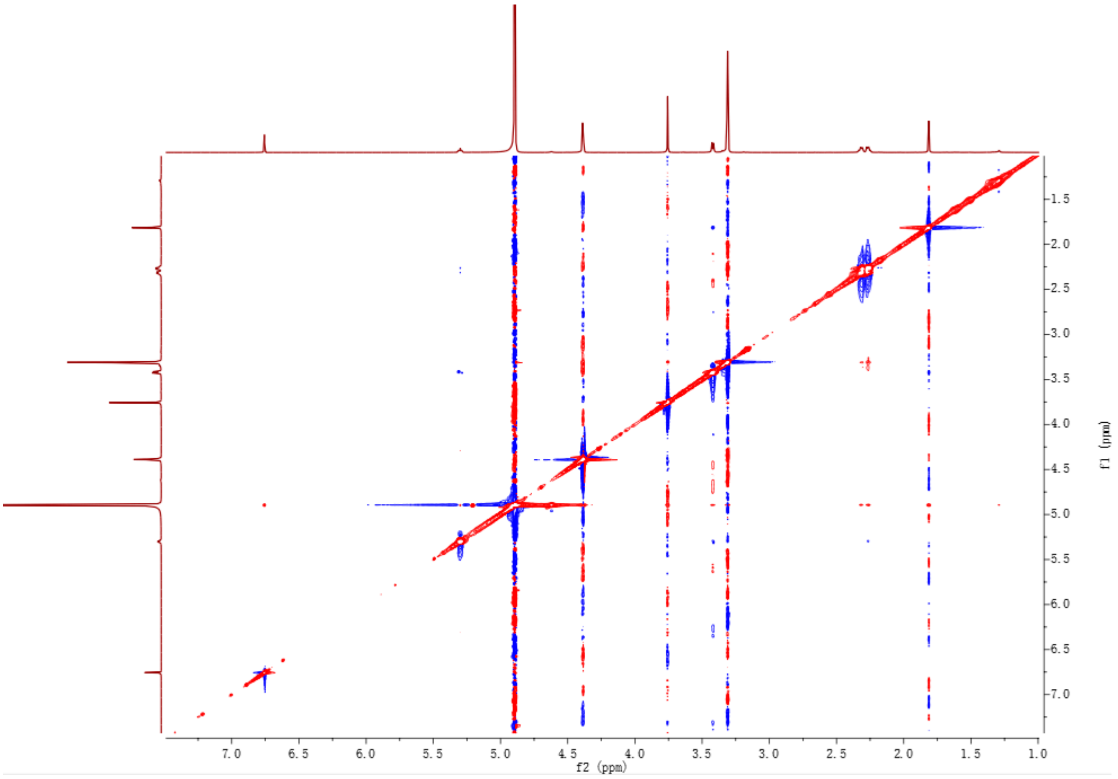

Figure S71. HRESIMS spectroscopic data of compound 11.

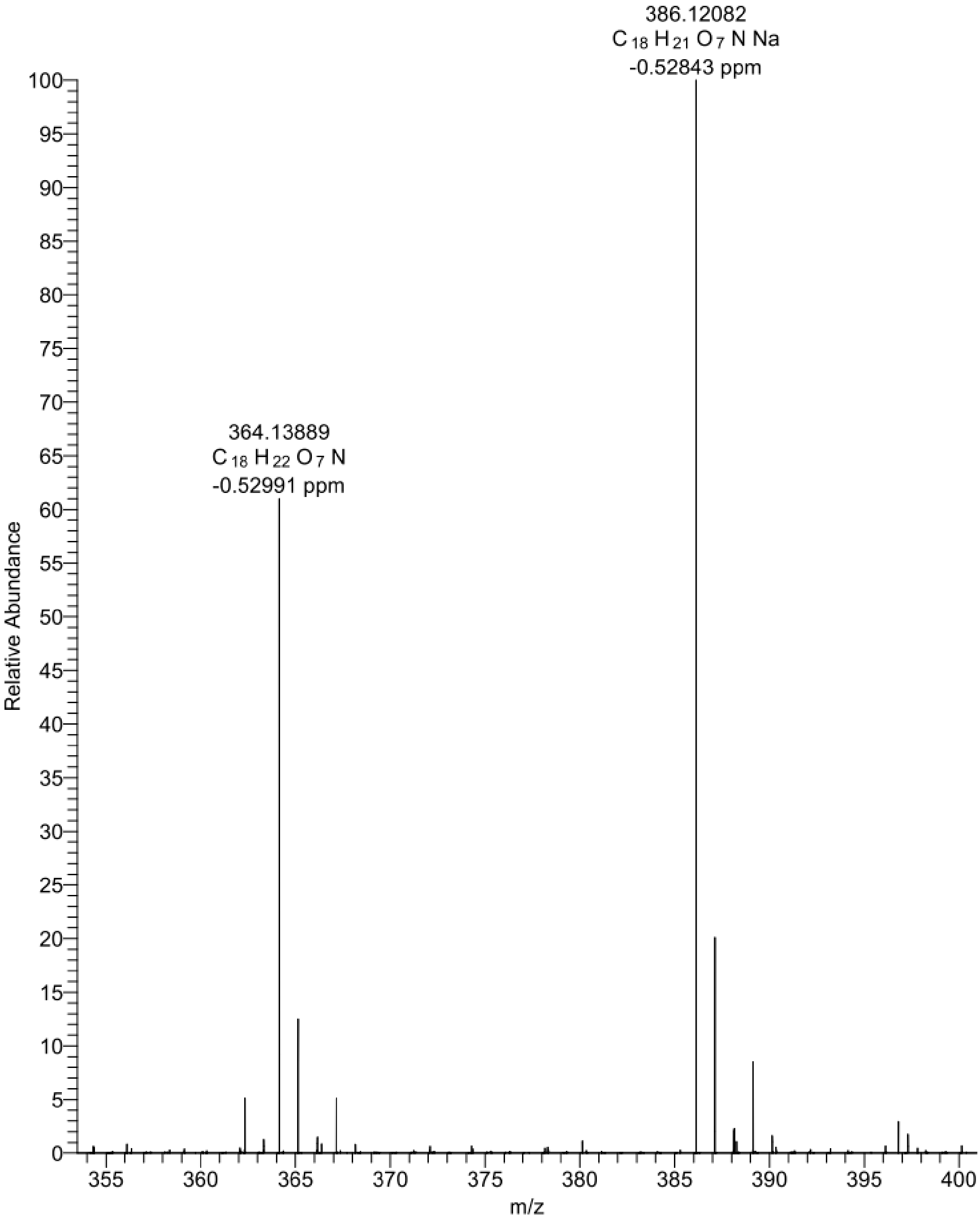

Figure S72. ^{1}**H NMR (600 MHz) spectrum of compound 11.**

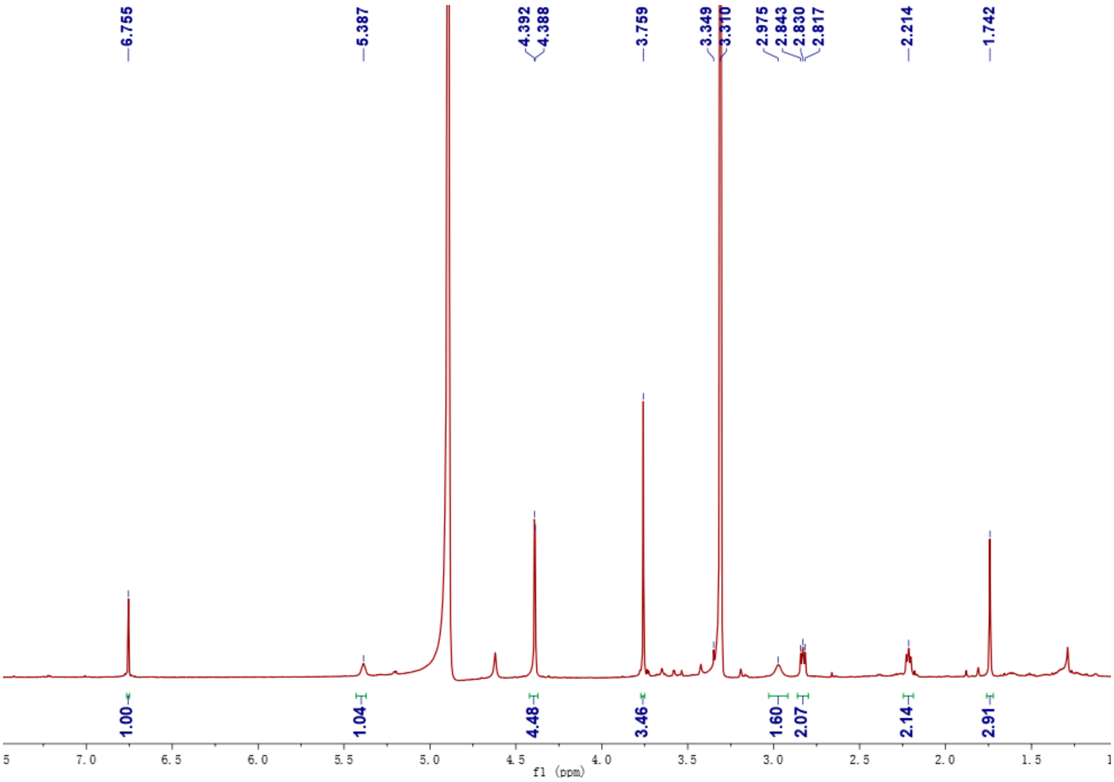

Figure S73. ^{13}**C NMR and DEPT (150 MHz) spectra of compound 11.**

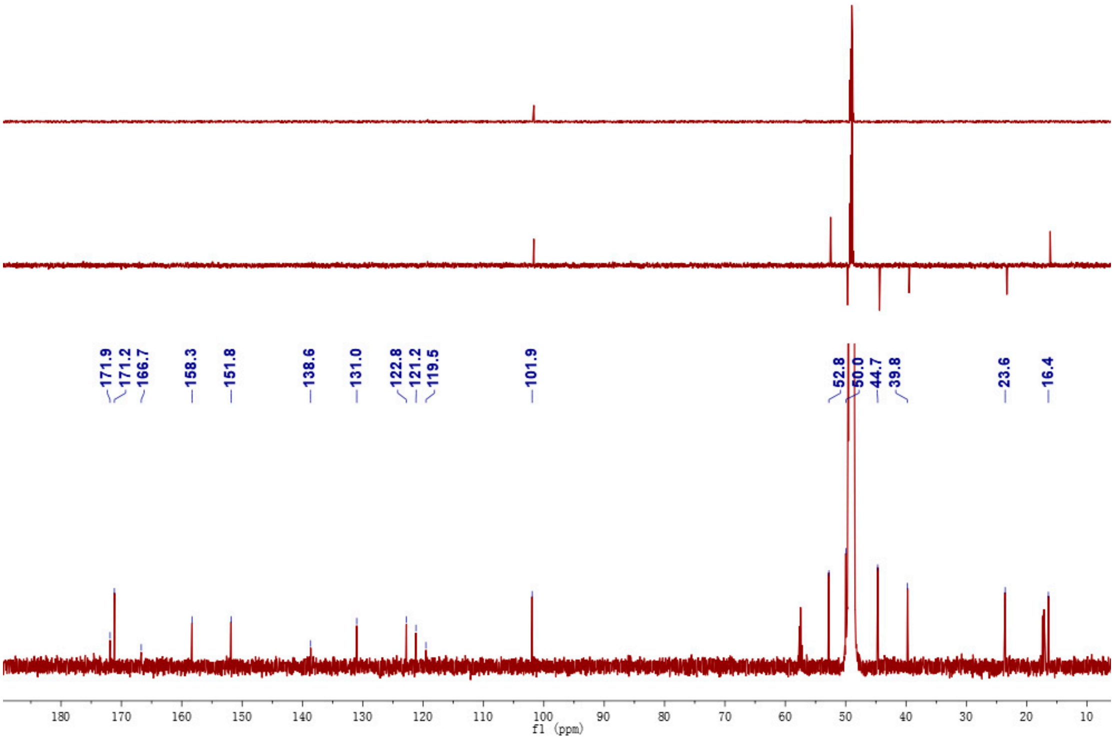

Figure S74. ^{1}H-^{1}H COSY spectrum of compound 11.

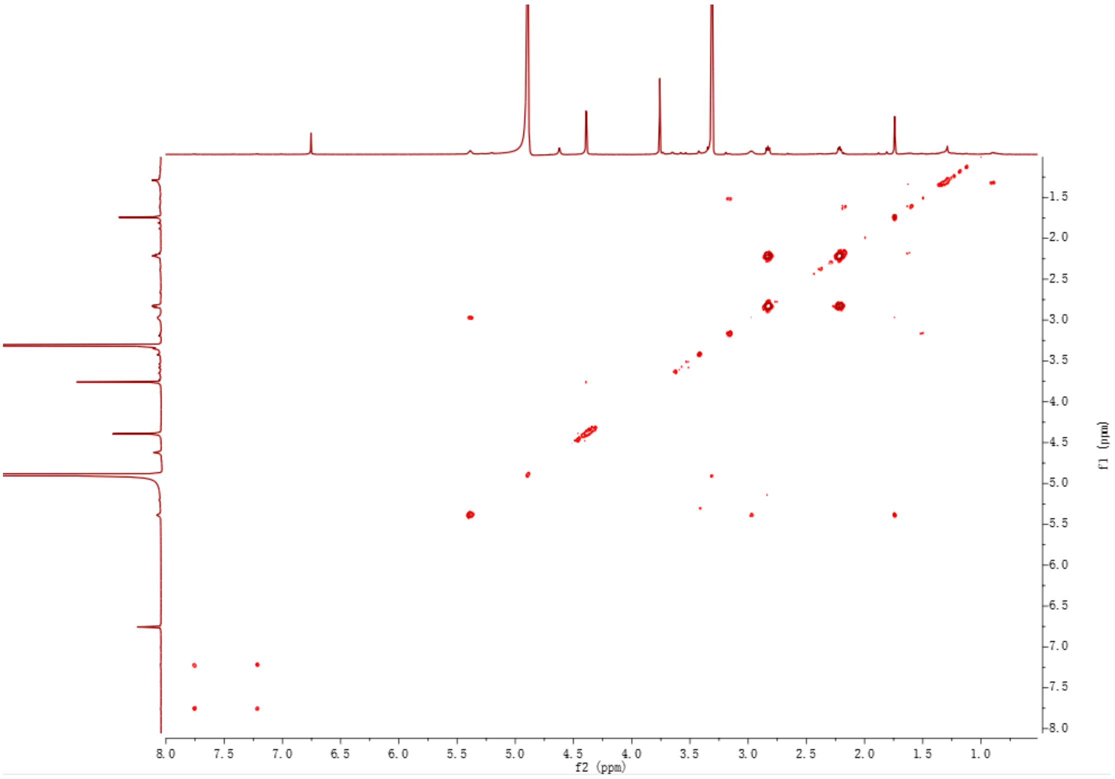

Figure S75. HSQC spectrum of compound 11.

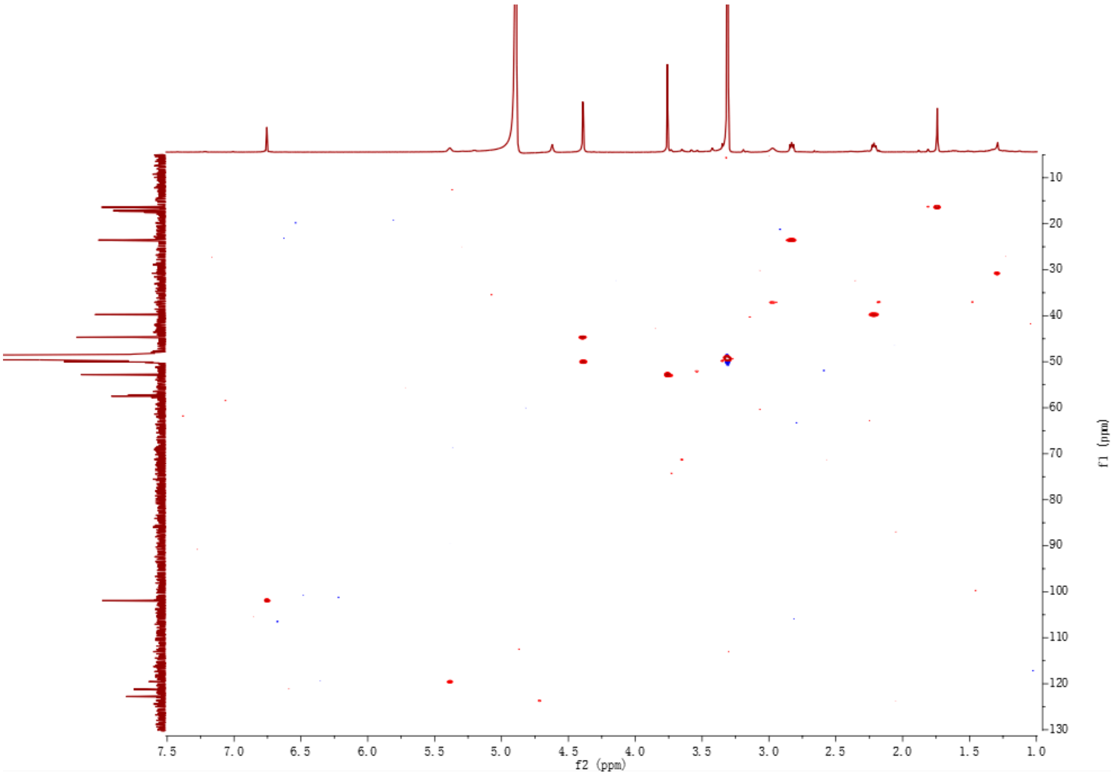

Figure S76. HMBC spectrum of compound 11.

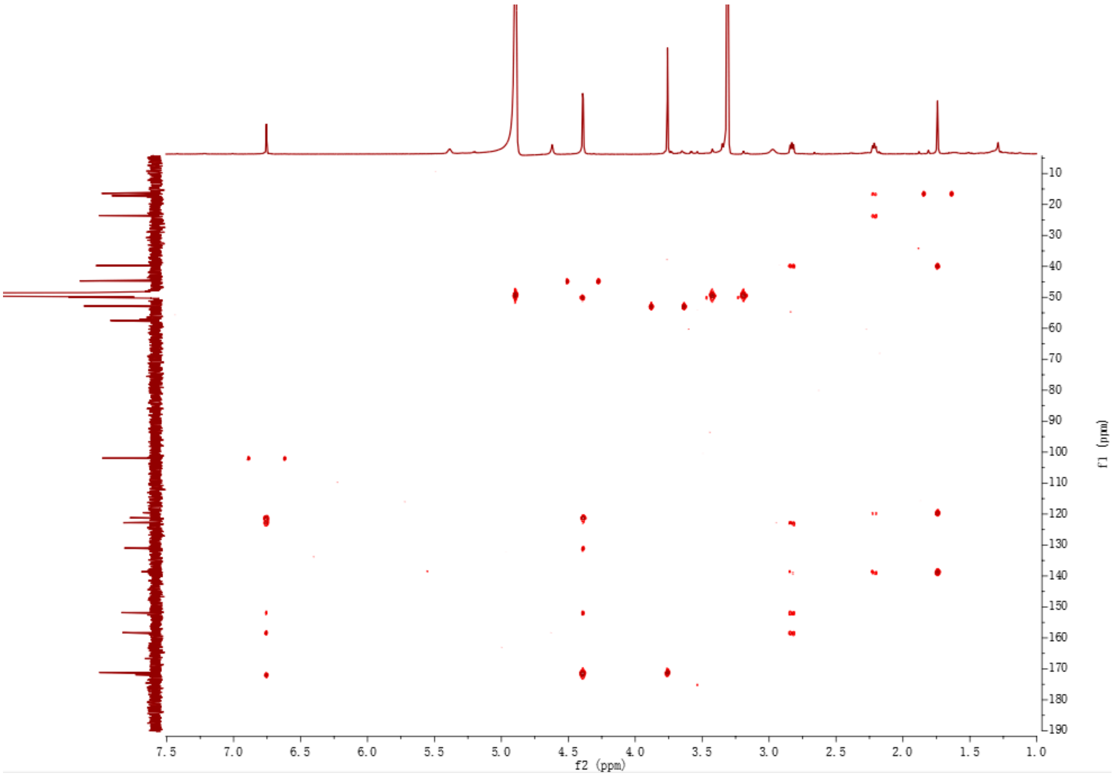

Figure S77. ROESY spectrum of compound 11.

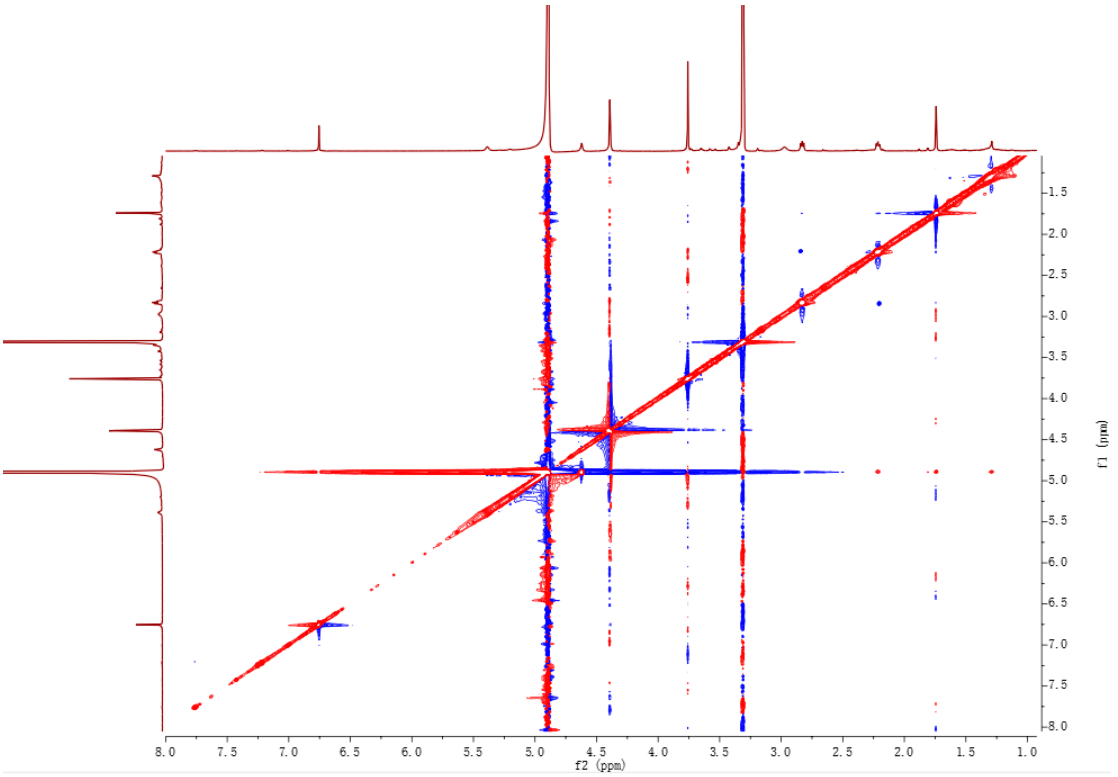

Figure S78. HRESIMS spectroscopic data of compound 12.

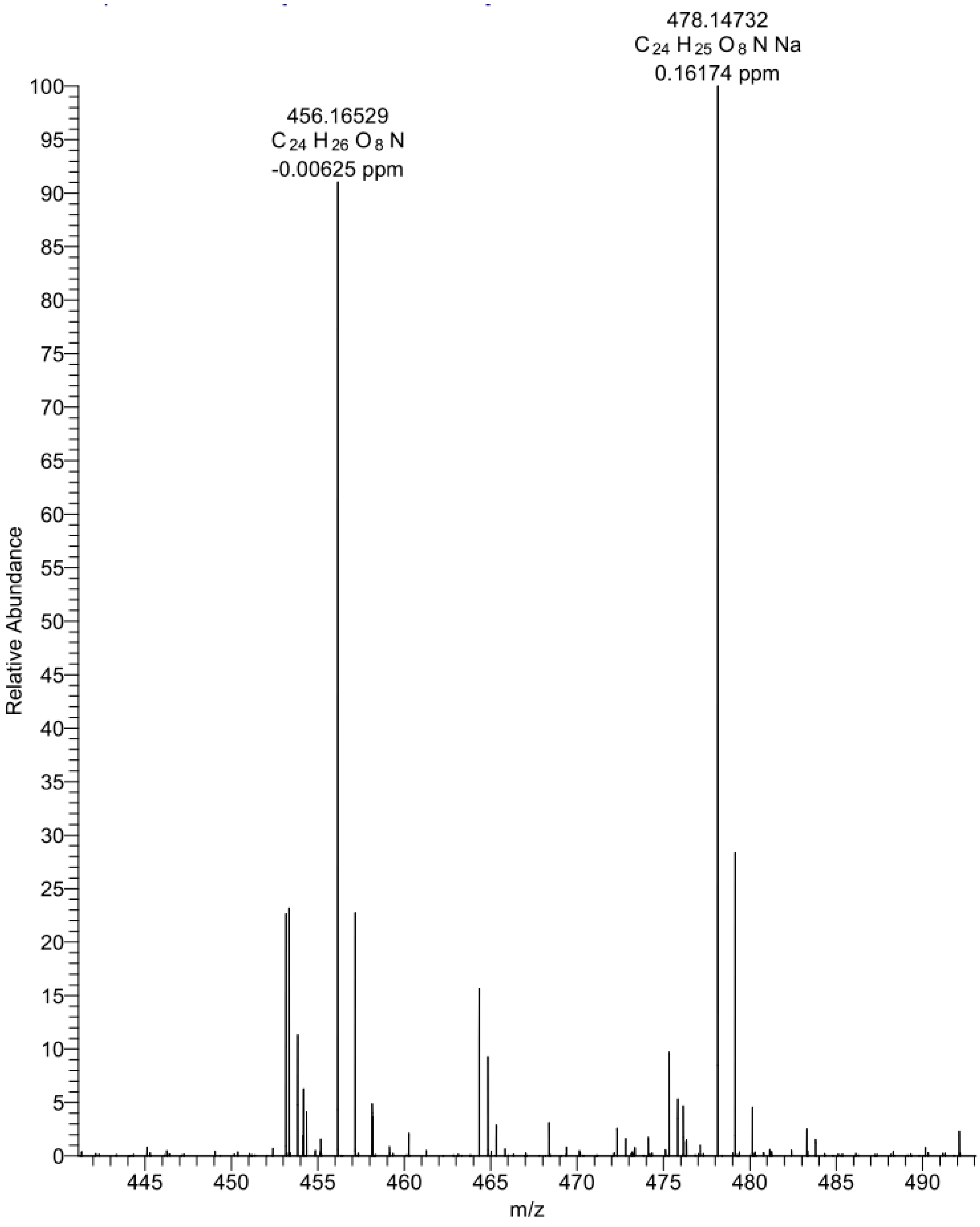

Figure S79. ^{1}H NMR (500 MHz) spectrum of compound 12.

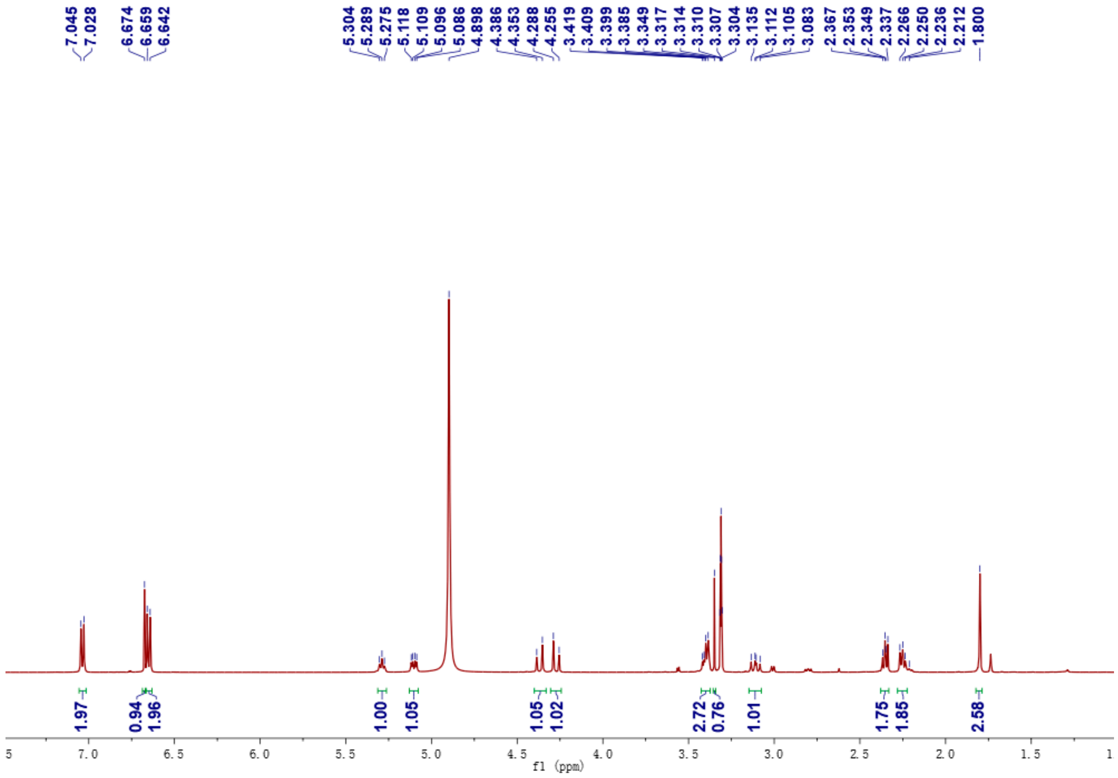

Figure S80. ^{13}C NMR and DEPT (125 MHz) spectra of compound 12.

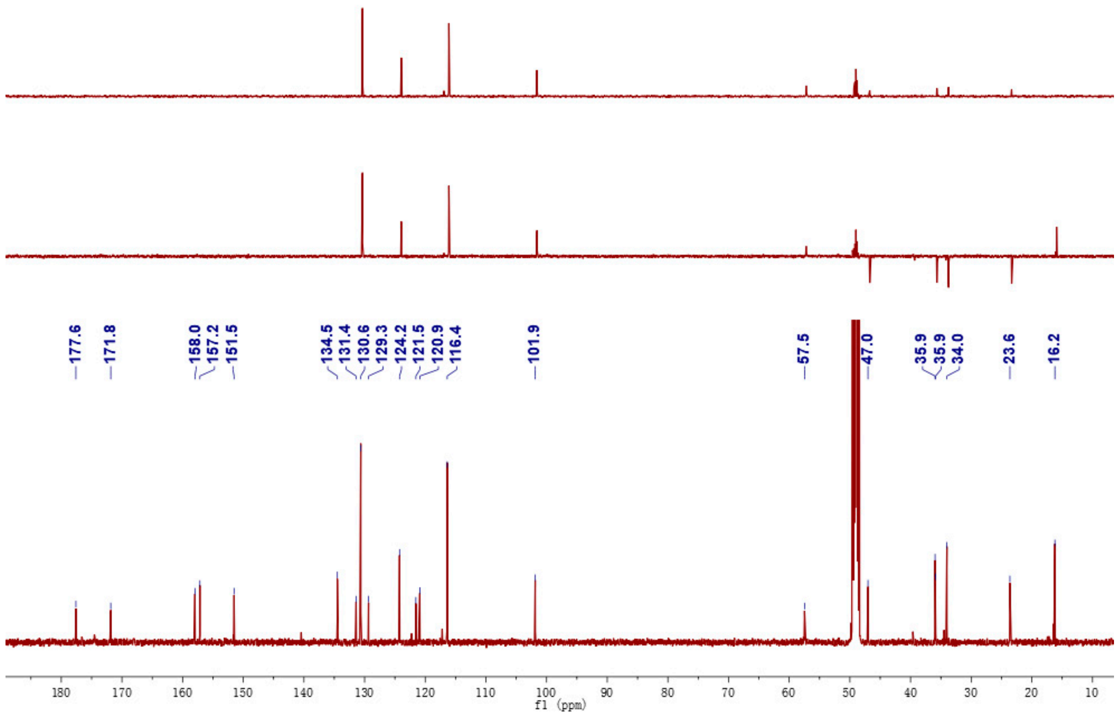

Figure S81. ^1H-^1H COSY spectrum of compound 12.

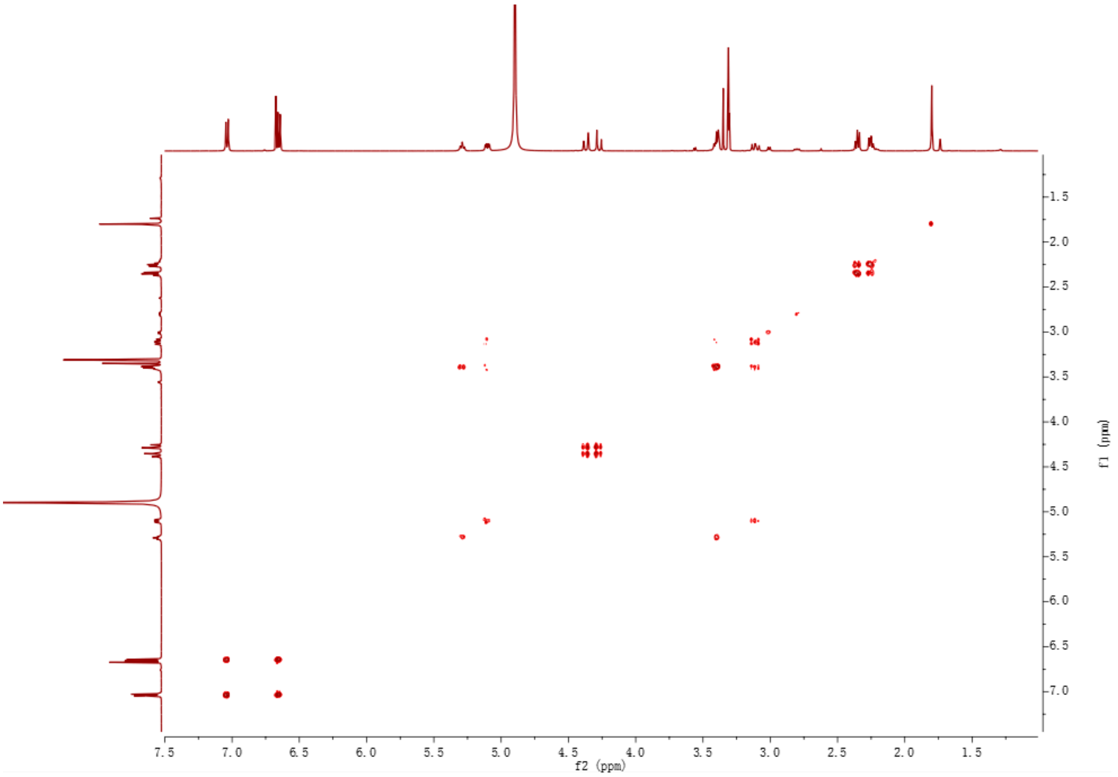

Figure S82. HSQC spectrum of compound 12.

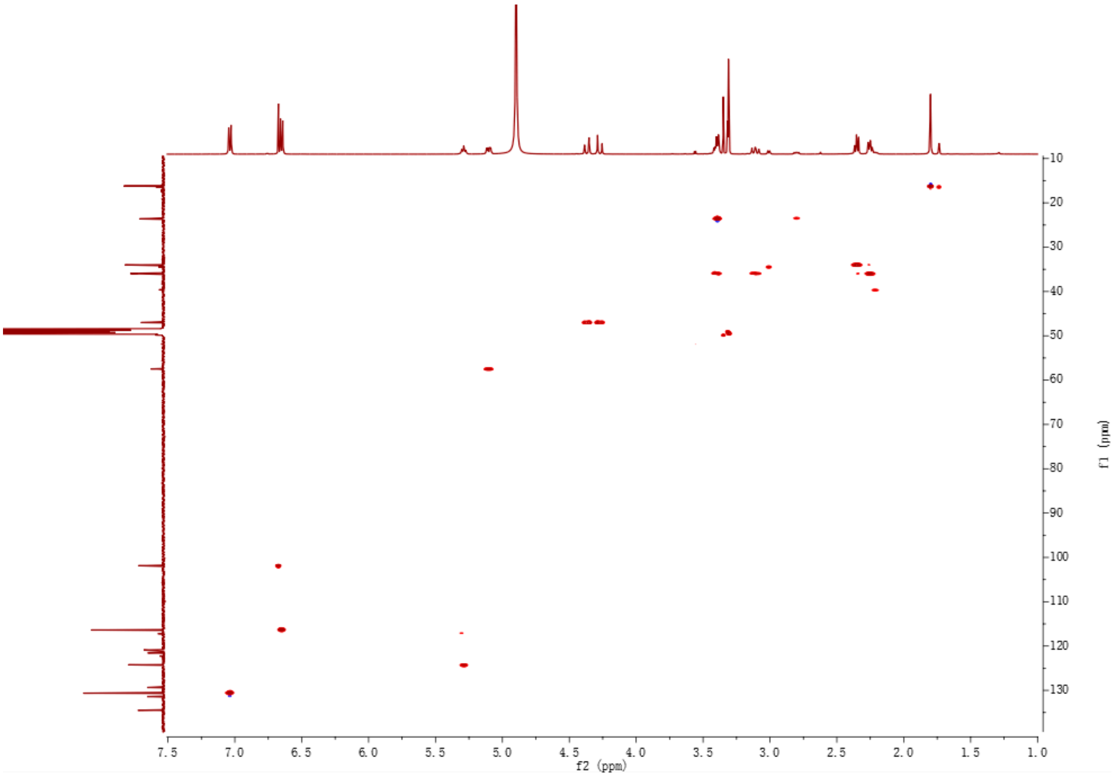

Figure S83. HMBC spectrum of compound 12.

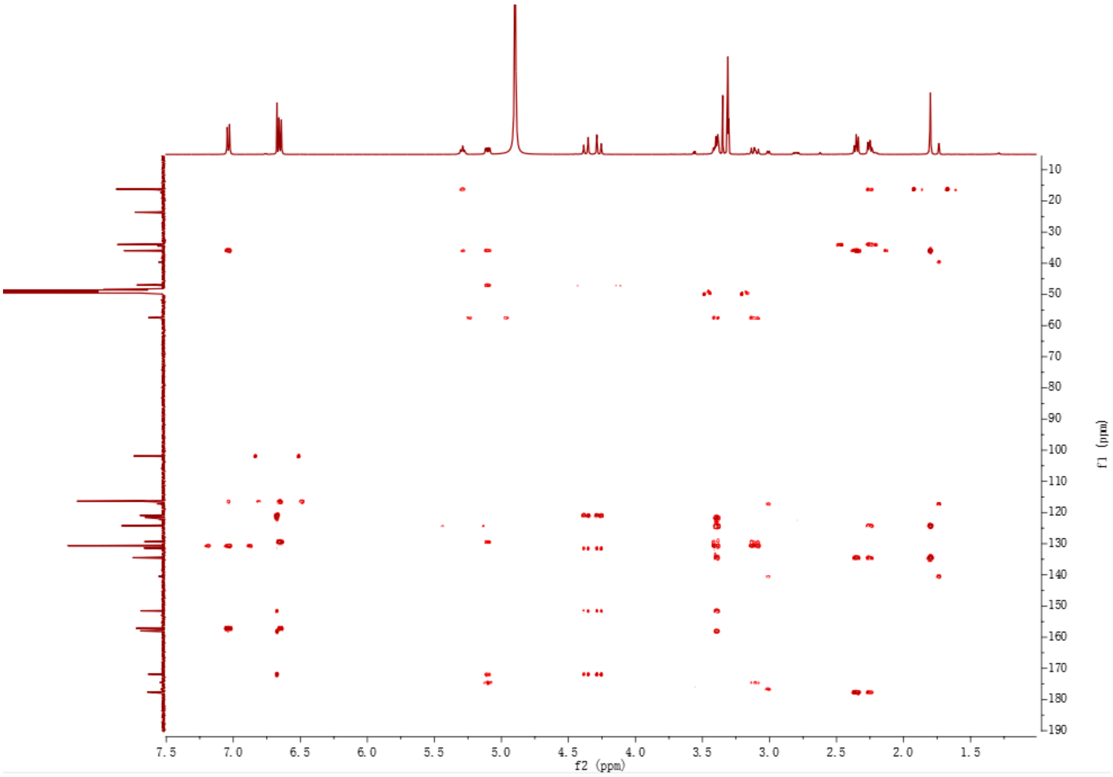

Figure S84. ROESY spectrum of compound 12.

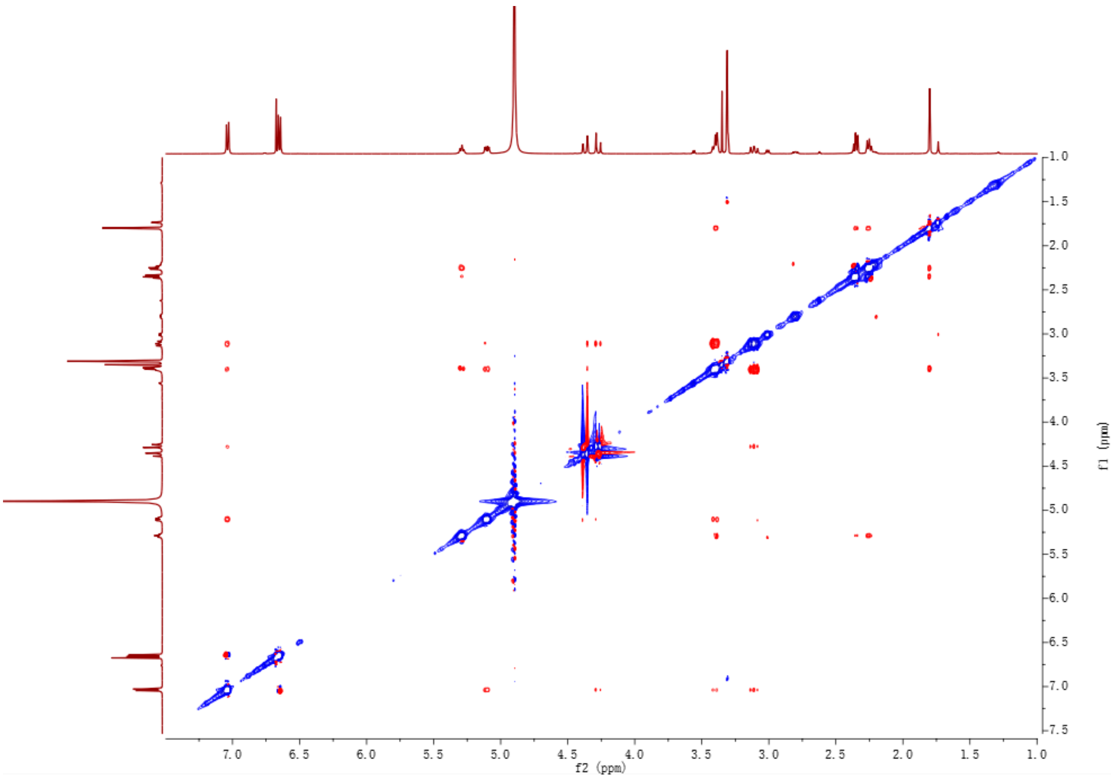

Supplement: Supplementary file 1 [file molecules-29-04901-s001.zip › molecules-3254323-supplementary.pdf]
